# Specific inhibition of the Survivin–CRM1 interaction by peptide-modified molecular tweezers

**DOI:** 10.1038/s41467-021-21753-9

**Published:** 2021-03-08

**Authors:** Annika Meiners, Sandra Bäcker, Inesa Hadrović, Christian Heid, Christine Beuck, Yasser B. Ruiz-Blanco, Joel Mieres-Perez, Marius Pörschke, Jean-Noël Grad, Cecilia Vallet, Daniel Hoffmann, Peter Bayer, Elsa Sánchez-García, Thomas Schrader, Shirley K. Knauer

**Affiliations:** 1 Department of Molecular Biology II, Centre for Medical Biotechnology (ZMB), University of Duisburg-Essen, Essen, Germany; 2 Institute of Organic Chemistry I, Faculty of Chemistry, University of Duisburg-Essen, Essen, Germany; 3 Department of Structural and Medicinal Biology, Centre for Medical Biotechnology (ZMB), University of Duisburg-Essen, Essen, Germany; 4 Department of Computational Biochemistry, Centre for Medical Biotechnology (ZMB), University of Duisburg-Essen, Essen, Germany; 5 Department of Bioinformatics and Computational Biophysics, Centre for Medical Biotechnology (ZMB), University of Duisburg-Essen, Essen, Germany

**Keywords:** Cancer, Chemical biology, Molecular biology, Chemistry

## Abstract

Survivin’s dual function as apoptosis inhibitor and regulator of cell proliferation is mediated via its interaction with the export receptor CRM1. This protein–protein interaction represents an attractive target in cancer research and therapy. Here, we report a sophisticated strategy addressing Survivin’s nuclear export signal (NES), the binding site of CRM1, with advanced supramolecular tweezers for lysine and arginine. These were covalently connected to small peptides resembling the natural, self-complementary dimer interface which largely overlaps with the NES. Several biochemical methods demonstrated sequence-selective NES recognition and interference with the critical receptor interaction. These data were strongly supported by molecular dynamics simulations and multiscale computational studies. Rational design of lysine tweezers equipped with a peptidic recognition element thus allowed to address a previously unapproachable protein surface area. As an experimental proof-of-principle for specific transport signal interference, this concept should be transferable to any protein epitope with a flanking well-accessible lysine.

## Abstract (web summary)

Survivin’s dual function as apoptosis inhibitor and regulator of cell proliferation is mediated via its interaction with the export receptor CRM1. Here authors report a strategy addressing its dimer interface overlapping with the nuclear export signal, the binding site of CRM1, with advanced supramolecular tweezers for lysine and arginine.

## Introduction

Protein–protein interactions (PPIs) have enormous importance for numerous biological processes, and are relevant to understand protein function, assembly, and communication. Today, large efforts and resources are focused on unveiling the wide interactome between more than 200,000 human proteins encoded in our genome^1^. The deliberate modulation of PPIs with external agents opens opportunities to study biological mechanisms and to interfere with pathological processes^1,2^.

However, interfaces between proteins are very difficult targets for molecular recognition since such interfaces are usually large (>1000 Å^2^), well solvated, and display a rugged topology^3^. Thus, until today interfering molecules have in most cases been identified by extensive library screening.

Supramolecular chemistry provides orthogonal artificial elements for protein recognition and, in combination with computational modeling, allows a deeper understanding of the underlying noncovalent interactions^4^. Inter alia, calixarenes^5^, cucurbiturils^6^, molecular tweezers^7^, and GCP motifs^8^ recognize well-solvated amino acid residues on protein surfaces and have been successfully used to target protein surfaces and to interfere with PPIs^9–13^. On the same protein, i.e., ubiquitin, a recent comparative study revealed that these host molecules occupy different areas and seem to exhibit complementary recognition profiles^14^.

Despite these promising features and first applications in cells and animals, the selective recognition of protein elements by supramolecular host systems remains highly challenging. A few recent examples include synthetic ligands for peptide motifs on proteins^15^ and for a specific protein context^16,17^.

We here present an advanced approach by combination of a supramolecular host molecule with a well-defined biomolecular interaction. This ditopic hybrid allows us to complex a single critical amino acid together with its direct environment on the protein surface, which leads to powerful competition with the natural binding partner. Specifically, we generate a covalent conjugate between a lysine-selective molecular tweezer and a self-complementary peptide and target a critical interface important for the survival of cancer cells, i.e., the nuclear export signal (NES), located on an ordered but somewhat dynamic loop on the Survivin surface.

Survivin is mostly absent in normal resting adult tissues, but highly upregulated in almost all cancer types^18,19^. Its overexpression is associated with resistance against chemo- and radiotherapy, frequent recurrences, and a decreased patient survival^20–23^. Despite its small size (142 aa, 16.5 Da) and its lack of enzymatic activity, Survivin is fulfilling a well characterized dual role within the cell^24^. As the smallest member of the inhibitor of apoptosis protein family, Survivin on the one hand plays a role in counteracting programmed cell death. As part of the chromosomal passenger complex (CPC), Survivin is on the other hand crucial for mitotic regulation promoting cell proliferation^25^. For both functions, an interaction with the nuclear export receptor CRM1 mediated by Survivin’s highly conserved, leucine-rich NES is pivotal^26–28^. Thus, interference with the Survivin–CRM1 interaction can inhibit cancer cell proliferation.

The development of a small molecule, which specifically binds to the NES on Survivin’s surface, would represent a valuable approach to inhibit the Survivin–CRM1 interaction. This is a challenge for supramolecular chemistry: can we direct an amino acid-selective host molecule to the functionally relevant epitope on Survivin by combining it with a recognition unit for the NES? This key region is located on an ordered loop and flanked by well-accessible basic amino acids, K90/91 and K103/R106. We therefore designed supramolecular tweezers that only target lysine (Lys) and arginine (Arg) residues and that are equipped with a compact binder to the natural dimer interface overlapping with the NES.

Molecular tweezers possess a torus-shaped arrangement of alternating benzene and norbornadiene rings, which form an electron-rich unpolar cavity—ideally suited to pull the cationic side chains of Lys and Arg inside. Two (hydrogen)phosphate groups lock the included side chain in an ion pair^29^. This unique binding mechanism operates well under physiological conditions and has already been exploited for protease inhibition^30,31^, prevention of protein aggregation^32,33^, and modulation of PPIs on shallow grooves^7,34^. In order to turn these molecular tools into specific Survivin ligands, it was necessary to identify a binding motif for the NES region and to develop a synthesis for efficient tweezer monofunctionalization. This would allow us to attach the NES binder covalently to the tweezer and generate the desired ditopic ligand.

In this work, we thus rationally equipped molecular tweezers for lysine and arginine with small peptides resembling the natural dimer interface in order to shield the NES from binding of cognate receptor CRM1. We demonstrate and rationalize the binding of the tweezers to Survivin and the interference with this critical PPI via several biochemical methods combined with molecular dynamics (MD) simulations and multiscale computational studies. Indeed, conjugation of an elongated peptide sequence (ELTLGEFL) outmatched a shorter and a scrambled, unselective peptide with regard to binding and inhibition potential. As such, with our rationally developed peptide-modified tweezers, specific supramolecular inhibitors of so far not easily targetable protein surface structures are now available.

## Results

### Development of peptide-modified molecular tweezers

**Fig. 1:**
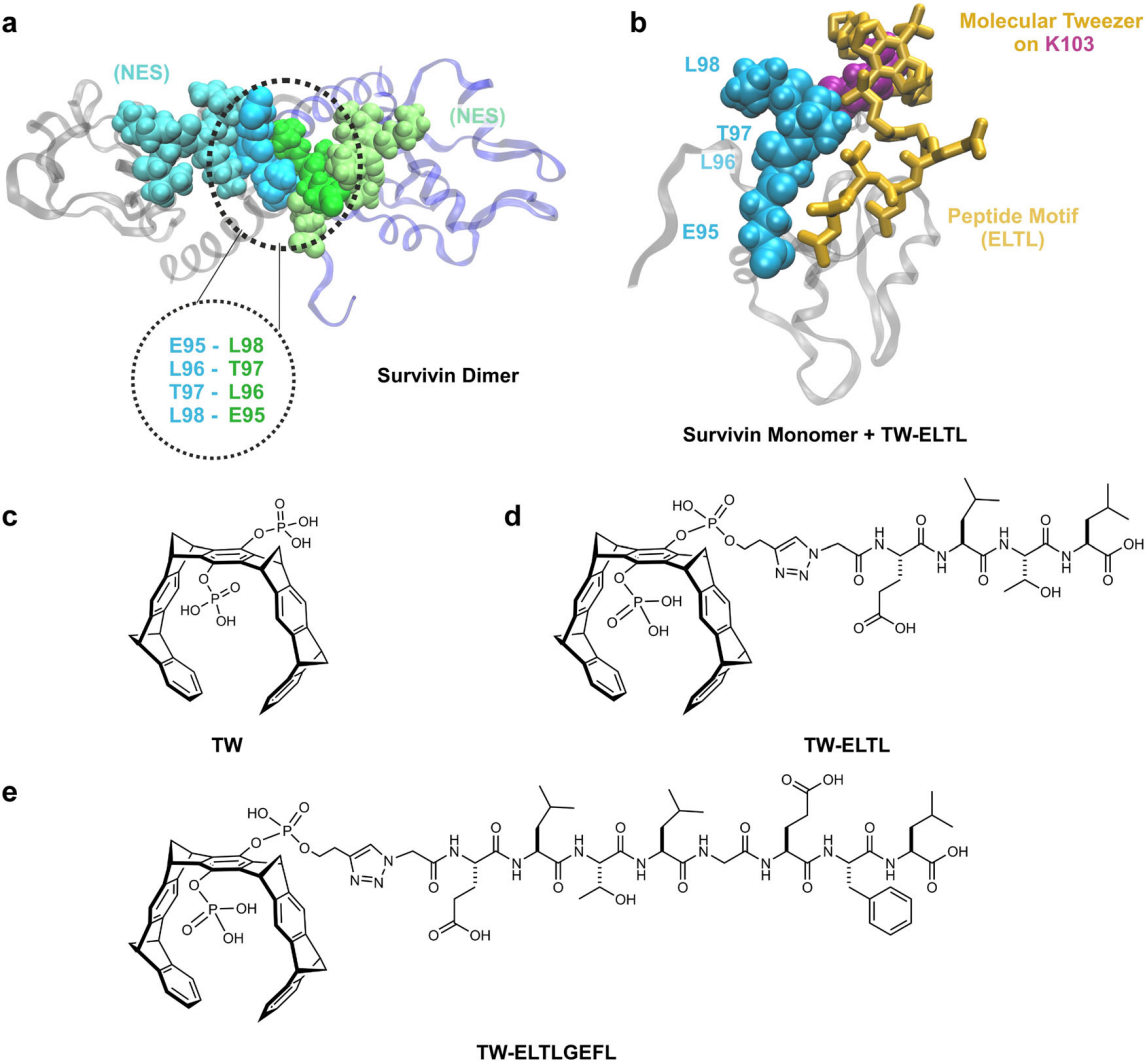
Design of peptide-modified supramolecular tweezers. **a** Representation of Survivin’s dimer interface based on PDB-ID: 1XOX [https://www.rcsb.org/structure/1xox]^38^. Both monomers, depicted in blue and gray, mainly interact via the ELTL sequence (contact region of both monomers overlapping with the NES, represented in cyan and green). This sequence was chosen as second binding motif for the peptide-modified tweezer molecules. **b** Representation of TW-ELTL (shown in **d**) bound to Survivin. TW-ELTL (yellow) binds the anchor lysine residue K103 (violet) on Survivin’s surface while the peptide motif ELTL (yellow) interacts with the ELTL region of the Survivin monomer (cyan). This is the same region of the dimer interface represented in Fig. 1a, overlapping with the NES (cyan). The chemical structures of the unmodified tweezer molecule TW (**c**), an asymmetrical tweezer molecule linked to the short peptide ELTL (TW-ELTL) (**d**), and an asymmetrical tweezer molecule linked to the elongated peptide ELTLGEFL (TW-ELTLGEFL) (**e**) are depicted.

**Fig. 2:**
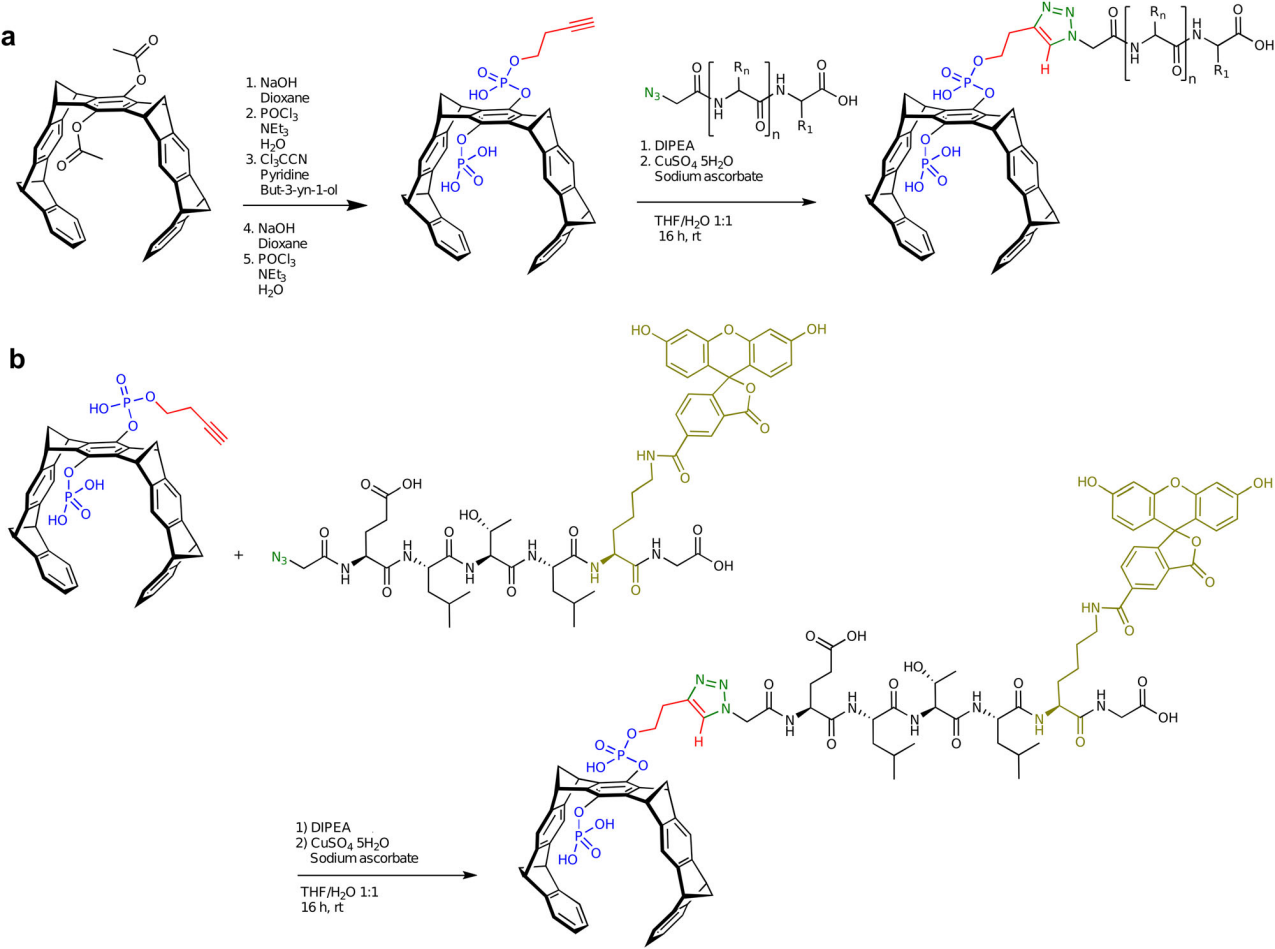
Synthetic strategy leading to monofunctionalized molecular tweezers. **a** Introduction of one butynyl phosphate ester arm on the parent diacetoxy tweezer (TCA coupling) followed by click chemistry with N-terminal azidopeptide; **b** additional introduction of a fluorescence label by adding a C-terminal Lys^FAM^-Gly sequence to the clicked peptide. Blue: phosphate; red: alkyne/triazole; green: azide/triazole; olive: FAM label. DIPEA N,N-diisopropylethylamine, THF tetrahydrofuran.

In the absence of other binding partners, the monomer–dimer equilibrium of Survivin lies on the dimer side; the functional interface (^93^FEELTLGEFL^102^) between both protomers is self-complementary with the hydrophobic interactions of entangled leucines playing a crucial role^35,36^. Intriguingly, this interface largely overlaps with Survivin’s NES (^89^VKKQFEELTL^98^). Hence, peptide fragments from the dimer interface (Fig. 1 a) are ideal candidates for the desired additional recognition unit. We therefore selected a short (^95^ELTL^98^) and an elongated peptide (^95^ELTLGEFL^102^) taken directly from this dimer interface (Fig. 1b–e). A synthetic strategy employing click chemistry was envisaged for monovalent tweezer functionalization, involving the esterification of one tweezer phosphate with a butynyl ester and introduction of an N-terminal azidoglycine into the peptide (Fig. 2). Using computational modeling, we identified K103 at the beginning of Survivin’s C-terminal α-helix as a well-suited anchor for the tweezers with K90/91 as potential alternatives.

### Synthesis of peptide-modified molecular tweezers

The peptide tweezer hybrid molecules became synthetically accessible by a sophisticated general strategy: first, an alkyne ester group was introduced to only one phosphate in the parent tweezer (TW, formerly named CLR01)^37^. This monofunctionalization relies on activation of the free phosphoric acid by trichloroacetonitrile followed by nucleophilic attack of butynol, which occurs only once if the reaction is carried out in pyridine. Second, an *N*-terminal azide group is attached to the peptide with azidoglycine. After cleavage of the peptide from the resin, both components are finally subjected to the standard conditions of a click reaction under Cu-I catalysis (water/THF). No protecting groups are needed on tweezer or peptide, so that the reaction product may be directly purified by preparative HPLC (Fig. 2).

For NES recognition, the peptide ELTL and the elongated peptide ELTLGEFL, which are resembling the natural dimer interface and thus are complementary to the partially overlapping NES of Survivin, were both synthesized by solid phase peptide synthesis (SPPS) with an N-terminal azidoglycine (Azac-peptides, SI1). Subsequently, peptide and monobutynyl tweezer were coupled in a THF/water solvent mixture with ascorbic acid and CuSO_4_ • 5 H_2_O. Both click reactions proceeded smoothly and gave the coupling products in good yields and excellent purity after HPLC purification (SI2). In addition, FAM-labels were introduced into the peptide-modified tweezers via a C-terminal (Fluorescein-labeled lysine)-glycine dipeptide fragment (SI3).

Reaction monitoring was facilitated by the appearance of additional ^1^H NMR signals at 5.2 ppm *(CH*_*2*_*-triazole)* and 8.0 pm *(CH*_*arom*_) indicative of triazole formation. The final peptide tweezers displayed very good water solubility, because they carry multiple negative charges both in the tweezer and in the peptidic part. No self-inclusion of the tweezer moieties was observed in the form of potential upfield shifts in the ^1^H NMR spectra, also ruling out the formation of unproductive tweezer dimers (SI2).

### Characterization of binding by isothermal titration calorimetry

The interaction between the tweezers and Survivin was studied by isothermal titration calorimetry (ITC) and NMR spectroscopy. So far, the only published NMR structure originated from a truncated Survivin (aa 1-120) with improved solubility^38^. Indeed, MD simulations of full-length Survivin revealed a highly flexible C-terminal α-helix fragment around residue 120 (SI4), which might explain poor expression of the full-length protein. Since the truncated construct still contains all relevant parts of the protein and gives excellent NMR spectra (SI5), we chose it for all our in vitro experiments and denote it as Survivin120 in the following results.

**Fig. 3:**
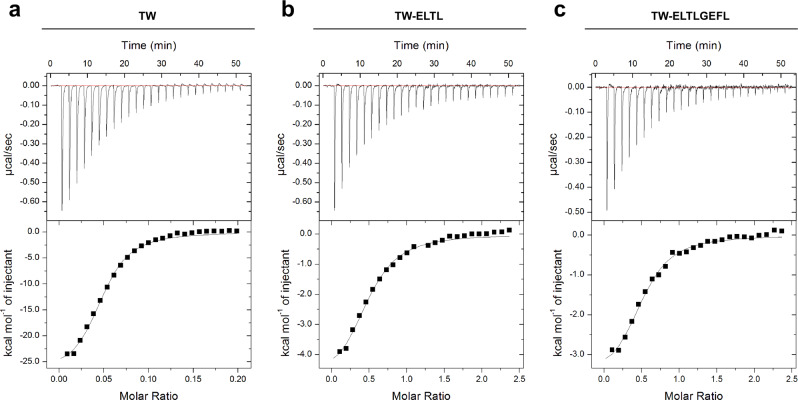
Evidence for tweezer binding to Survivin120 from ITC measurements. Titration of 300 μM TW (**a**) in the cell with 300 µM Survivin120 in the syringe. Titration of 100 µM TW-ELTL (**b**) and TW-ELTLGEFL (**c**) in the cell with 1.2 mM Survivin120 in the syringe. All titrations were performed in PBS, pH 7.4 at 25 °C. The black lines in the bottom panels are the best fit of the data to a one set of sites model. The heat of dilution was subtracted as constant. Dissociation constants were determined to be 38 ± 4 µM for TW, 24 ± 4 µM for TW-ELTL and 19 ± 3 µM for TW-ELTLGEFL. Values reported are the mean ± SEM of the fit. For thermodynamic parameters see SI7. Source data are provided as a Source Data file.

ITC titration of Survivin120 to the tweezers resulted in exothermic binding isotherms (Fig. 3) with dissociation constants K_D_ of 38 µM for the unmodified tweezer TW, 24 µM for TW-ELTL as well as a K_D_ of 19 µM for TW-ELTLGEFL. These values are in good agreement with the unmodified tweezer TW (formerly CLR01) affinity toward lysine (*K*_*D*_ 17 µM)^39^. Fitting the ITC titrations with Survivin120 to a one set of sites model allowed us to derive all relevant thermodynamic data including stoichiometries and changes in Gibbs free energy (G), enthalpy (H), and entropy (S) as summarized in a supplementary table (SI7). Importantly, the unmodified tweezer produced a 20:1 stoichiometry (correlating well with 16 accessible lysine and arginine residues in Survivin120), whereas the modified tweezers displayed 2:1 ligand/protein ratios. This stoichiometry might be plausible with regard to the antiparallel orientation of the Survivin monomers in the respective homodimer, probably allowing the conjugated peptides to also align in both directions with the tweezer either binding to one or the other neighboring anchor lysine. In addition, we carried out reverse titrations by adding increasing amounts of unmodified tweezer TW vs. TW-ELTL into a constant concentration of Survivin (SI8). Interestingly, a sharp kink was produced at a 4–5-fold tweezers excess in both cases, indicating that 4–5 tweezer molecules can be accommodated on the protein surface. However, further addition of unmodified tweezers produced a second substantial exothermic titration step, indicating further unspecific binding to accessible lysine and arginine residues, while the peptide tweezer had already almost reached saturation. Due to the biphasic character these binding curves do not allow the determination of binding constants. However, our data indicate that the introduction of a peptide mimicking Survivin’s natural dimer interface enhanced the tweezer affinity toward Survivin only slightly, but greatly increased its regioselectivity. Although the dimer stability of Survivin120 is not known, it likely represents the binding partner for all tweezers, because apart from the NES region V89-L102 some remote residues P4-W10 contribute additional, mostly aromatic, interactions. Even in the dimer, however, the self-complementary peptide loops (V89-L102) are dynamic and can be expected to expose the NES region temporarily (see discussion of the simulations below).

### Mapping tweezer binding sites by NMR

**Fig. 4:**
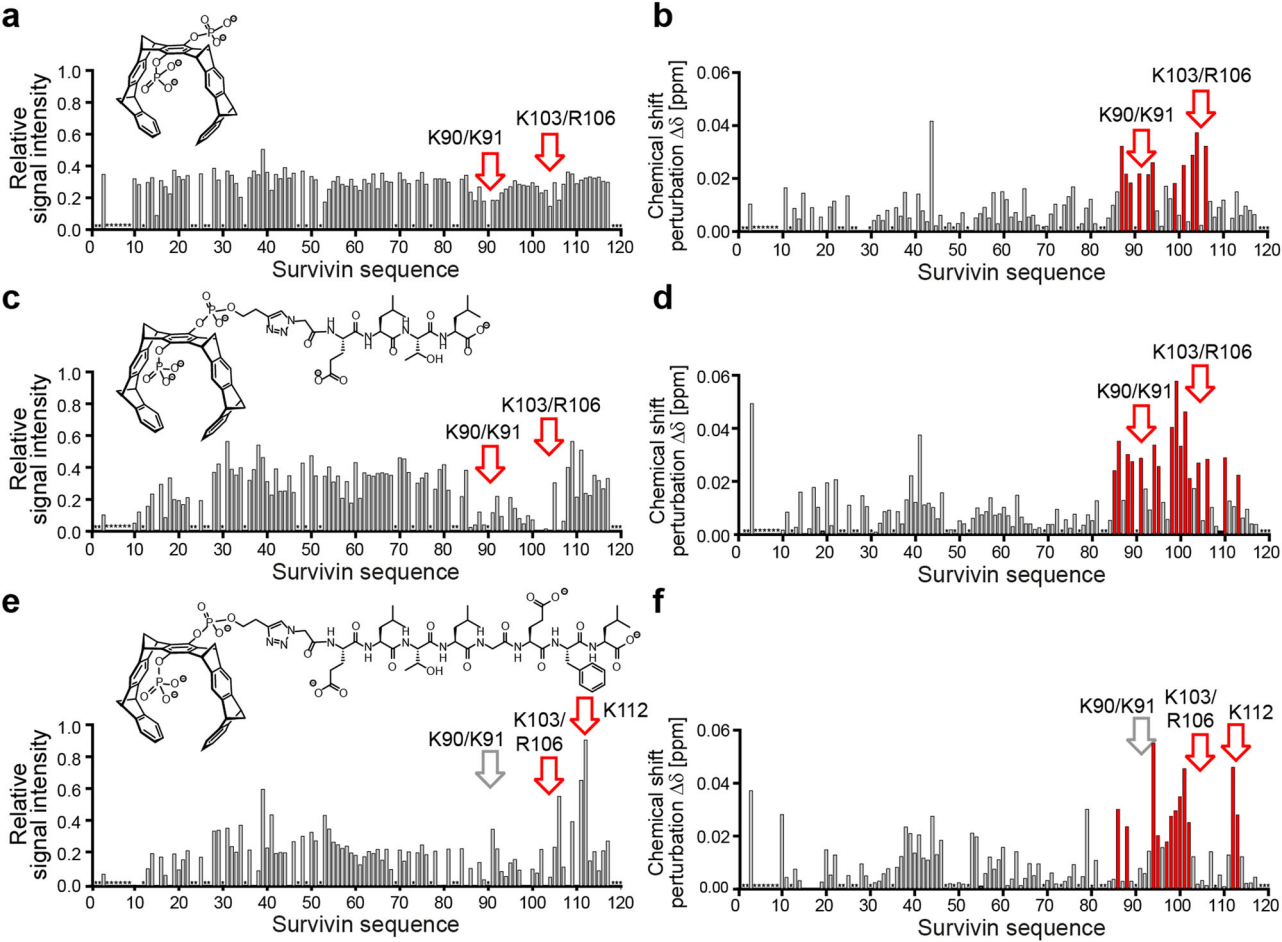
NMR chemical shift perturbation and signal intensity analyses allow us to map binding of molecular tweezers to Survivin120. NMR intensity changes and signal shifts of Survivin120 in complex with one equivalent of tweezers compared to Survivin alone plotted against the amino acid sequence. Normalized signal intensities as well as signal shifts for the unmodified tweezer TW (**a**, **b**), TW-ELTL (**c**, **d**), and TW-ELTLGEFL (**e**, **f**) were identified for each signal and plotted against the Survivin sequence (residues 2-117 as assigned in the BMRB data-base). Residues that were excluded from analysis because they are not visible in the spectra or suffer from signal overlap are marked with an asterisk. Residues with a prominent shift or reduced signal intensity (red) are clustered around Survivin’s NES, and lysine and arginine residues are additionally marked with an arrow. Upon titration with TW-ELTL and TW-ELTLGEFL, signal intensities collapse around the NES region. Source data are provided as a Source Data file.

To map tweezer binding to distinct amino acid residues of Survivin, we performed NMR titrations adding tweezers up to equimolar amounts to ^15^N-labeled Survivin120 (SI5). Binding of a ligand causes signal shifts and often reduced signal intensities for the residues involved in the interaction (Fig. 4). Titration of the unmodified tweezer resulted in reduced signal intensities and shifts around the basic amino acids K91, K103, and R106 (Fig. 4a, b). K90 also lies in the regions identified; however, it is not assigned in the spectrum. In addition, signal intensities decrease in the same regions (Fig. 4d) confirming these as potential tweezer binding sites. Unfortunately, the applied NMR method does not allow us to differentiate between tweezer binding to residues K90 vs. K91 and K103 vs. R106 since they are in too close proximity. Titration of TW-ELTL (Fig. 4c, d) as well as TW-ELTLGEFL (Fig. 4e, f) decreased signal intensities in this region even more and enhanced signal shifts for the same basic residues. In addition, the NES residues between K91 and K103 experienced an intensity loss and significant signal shift when the peptide-linked tweezers were binding in contrast to the unmodified tweezer. This shows that the peptides indeed contact the NES region and confirms the desired regioselectivity of the tweezer conjugate on Survivin’s surface. The overall line shape of the HSQC spectra indicates that Survivin120 likely remains dimeric upon tweezer binding. If the dimer would dissociate upon ligand binding, a sharpening of the signals and thus an intensity increase would occur due to slower T2 relaxation. Rather the contrary is observed, especially at ligand:protein ratios >1:1, which indicates beginning aggregation. Nevertheless, anchoring the peptide–tweezers nearby the NES with direct peptide–NES interactions is expected to shield the natural binding site and significantly weaken the Survivin–CRM1 interaction.

**Fig. 5:**
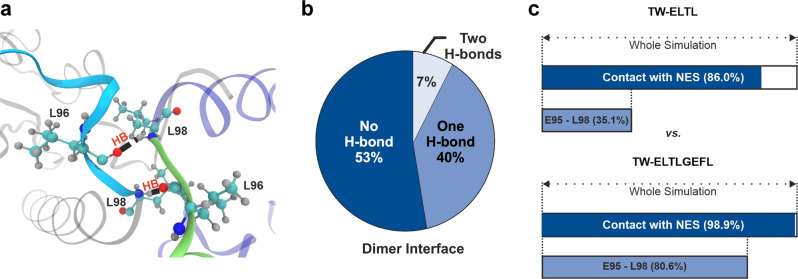
GaMD simulations and QM/MM calculations on the tweezers–Survivin interaction provide deeper insights into the binding event. **a** Main hydrogen bonds (HB, red) established between Survivin monomers (gray, violet) at the ELTL interface (contact region of both monomers overlapping with the NES, represented in cyan and green). The leucine residues engaged in these interactions are shown in CPK representation. **b** Occurrence of hydrogen bonds at the dimer interface. **c** Frequency of noncovalent contacts between the tweezers, bound to K103, and the NES as well as with the ELTL region (^95^ELTL^98^) of the dimer interface.

For a better understanding of the complexation process, we performed MD and Gaussian accelerated molecular dynamics (GaMD) simulations as well as quantum mechanics/molecular mechanics (QM/MM) calculations (see SI10 for computational details). TW–lysine interactions were calculated in the Survivin120 monomer (protomer A, structure with PDB-ID 1XOX [https://www.rcsb.org/structure/1xox])^38^. The monomer, unlike the dimer structure, displays an exposed NES region, which is a key feature for the activity of Survivin. Thus, it represents the most suitable model to study the interactions of the tweezers with the lysine residues in this region. Four well-accessible lysine residues (K23, K90, K91, and K103) were selected on the monomeric protein for an in-depth characterization of their binding mode with TW as well as TW-ELTL. MD and GaMD simulations evidenced interactions of the peptide-modified tweezers with Survivin120 via the peptide moieties (SI12, Table 1, and Fig. 5). Due to the lack of the peptide motif, such interactions cannot be established by the bishydrogenphosphate-substituted tweezer TW. Since the inclusion complexes of lysine with TW and TW-ELTL otherwise display remarkably similar structural features (SI13), we can assume that the additional interactions found in the peptide-modified tweezers contribute to their improved selectivity toward K103/K91. We quote these interactions as dynamic because of the lack of a highly conserved binding pose of the peptide fragment.

**Table 1:** The relative energies of the QM region indicate that the tweezer–lysine complexes are most stable in positions 103 and 91.

TW-ELTL	Relative energy QM region (kcal/mol)
K103	0 ± 1
K91	1 ± 14
K90	27 ± 6
K23	68 ± 3

Interestingly, a conserved interaction between the peptide tail of TW-ELTL and the homologous segment in the protein (^95^ELTL^98^) occurred only when TW-ELTL was encapsulating K103 (SI12). This particular site allows for the antiparallel pairing found in the naturally occurring dimer structure. According to the QM/MM calculations, this site also produced a very stable complex (Table 1 and SI13), which is further stabilized by a salt bridge with R106 (SI14). Therefore, we also performed GaMD simulations of the monomer of Survivin120 and a modified tweezer with the elongated peptide, TW-ELTLGEFL, on K103. At this point, it is important to notice that the above-discussed structural details are in very good agreement with the shift and intensity changes observed by NMR experiments, with one exception: they require an exposed NES region such as in monomeric Survivin. This apparent discrepancy can be rationalized by the conformational flexibility at the weakly associated dimeric NES region. We therefore also performed GaMD simulations of the Survivin120 dimer in an explicit solvent box. The frequency of hydrogen bond formation during the simulation within the dimer interface was analyzed (Fig. 5a, b). The results show that the strongest interaction at the ^95^ELTL^98^-region of the dimer interface (involving two hydrogen bonds) has a very low prevalence along the simulation. In the most frequent scenario, no hydrogen bonds are formed, indicating weak and labile interactions between the interface fragments ^95^ELTL^98^ in both monomers. Not surprisingly, the GaMD simulations also showed that TW-ELTLGEFL is able to form more noncovalent interactions with the NES region than the shorter TW-ELTL. This difference becomes most apparent in the ELTL region of the interface (^95^ELTL^98^) (Fig. 5c). The peptide substituents of the modified tweezers are rather flexible, allowing for frequent interactions with the NES. It seems that the rigid triazole linker acts as an anchor point and facilitates these interactions (SI15)—a synergistic effect between the peptide motif and the (otherwise inactive) linker fragment. As expected, the longer, more flexible peptide chain of TW-ELTLGEFL explores a larger conformational space and establishes more attractive interactions with the NES, explaining its superior performance in binding experiments. Our calculations show weak and dynamic interactions at the dimer interface. Such features might be leveraged by the TW-anchored peptide to form contacts with one of the Survivin protomers and, hence, shield the corresponding NES region. Without further structural information we cannot exclude that the peptide might also bind in a way that stabilizes the dimer interface and thus prevents CRM1 from binding a Survivin monomer.

### Tweezer interference with the export complex assembly in vitro

**Fig. 6:**
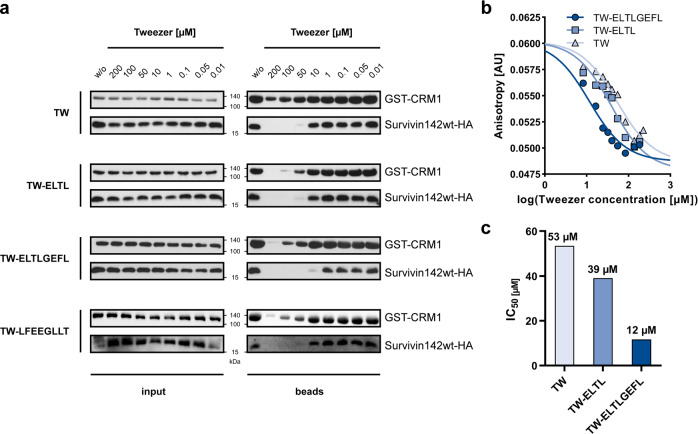
The assembly of the export complex is disturbed by unmodified and peptide-linked tweezer molecules. **a** 293T cell lysate with overexpressed Survivin142-HA was preincubated with either unmodified tweezer (TW), TW-ELTL, TW-ELTLGEFL, or a scrambled peptide-modified tweezer, TW-LFEEGLLT, at concentrations ranging from 0.01 to 200 µM. GST-CRM1 was mixed with either non- or preincubated cell lysates in the presence of recombinant RanQ69L and dGTP to allow complex assembly. GST-CRM1 and interacting proteins were pulled by GSH-Sepharose beads. Proteins in input and beads samples were analyzed via immunoblotting with antibodies specific for GST or HA. For each tweezer, samples derive from the same experiment and gels/blots were processed in parallel. Direct comparison for this exact concentration range was performed once for TW, TW-ELTL, and TW-ELTLGEFL and for TW-LFEEGLLT in three technical replicates. **b**, **c** Atto488-labeled Survivin120 was preincubated with CRM1_1-1062VLV430AAA mutant in a ratio of 1:5 and titrated with supramolecular tweezers up to approx. 180 µM. Fluorescence anisotropy was measured (*n* = 1) (**b**), and IC50 values were determined from the resulting curves (**c**). TW light blue/ triangles, TW-ELTL blue/squares, TW-ELTLGEFL dark blue/circles. Source data are provided as a Source Data file.

We next investigated whether tweezers inhibit the Survivin–CRM1 complex formation in vitro. The effects of the unmodified and peptide-linked tweezers were analyzed via pull-down experiments with 293T lysates containing overexpressed HA-tagged Survivin142, recombinant GST-CRM1 as bait protein, and tweezers (Fig. 6). The parent and ELTL-linked tweezer required 10–50 µM to disrupt the Survivin–CRM1 complex. TW-ELTLGEFL was already effective at 1–10 µM and thus turned out to be more potent. In order to provide experimental evidence for the sequence selectivity of the TW-ELTLGEFL ligand, a scrambled analog was synthesized and clicked to the tweezer, resulting in the hybrid molecule TW-LFEEGLLT (SI1, SI2, and SI10). Intriguingly, this ligand was about one order of magnitude less effective than the one bearing the original NES-derived sequence and rather comparable to the unconjugated tweezer TW with regard to the pull-down analyses (Fig. 6a and SI18). Moreover, ITC titrations with TW-LFEEGLLT revealed much lower heat changes, and the corresponding *K*_*D*_ value dropped from 19 ± 3 for TW-ELTLGEFL to 68 ± 22 μM for the scrambled peptide conjugate (SI16 and S17). Thus, it displays significantly (~three times) lower affinity toward the wildtype protein, indicating that the correct self-complementary NES sequence is indeed essential for efficient ditopic recognition. Additional NMR studies demonstrated that the tweezer with the scrambled peptide sequence is still able to bind to the two sites K90/K91 and K103/R106 like the unmodified TW (SI17). However, no large perturbations as for TW-ELTL or TW-ELTLGEFL are observed in between residues 91–103, indicating that the peptide moiety does not form specific contacts with Survivin. Instead, the slight chemical shift perturbations might be due to the spatial proximity of the peptide moiety to the anchoring residue. All those results strongly indicate that the additional binding energy from the NES interface was lost due to scrambling and thus point toward specific recognition as the reason for increased selectivity and interference with the Survivin–CRM1 interaction. Of note, very high tweezer concentrations (100–200 µM) also weakened GST-CRM1 binding to GSH-Sepharose beads, most likely by low-affinity binding to the GST protein. However, direct ITC titrations gave only small heat changes and revealed that this interaction is endothermic (SI9).

We also used fluorescence anisotropy experiments to investigate the ability of the tweezer molecules to disrupt the Survivin–CRM1 complex. Atto488-labeled Survivin120 was preincubated with the CRM1_1-1062VLV430AAA mutant^40^ that binds Survivin irrespective of RanGTP and titrated with tweezers. The presence of all tweezers significantly lowered the fluorescence anisotropy, indicating a potent disruption of the Survivin–CRM1 complex (Fig. 6b, c). IC_50_ values were determined at 53 µM for the unmodified tweezer, 39 µM for TW-ELTL, and 12 µM for TW-ELTLGEFL. Thus, pull-down and fluorescence anisotropy experiments both indicated that the peptide modification increases the inhibitory potential of the tweezers for the Survivin–CRM1 interaction.

### Confirmation of the tweezer binding site

**Fig. 7:**
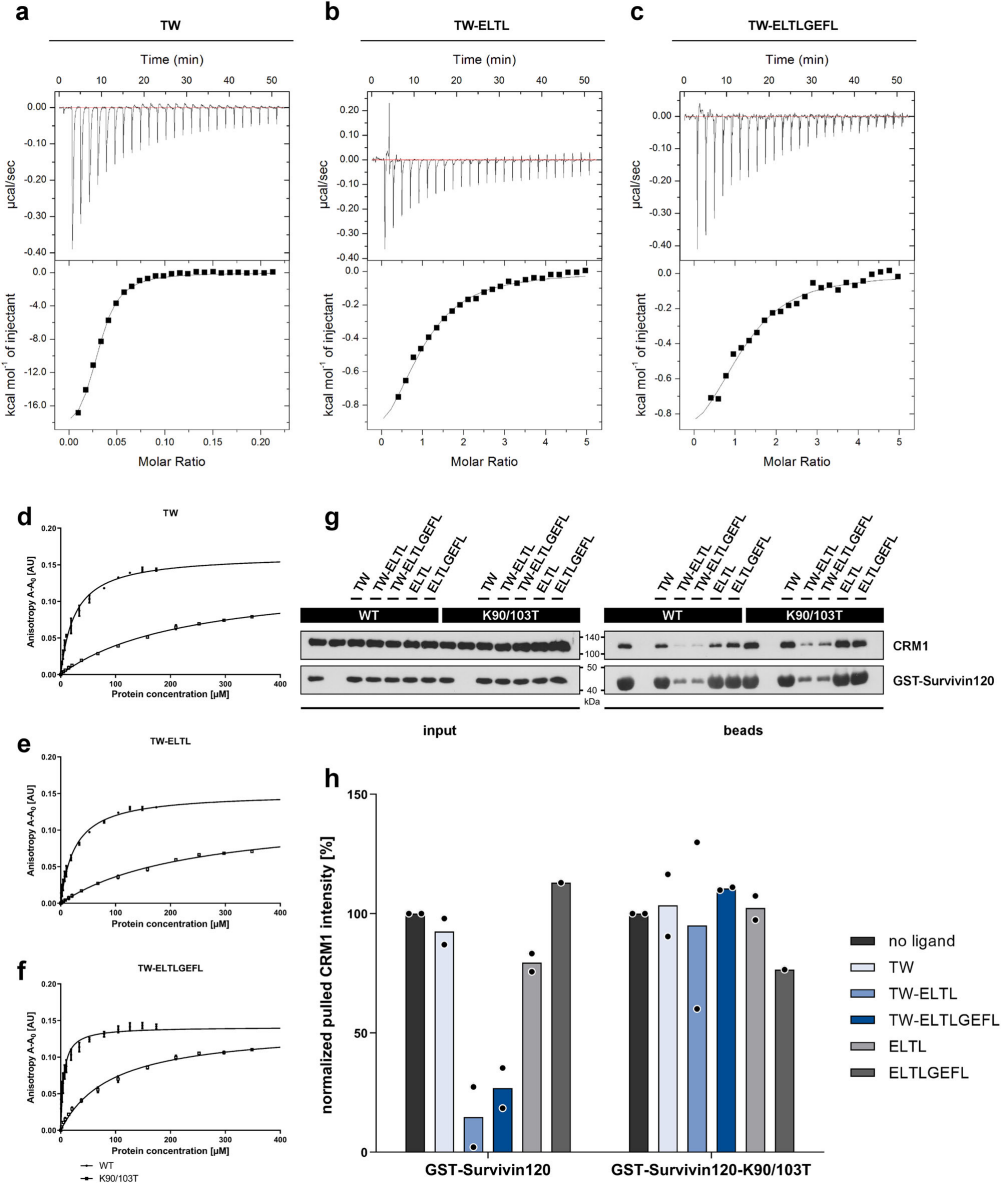
Lysine to threonine mutations near Survivin’s NES and dimer interface reduce tweezer affinity and impair its inhibitory effect on the Survivin–CRM1 interaction. Titration of 300 μM TW (**a**) in the cell with 321 µM Survivin120 K90/103T in the syringe. Titration of 100 µM TW-ELTL (**b**) and TW-ELTLGEFL (**c**) in the cell with 2.5 mM Survivin120 K90/103T in the syringe. All titrations were performed in PBS, pH 7.4 at 25 °C. Graphs represent one representative example each from three independent experiments (*n* = 3). The black lines in the bottom panels are the best fit of the data to a one set of sites model. The heat of dilution was subtracted as constant. For thermodynamic data derived from the graphs see SI7. FAM-labeled unmodified tweezer molecule (**d**), TW-ELTL (**e**), and TW-ELTLGEFL (**f**) (0.2 µM) were titrated with either Survivin120 (200 µM, circles) or Survivin120 K90/103T (400 µM, squares). Survivin120 K90/103T showed greatly reduced tweezer affinities (lower curves). **d**–**f** Data are presented as mean values ± SD with *n* = 3 independent experiments. **g** Pull-down results after immunostaining. GST-Survivin120-WT or GST-Survivin120-K90/103T were incubated with 50 µM respective tweezer molecule or ELTL/ ELTLGEFL peptides w/o tweezer. GST-Survivin120- or GST-Survivin120-K90/103T-loaded beads were mixed with CRM1 and RanQ69L prey proteins as well as dGTP to allow export complex assembly. Proteins in input and bead samples were analyzed via immunoblotting with antibodies specific for CRM1 or GST. WT, wildtype. One representative example of two independent biological replicates is shown. Samples derive from the same experiment and gels/blots were processed in parallel. **h** Quantification of two independent pull-down experiments. After subtraction of the CRM1 negative control from the pulled CRM1 intensity, the latter is normalized by the GST–Survivin intensity and afterwards normalized by the CRM1 intensity without tweezer incubation. Export complex assembly is only compromised by peptide tweezers in the wildtype Survivin120, but not in the mutant. No ligand: black; TW: light blue; TW-ELTL: blue; TW-ELTLGEFL: dark blue; ELTL peptide: light gray; ELTLGEFL peptide: dark gray. Source data are provided as a Source Data file.

If the tweezer-based inhibitors indeed bind to lysines/arginines flanking the NES (K90, K91, K103, R106), their mutation to, e.g., threonine, should abolish the observed effects. For this reason, we generated double and triple Survivin120 mutants lacking these putative tweezer anchor points. Unfortunately, most double mutants and especially the triple mutants were unfolded as evidenced by 1D ^1^H NMR (SI6); finally, the correctly folded double threonine mutant Survivin120 K90/103T was chosen for further investigation. Indeed, ITC titrations with this mutant revealed lower tweezer affinities (dissociation constants *K*_*D*_ of 49 ± 5 µM for the unmodified tweezer TW, 50 ± 10 µM for TW-ELTL, and 36 ± 10 µM for TW-ELTLGEFL) and 1:1 stoichiometries for the peptide-modified tweezers (Fig. 7a–c and SI7). Next, binding of FAM-labeled tweezers (SI3) to either Survivin120 wildtype or the K90/103T mutant was studied by fluorescence anisotropy (Fig. 7d–f). For Survivin120 wildtype, the affinities correspond well to those values obtained from the initial ITC titrations: TW-FAM *K*_*D*_ of 27 ± 2 µM, TW-ELTL-FAM *K*_*D*_ of 26 ± 2 µM, and TW-ELTLGEFL-FAM *K*_*D*_ of 5 ± 0.5 µM (potential stabilizing influence of the FAM unit). Binding to the Survivin120 K90/103T mutant, however, was strongly impaired (*K*_*D*_ values of 240 ± 20, 240 ± 20 and, 92 ± 5 µM). We conclude that lysines 90 and 103 are indeed essential for efficient tweezer binding.

Next, we investigated whether tweezer inhibition of the export complex assembly is compromised upon lysine mutations near the NES. Therefore, pull-down experiments were performed with recombinant GST-tagged Survivin120 wildtype and K90/103T as bait proteins, tweezers, recombinant CRM1 and RanQ69L (Fig. 7g). Controls lacking the Survivin baits were included, as well as isolated peptides without tweezer (Fig. 7h). In these experiments, both tweezer peptide conjugates potently inhibited the interaction between CRM1 and GST-Survivin120; in sharp contrast, the parent tweezer TW and uncoupled peptides were all inactive. However, for the double lysine mutant GST-Survivin120-K90/103T even the tweezer conjugates lost most of their inhibitory power toward the essential Survivin–CRM1 interaction. This is an important control: it indicates that the tweezer conjugates address specific lysine residues in Survivin’s NES and thus shield it against CRM1.

## Discussion

Inhibition of the essential Survivin–CRM1 interaction is of great interest because it regulates cell proliferation and mediates a cytoprotective function^27,30^. However, the development of CRM1 binders bears the disadvantage that it affects a large number of cargo proteins and is therefore not specific for Survivin. Here, we present prototypes for an alternative strategy: to address Survivin’s NES with specific supramolecular tweezer conjugates, which dock onto the overlapping natural dimer interface on Survivin’s protein surface with low micromolar affinities. This alternative is now accessible by click reactions from an alkyne-modified parent tweezer. We emphasize that this synthetic strategy greatly expands the design of modified tweezers, because it can be applied to tweezers with one or two phosphate arms and is not restricted to peptides. Virtually any additional functional unit can now be attached to the tweezers by click chemistry: fluorescence labels, chemically reactive groups, peptidic and other recognition units, Au nanoparticles (via C-terminal cysteines), and scaffolds with various alkynes for multivalency have already been introduced^37,41^. This functionalization synthetic strategy now opens up a pioneering class of advanced tweezer derivatives with two or more functions.

Moreover, as a proof-of-principle, our experiments confirm that binding of peptide-equipped tweezers occurs in Survivin’s NES region overlapping with the dimer interface and therefore impairs the interaction with CRM1. To the best of our knowledge, this is the so far sole successful example that an amino acid binder (lysine tweezer) is directed to a specific epitope of a protein—in our case by conjugation to the self-complementary dimer interface comprising the NES sequence. The underlying rational design was supported by ITC titrations and NMR spectroscopy that produced maximum chemical shift perturbations on four lysine/arginine residues flanking the NES. Detailed MD and GaMD simulations complemented with QM/MM calculations revealed K103 as a preferred binding site and supported that even in the dynamic dimer the NES signal is partially exposed to approaching ligands. Pull-down experiments and fluorescence anisotropy titrations both indicated that the peptide modification indeed increases the inhibitory potential and specificity of the tweezers for the Survivin–CRM1 interaction. Importantly, a tweezer molecule equipped with a scrambled peptide motif was significantly less effective with regard to binding and inhibition of the relevant Survivin–CRM1 interaction. Again, this points toward a specific recognition as the origin for increased selectivity of our hybrid ligand. Finally, a double lysine mutant of Survivin (K90/103T) further substantiated the identified binding sites, because here the potent tweezer conjugates lost most of their inhibitory potential (ITC, pull-down, FT). Our sophisticated synthetic approach allows the formation of labeled tweezer peptide conjugates for advanced binding experiments, which may also find applications in fluorescence imaging.

Even though the peptide modification increased the ability of the tweezers to shield the NES, it had only moderate impact on the binding affinity. Obviously, additional binding energy must be generated by tailored recognition units incorporated into the tweezer conjugates. A second tweezer unit at the opposed end of the peptide may serve this purpose, or alternatively more powerful supramolecular NES binders of synthetic or natural origin.

In this study, we established that a supramolecular amino acid binder can be designed for an exposed surface epitope on a given protein target. Our strategy involves the combination of a lysine–selective tweezer with a peptidic recognition element for the desired binding epitope. This was accomplished by trichloroacetonitrile-assisted monoesterification of a single tweezer phosphate with butynol and subsequent click reaction with an azide-modified peptide without the need of any protecting group. Attachment of a single peptide arm renders the hybrid tweezer selective for the peptide loop representing the NES in Survivin which is self-complementary and flanked by well-accessible lysine residues. The design was guided by MD and GaMD simulations as well as QM/MM calculations of the putative tweezer–protein complexes. Structural evidence was provided by 2D NMR spectroscopy, affinities were determined by ITC titrations. The hybrid tweezers were able to disrupt the essential complex between Survivin and its export receptor CRM1 in cell lysates as demonstrated with pull-down assays and in vitro as shown by fluorescence anisotropy measurements. Labeled tweezer hybrids revealed strongly diminished affinities to a Survivin double mutant that lacked the NES-flanking lysines and confirmed the selectivity for the respective epitope on Survivin. We thus accomplished the proof-of-principle of epitope targeting by supramolecular binders. Further optimization should improve the performance of our ligands by, e.g., by replacing the peptide unit resembling the natural dimer interface of Survivin with much more powerful interaction partners from the CPC, such as Borealin fragments. Alternatively, we plan to employ dimeric tweezers with an internal peptide unit—in order to exploit two attachment sites to the NES region. Lysine 90 and 103 have been proven very well suited for this purpose and work into this direction is underway in our laboratory.

In the future, supramolecular inhibition of the CRM1–Survivin interaction should be transferred into the cellular context in order to further probe Survivin’s biological functions and to gain control over its export activity. The introduction of fluorescence labels into the next generation of tailored tweezer conjugates via click chemistry will facilitate their detailed monitoring by confocal microscopy. Our study is an experimental proof-of-principle that specific shielding of intracellular transport signals can indeed be accomplished by supramolecular ligands. In the past, inhibition of nucleo-cytoplasmic transport, including nuclear export and import processes, was only achieved in a rather unspecific manner targeting the respective receptors instead of the bound signals, exemplified by the drug Leptomycin B binding to Crm1. As the activity of several disease-driving proteins besides Survivin is based on selective nuclear transport and protein interactions, our results could indeed set the stage for a broad future exploitation of the developed principles in basic and applied biomedical research.

## Methods

### Synthesis of tweezer conjugates

Due to its excellent biocompatibility and very good tolerance toward peptidic side chains^42^ the copper-catalyzed Huisgen cycloaddition was employed to couple an alkyne tweezer with an azidopeptide^43^. To this end the unsymmetrical monophosphate monobutynylphosphate tweezer was synthetized according to our recently published protocol for the synthesis of unsymmetrical diphosphate monoesters via the trichloroacetonitrile method^37^. All peptides were prepared with a final coupling of azidoacetic acid to their *N*-terminus. Cleavage from the resin and purification by preparative HPLC yielded pure peptides. Subsequent click reactions between free azido peptides and alkyne tweezers were carried out in a mixture of water and THF (1:1). The catalyst was prepared in situ by reaction of copper sulfate with sodium ascorbate in the presence of DIPEA base. The resulting hybrid molecules were precipitated by acidification with HCl, followed by removal of THF in vacuo and filtration. Unreacted starting materials could be separated from the products by RP-18 column chromatography or preparative HPLC.

### Peptide synthesis

All peptides were synthetized using automated, microwave assisted, SPPS. The synthesis was carried out on a CEM peptide synthesizer using a Wang resin (4-hydroxybenzyl alcohol (PHB) on polystyrene) already equipped with the C-terminal amino acid. Coupling was effected with HCTU. In the final step, 2-azidoacetic acid was coupled to the free *N*-terminus of the peptide; then the entire peptide was cleaved off the resin with TFA, TIS, and water. The peptide in the cleavage solution was poured onto ice-cooled diethyl ether and stored in the freezer for 1 h to precipitate. Each peptide was pre-purified by centrifugation and washed again with diethyl ether. Subsequently, preparative purification was carried out by means of HPLC. Purification was performed on a preparative HPLC system from Jasco with UV/Vis detector (UV-975, DG-2080-53 solvent degasser, LG-980-02S 3-channel solvent mixer, peak-detection at 210  nm). The instrument is equipped with a reverse-phase column from Macherey-Nagel (Modell EC 250/4 Nucleosil 100-3 C18). Linear gradients of acetonitrile and water with presence of 0.1 TFA were applied.

### Click coupling

Monophosphate monobutynylphosphate tweezer (5.0 mg, 6.4 µmol) was dissolved in 2 mL THF/H_2_O (1:1) in a 5-mL round-bottom flask together with the respective *N*-terminal Azac peptide (23 µmol). Fresh distilled DIPEA (11.3 µL) was added to the previously degassed solution. Subsequently, the copper sulfate solution (8.3 mg CuSO_4·_5H_2_O, 33 µmol in 1 mL water) was mixed with the sodium ascorbate solution (13 mg C_6_H_7_NaO_6_, 66 µmol in 1 mL water) and the catalytic brew was immediately added to the reaction solution. The reaction mixture was stirred for 16 h at room temperature and subsequently quenched by addition of 1 M HCl (5 mL), resulting in formation of a colorless (yellow in the case of FAM-labeled derivates) precipitate, followed by removal of THF in vacuo. The suspension was extracted with chloroform (3 × 5 L). The aqueous phase was filtered and the collected solid was washed with water (2 × 1 mL). The crude product was rinsed with distilled THF from the fritted funnel and the desired TW-peptide conjugate was obtained as a colorless (or yellow) solid after evaporation to dryness (6 µmol, 94 %). LC traces of all final products (peptides, FAM-labeled peptides, tweezer molecules) can be found in the supplementary information (SI19).

### Plasmids

Bacterial expression vectors encoding Survivin120 variants, CRM1, and RanQ69L were constructed by polymerase chain reaction (PCR) amplification using appropriate templates and primers containing ApaI/BamHI restriction sites (SI20). PCR products were cloned into the vector pET41-GST-PreSc as an *N*-terminal fusion with GST and a PreScisson protease cleavage site as described (SI20)^26^. To generate Survivin point mutants, critical lysines were changed by site-directed mutagenesis with the Q5^®^ Site-Directed Mutagenesis Kit from New England BioLabs^®^. The eukaryotic expression vector pc3-Survivin142-HA was analogously constructed by PCR amplification using an appropriate template and primers containing BamHI/NheI restriction sites (SI20). The PCR product was cloned into the vector pcDNA3.1 as a *C*-terminal fusion with an HA expression tag and transfected as described^26^.

### Protein expression and purification

GST-tagged proteins were expressed in Escherichia coli soluBL21 cultivated in LB media containing 50 µg/mL kanamycin. The expression was induced with 1 mM IPTG at an OD_600_ of 0.6–0.8. Bacteria were pelleted, lysed with lysozyme, and subsequent sonication in TRIS/NaCl (pH 7.4) supplemented with 1 mM PMSF. The GST-tagged proteins were then immobilized on GSTrap 4B columns. The GST-Tag was optionally cleaved with PreScission protease on column, depending on the experiments performed afterwards. The protein was then loaded on a HiTrap Q HP column and eluted with a 0.025–1 M NaCl gradient in 50 mM Tris-HCl buffer, pH 7.5, containing 1 mM DTT.

His-tagged CRM1_1-1062VLV430AAA_ was expressed in *E.* *coli* BL21-CodonPlus-RIL from Agilent Technologies using a pTGA20 vector obtained from Dr. Sonia Banuelos (Department of Biochemistry and Molecular Biology, Biofisika Institute, University of the Basque Country, Leioa, Spain). Bacteria were cultivated in LB media containing 100 µg/mL carbenicillin for the pTGA20 vector and 50 µg/mL chloramphenicol for maintaining the pACYC plasmid in the BL21-Codon Plus strain. The expression was induced with 0.1 mM IPTG at an OD_600_ of 0.5. Bacteria were pelleted and lysed with lysozyme and subsequent sonication in TRIS/NaCl (pH 7.4) supplemented with 1 mM PMSF. The lysates were then immobilized on HisTrap FF columns and eluted using 50 mM NaH_2_PO_4_, 300 mM NaCl, and 500 mM imidazole, pH 8.0. Afterwards the His-Tag was cleaved with TEV protease from Sigma-Aldrich overnight at 4 °C. The protein was dialyzed against 50 mM Tris-HCl and 25 mM NaCl, pH 7.5 and passed over another HisTrap FF column to remove the cleaved His-Tag. The protein was then loaded on a HiTrap Q HP column and eluted with a 0.025–1 M NaCl gradient in 50 mM Tris-HCl buffer, pH 7.5, containing 1 mM DTT.

Isotopically ^15^N-labeled GST-tagged Survivin120 was expressed in *E. coli* SoluBL21 by growing a culture in 4 L LB medium at 37 °C. At an OD_600_ of 1.0–1.2, the bacteria were pelleted and resuspended in 1 L M9 minimal medium supplemented with 1 g/L ^15^N-ammonium chloride. After incubation for 30 min at 37 °C, expression was induced with 0.2 mM IPTG and the protein was expressed for 20 h at 30 °C. The cells were harvested and lysed by sonication in PBS (pH 7.4) supplemented with 1 mM PMSF. ^15^N-GST-Survivin120 was purified via a GSTrap 4B affinity column. The GST-tag was cleaved with PreScission protease for 6 h at 4 °C. Subsequent preparative size exclusion chromatography was performed with a HiLoad 26/600 Superdex 75 pg column and a downstream mounted GSH-column from GE Healthcare in 50 mM KPi pH 7.4 with 150 mM KCl and 2 mM DTT. The pure protein was concentrated, and the buffer was exchanged to NMR buffer (50 mM KPi pH 6.5, 90 mM KCl, 2 mM DTT) using Vivaspin Ultracentrifugation filters with a molecular weight cutoff of 10 kDa.

### Isothermal titration calorimetry (ITC)

ITC was performed with a MicroCal iTC200 from Malvern Panalytical in PBS, pH 7.4 at 25 °C with molecular tweezers in the cell and Survivin120 in the titration syringe. The protein was dialyzed overnight at 4 °C against the PBS buffer. Ligands were dissolved in the respective dialysis buffer. Then, 300 µM TW were titrated with 300 µM Survivin120 WT or 321 µM Survivin120 K90/103T. Then, 100 µM TW-ELTL, TW-ELTLGEFL, or TW-LFEEGLLT was titrated with 1.2 mM Survivin120 WT or 2.5 mM Survivin120 K90/103T. For reverse titrations, either 33.3 or 34.4 µM Survivin120 in the cell was titrated with 5 mM TW or TW-ELTL in the syringe, respectively. All titrations were performed in PBS, pH 7.4 at 25 °C. Then, 1.5 μL injections were used with 120 s spacing time between injections. The injection rate was set to 0.5 µL/s and the reference power was 5 µcal/s. ITC thermograms were fitted to a one set of sites model with the software Origin (v7.0552) provided with the instrument. Heat of dilution was subtracted as constant from each data point.

### Pull-down experiments

All pull-down assays were performed at room temperature in pull-down buffer (PBS, pH 7.4, 1 mM DTT, 0.02 Triton X-100). GST fusion proteins were immobilized on 50 μL GSH-coated Sepharose 4B beads from GE Healthcare. The beads were prior equilibrated and blocked with 500 μL pull-down blocking buffer (PBS, pH 7.4, 1 (w/v) BSA, 1 mM DTT, 0.02 (v/v) Triton X-100) for 1 h to prevent unspecific binding.

For analysis of the effective inhibitory tweezer concentration, 200 μg 293T lysate with overexpressed Survivin142-HA was preincubated without ligand or with different concentrations (10 nM to 200 μM) of unmodified tweezer TW, TW-ELTL, TW-ELTLGEFL, or TW-LFEEGLLT for 1 h and then mixed with 35 μg GST-CRM1, 55 μg RanQ69L, and 2 mM dGTP. GSH-beads were incubated with this protein mixture for 2 h under rotation. For analysis of tweezer specificity, 40 μg GST-Survivin120 or GST-Survivin120-K90/103T point mutant was pre-bound to equilibrated GSH-beads in 500 μL pull-down buffer, containing additionally either no ligand or 50 μM unmodified tweezer TW, TW-ELTL, TW-ELTLGEFL, or peptides ELTL and ELTLGEFL, for 1 h under rotation. After washing and blocking, GSH-beads were incubated with a protein mixture consisting of 2 mM dGTP, 50 μg CRM1, and 50 μg RanQ69L for 2 h under rotation.

Samples of input and beads taken during pull-down experiments were ran on 12.5 SDS gels and transferred onto 0.2 µM PVDF membranes (Amersham Hybond P 0.2) using a PerfectBlue™ tank electro blotter (Peqlab) at 350 mA for 150 min. Membranes were blocked for 1 h using 5% milk powder in TBS buffer containing 0.1% Tween-20. Next, membranes were incubated in primary antibodies against HA (anti-HA, mouse monoclonal, BioLegend, Covance MMS-101R, 1:1,000), GST (anti-GST, mouse monoclonal, Santa Cruz Biotechnology Inc., sc-57753, 1:1000) or CRM1 (anti-CRM1, rabbit polyclonal, Novus Biologicals Ltd., NB100-79802, 1:10,000) overnight at 4 °C. Secondary horseradish peroxidase (HRP)-conjugated antibody (anti-mouse IgG-HRP, sheep, GE Healthcare, NXA931 or anti-rabbit IgG-HRP, donkey, GE Healthcare, NA934) was then added (1:10,000) for 1 h at room temperature. Chemiluminescence was detected and imaged using the Pierce ECL Plus Western Blotting Substrate kit (Thermo Fisher Scientific) and Chemidoc system (Bio-Rad) or film processor Cawomat 2000 IR (CAWO). Western blots were analyzed by densitometric analysis with ImageJ version 1.52p (U.S. National Institutes of Health), measuring the mean gray intensity of protein bands. Uncropped and unprocessed scans of western blots are deposited in the Source Data file.

### NMR experiments

NMR experiments were performed on a Bruker 700 MHz Avance Ultrashield NMR spectrometer (Bruker, Germany) equipped with a 5 mm CPTCI ^1^H-^13^C/^15^N/D cryoprobe with z-gradient at 25 °C. The pulse program for the ^1^H-^15^N-BEST-TROSY-HSQC is part of the NMRlib 2.0 pulse sequence tools library from IBS (Grenoble, France) available at http://www.ibs.fr/research/scientific-output/software/pulse-sequence-tools/. Spectra were processed with Topspin 3.5 and analyzed in CARA^44^. Histograms plotting chemical shift perturbation or signal intensities against the protein sequence were generated in GraphPad Prism 5. The assignments for the Survivin120 construct were obtained from BMRB entry # 6342.

Protein NMR samples for ^15^N-HSQC titrations contained 400–600 µM ^15^N-labeled Survivin120 in NMR buffer (50 mM KPi pH 6.5, 90 mM KCl, 2 mM DTT with 10% D_2_O). A 10 mM (TW) or 5 mM (TW-ELTL, TW-ELTLGEFL, TW-LFEEGLLT) stock solution of ligand in water was added stepwise to the protein samples. Tweezers were titrated stepwise up to a 1:1 ratio, and ^1^H-^15^N-BEST-TROSY-HSQC NMR spectra were recorded for each titration step. General line broadening was observed in the NMR spectra once the ligand:protein ratio exceeds 1:1; therefore, titration points are only analyzed up to this ratio.

The chemical shift perturbation Δδ for the ^1^H,^15^N-BEST-TROSY-HSQC was calculated from the ^1^H- and ^15^N-shifts according to Eq. 1^40^ using the spectra with 0 and 1 equivalent of tweezers, where Δδ_N_ and Δδ_H_ represent the chemical shift perturbation values of the amide nitrogen and proton, respectively:

1 $${\mathrm{{\Delta}}}\delta = \sqrt {{\mathrm{{\Delta}}}\delta _H^2 + \left( {0.154 \cdot {\mathrm{{\Delta}}}\delta _N} \right)^2}$$

Relative signal intensities *I*/*I*_0_ were obtained by dividing the intensities in the presence of 1 equivalent of each tweezer by the intensities in the absence of tweezers. Residues L6 and W10 whose signals overlap with other signals at the end of the titration were excluded from the intensity analysis. Spectra were processed with Topspin 3.5 (Bruker) and analyzed in CARA (version 1.9.1.7). Chemical shift perturbation and relative signal intensities were calculated from the raw chemical shift data and peak intensities using Excel 2016 (Microsoft) and plotted with GraphPad Prism 5.0.

NMR samples of Survivin120 mutants to assess proper folding contained 100–500 µM unlabeled Survivin120 mutants in NMR buffer (50 mM KPi pH 6.5, 90 mM KCl, 2 mM DTT with 10% D_2_O). 1D proton spectra with water suppression were recorded and protein folding was evaluated based on the dispersion of amide, aromatic, and methyl signals. Folded proteins show a wide signal dispersion in the amide/aromatic range (6–10 ppm) and the presence of methyl signals at <1 ppm. By reason of the high demand for isotope-labeled protein, all NMR experiments were by default performed only once.

### Fluorescence anisotropy measurements

Fluorescence anisotropy was measured with a Jasco Spectrofluorometer FP-8300 in PBS buffer, pH 7.4, at 25 °C and data were collected with the software Spectra Manager™.

For the quantitative analysis of the Survivin120/CRM1 complex, Survivin120 was labeled with ATTO488-maleimide from Jenabioscience according to the manufacturer’s instructions and mixed with CRM1_1-1062VLV430AAA_ in a ratio of 1:5. The protein complex was then titrated with tweezers in several steps until a final concentration of approximately 180 μM tweezers was reached. Data were transformed to logarithmic scale and IC_50_ was fitted using the equation in GraphPad Prism 8:

2 $$y = A_{\rm{min}} + \frac{{\left( {A_{\rm{max}} - A_{\rm{min}}} \right)}}{{1 + 10^{x - \log \left( {IC_{50}} \right)}}}$$

where *A*_max_ is the anisotropy in the absence of tweezer, *A*_min_ is the anisotropy at the end titration, and *x* is the concentration of tweezer. *A*_max_ was constrained for each data set, whereas *A*_min_ and IC_50_ were fitted.

For binding studies, FAM-labeled molecular tweezers (200 nM) were titrated with Survivin120 wildtype or K90/103T mutant until a final concentration of 180 µM (wildtype) or 350 µM (K90/103T) was reached. Data were normalized to the measured anisotropy *A*_0_ in the absence of protein. Using a single-site binding model, the fluorescence anisotropy data were fitted to the equation:

3 $$y = {\mathrm{A}} \cdot \frac{{\left( {L + x + K_D} \right) - \sqrt {\left( {L + x + K_D} \right)^2 - 4 \cdot x \cdot L} }}{{2 \cdot L}}$$

where *A* is the anisotropy at saturation, *L* is the concentration of the fluorescent ligand, *x* is the concentration of protein titrated, and *K*_*D*_ is the dissociation constant.

### Statistical analysis

ITC thermograms were fitted to a one set of sites model with the software Origin provided with the instrument. Heat of dilution was subtracted as constant from each data point. From three independent experiments (*n* = 3), one representative example was depicted, and thermodynamic data were derived thereof. For pull-down experiments, only the representative quantification of the depicted western blot is shown. Of note, low protein yields of the mutant CRM1_1-1062VLV430AAA did not allow replicates of the respective experiment. Fluorescence anisotropy graphs were generated with GraphPad Prism 5.0. Depicted error bars represent the standard deviation of three independent titrations.

### Computational details

For MD simulations of full-length Survivin, an initial structure for Survivin was generated in Modeller v9.17^45^ using the Uniprot protein sequence O15392-1 [https://www.uniprot.org/uniprot/O15392] (human BIRC5 isoform alpha) as target and PDB entries 1E31 [https://www.rcsb.org/structure/1E31]^46^, 1F3H [https://www.rcsb.org/structure/1F3H]^47^, 3UEG [https://www.rcsb.org/structure/3UEG], 3UEH [https://www.rcsb.org/structure/3UEH], 3UEI [https://www.rcsb.org/structure/3UEI]^48^, and 1XOX [https://www.rcsb.org/structure/1XOX]^38^ as templates. The best model was selected to minimize the DOPE and molpdf scores and was validated with PROCHECK (v.3.5.4)^49^ from the online Swiss-Model Workspace^50^. The root-mean-square deviation (RMSD) between the model and templates is less than 0.7 Å for the crystal structures (1E31, 1F3H, 3UEG, 3UEH, 3UEI) and 2.0 Å for the NMR solution (1XOX).

MD simulations were run with Gromacs 4.6.7^51^ using the Amber ff99SB force field^52^ extended with ZAFF to model the zinc finger^53^. Topology files were created with the TLEaP module of Amber v12.21^54^ and converted to Gromacs topologies by ACPype^55^. Proteins were solvated in a dodecahedron box of SPC/E water molecules^56^ with a 10 Å minimum separation between the protein and the box boundaries. The system was neutralized by addition of Na^+^ and Cl^−^ ions to a final ionic strength of 0.15 mol/L. The system was energy-minimized by steepest-descent to a total force of 2000, equilibrated for 5 ns in the NVT ensemble with restrained heavy atoms, and for 5 ns in the NPT ensemble without restraints. Production simulations were run in the NPT ensemble for a total of 310 ns (3 × 50 ns, 2 × 80 ns). Temperature was stabilized at 300 K in the NVT and NPT ensembles by the V-rescale thermostat^57^, while the pressure was stabilized at 1 atm in the NPT ensemble by the Berendsen barostat (equilibration) or Parrinello–Rahman barostat (data production)^58^. Simulations were carried out on a GPU (GeForce 970 and GeForce 1070, CUDA 6.5) using a time step of 2  fs, the Verlet scheme^59^ for neighbor search with a 10 Å cutoff, the Particle Mesh Ewald method^60^ for electrostatic calculations, and the LINCS algorithm^61^ for bond constraints.

Representative structures were extracted from trajectories based on mutual RMSDs of the backbone atoms, using the g_rms tool in Gromacs to produce 2D RMSD plots, the PAM (partition around medoids)^62^ tool from R package cluster, version 2.0.6, in R v3.3.1^63^ to find clusters, and the cluster.stats function of R package fpc, version 2.1.10, to validate the clustering based on silhouette coefficients^64^. For each cluster, the MD frame that minimized the RMSD with all other frames in the cluster was selected as the representative pose of that cluster. Root-mean-square fluctuations were calculated residue-wise on the concatenated MD trajectories using the g_rms tool in Gromacs.

For MD simulations of monomeric and dimeric Survivin120, NAMD^65^ was used to perform 80 ns (2 × 40 ns) MD simulations of 1:1 protein–tweezer complexes with the ligand (TW and TW-ELTL) on K23, K90, K91, and K103 (SI11). The simulations were performed in the NPT ensemble at 1 atm and 300 K^74^ with the CHARMM36m force field^66,67^. The system was placed in a TIP3P^68^ water box built with a padding of 20 Å and neutralized with sodium ions. A cutoff of 12 Å was used for Van der Waals interactions. Long-range electrostatic contributions were evaluated using the Particle Mesh Ewald method^60^. The systems were initially minimized and equilibrated at 300 K by performing 150 ps each of NVT and NPT simulations with a time step of 2 fs. Harmonic constraints on the collective variables representing distances and angles were used to maintain the geometry of the tetrahedral zinc finger. As in previous studies, the conformational features of the lysine–tweezers complexes are conserved (Table SI12).

QM/MM optimizations were performed using ChemShell^69^ with the DL-FIND geometry optimizer^70^ and Turbomole^71^ to handle the QM region. The QM region was formed by the tweezer as well as the ammonium and the methylene groups in positions *δ* and *ε* of the lysine´s sidechain. The MM region comprised the remaining protein atoms, solvent and ions. An electrostatic embedding scheme^72,73^ was used. The MM region was calculated with the CHARMM36m force field and the QM region at the DFT(B3LYP-D3)/Def2SVP level of theory^74^. Five snapshots from the MD simulations were used as initial geometries for QM/MM optimizations. The snapshots correspond to geometries around the centroid of the largest cluster from the MD simulations. The cluster analysis was performed using a quality-threshold-based algorithm implemented in VMD^75^, with a RMSD cutoff of 3 Å.

Additional information and computational details can be found in the supplementary information (SI10).

GaMD^76^ simulations were performed with NAMD using an analogous setup to the standard MDs. The statistics for the biasing potential were collected during 50 ns of equilibration prior the production run, which was then extended to 100 ns. The threshold value for the biasing potential was fixed at the maximum potential energy sampled during the equilibration step. The standard deviation of the biasing potential was controlled by allowing a maximum value of 10 kT.

### Reporting Summary

Further information on research design is available in the Nature Research Reporting Summary linked to this article.

## Supplementary information

Supplementary Information

Reporting Summary

## Data Availability

Data supporting the findings of this manuscript are available from the corresponding authors upon reasonable request. A reporting summary for this article is available as a Supplementary Information file. Source data are provided with this paper.

## Source data

Source Data

## References

[1] Keskin O, Tuncbag N, Gursoy A (2016). Predicting protein-protein interactions from the molecular to the proteome level. Chem. Rev..

[2] Kubota R, Hamachi I (2015). Protein recognition using synthetic small-molecular binders toward optical protein sensing in vitro and in live cells. Chem. Soc. Rev..

[3] Scott DE, Bayly AR, Abell C, Skidmore J (2016). Small molecules, big targets: drug discovery faces the protein-protein interaction challenge. Nat. Rev. Drug Disco..

[4] van Dun S, Ottmann C, Milroy LG, Brunsveld L (2017). Supramolecular chemistry targeting proteins. J. Am. Chem. Soc..

[5] McGovern RE, Fernandes H, Khan AR, Power NP, Crowley PB (2012). Protein camouflage in cytochrome c-calixarene complexes. Nat. Chem..

[6] Chinai JM (2011). Molecular recognition of insulin by a synthetic receptor. J. Am. Chem. Soc..

[7] Bier D (2013). Molecular tweezers modulate 14-3-3 protein-protein interactions. Nat. Chem..

[8] Hatai J, Schmuck C (2019). Diverse properties of guanidiniocarbonyl pyrrole-based molecules: artificial analogues of arginine. Acc. Chem. Res.

[9] Bier D (2017). The molecular tweezer CLR01 stabilizes a disordered protein-protein interface. J. Am. Chem. Soc..

[10] de Vink PJ (2017). A binary bivalent supramolecular assembly platform based on cucurbit[8]uril and dimeric adapter protein 14-3-3. Angew. Chem. Int Ed. Engl..

[11] Nguyen HD, Dang DT, van Dongen JL, Brunsveld L (2010). Protein dimerization induced by supramolecular interactions with cucurbit[8]uril. Angew. Chem. Int Ed. Engl..

[12] Trusch F (2018). Cell entry of a host-targeting protein of oomycetes requires gp96. Nat. Commun..

[13] Vallet C (2020). Functional disruption of the cancer-relevant interaction between survivin and histone H3 with a guanidiniocarbonyl pyrrole ligand. Angew. Chem. Int Ed. Engl..

[14] Mallon M, Dutt S, Schrader T, Crowley PB (2016). Protein camouflage: aupramolecular anion recognition by ubiquitin. ChemBioChem.

[15] Smith LC, Leach DG, Blaylock BE, Ali OA, Urbach AR (2015). Sequence-specific, nanomolar peptide binding via cucurbit[8]uril-induced folding and inclusion of neighboring side chains. J. Am. Chem. Soc..

[16] McGovern RE (2015). Structural study of a small molecule receptor bound to dimethyllysine in lysozyme. Chem. Sci..

[17] Sonzini S (2016). High affinity recognition of a selected amino acid epitope within a protein by cucurbit[8]uril complexation. Angew. Chem. Int Ed. Engl..

[18] Ambrosini G, Adida C, Altieri DC (1997). A novel anti-apoptosis gene, survivin, expressed in cancer and lymphoma. Nat. Med.

[19] Adida C (2000). Prognostic significance of survivin expression in diffuse large B-cell lymphomas. Blood.

[20] Capalbo G (2007). The role of survivin for radiation therapy. Prognostic and predictive factor and therapeutic target. Strahlenther. Onkol..

[21] Chen P (2014). Over-expression of survivin and VEGF in small-cell lung cancer may predict the poorer prognosis. Med Oncol..

[22] Engels K (2007). Dynamic intracellular survivin in oral squamous cell carcinoma: underlying molecular mechanism and potential as an early prognostic marker. J. Pathol..

[23] Xu C (2014). High survivin mRNA expression is a predictor of poor prognosis in breast cancer: a comparative study at the mRNA and protein level. Breast Cancer.

[24] Knauer SK, Mann W, Stauber RH (2007). Survivin’s dual role: an export’s view. Cell Cycle.

[25] Li F (1998). Control of apoptosis and mitotic spindle checkpoint by survivin. Nature.

[26] Knauer SK, Bier C, Habtemichael N, Stauber RH (2006). The Survivin-Crm1 interaction is essential for chromosomal passenger complex localization and function. EMBO Rep..

[27] Knauer SK (2007). The survivin isoform survivin-3B is cytoprotective and can function as a chromosomal passenger complex protein. Cell Cycle.

[28] Knauer SK (2007). Nuclear export is essential for the tumor-promoting activity of survivin. FASEB J..

[29] Fokkens M, Schrader T, Klarner FG (2005). A molecular tweezer for lysine and arginine. J. Am. Chem. Soc..

[30] Talbiersky P, Bastkowski F, Klarner FG, Schrader T (2008). Molecular clip and tweezer introduce new mechanisms of enzyme inhibition. J. Am. Chem. Soc..

[31] Wilch C (2017). Molecular tweezers inhibit PARP-1 by a new mechanism. Eur. J. Org. Chem..

[32] Schrader T, Bitan G, Klarner FG (2016). Molecular tweezers for lysine and arginine—powerful inhibitors of pathologic protein aggregation. Chem. Commun. (Camb.).

[33] Vopel T (2017). Inhibition of huntingtin exon-1 aggregation by the molecular tweezer CLR01. J. Am. Chem. Soc..

[34] Trusch F (2016). Molecular tweezers target a protein-protein interface and thereby modulate complex formation. Chem. Commun. (Camb.).

[35] Engelsma D, Rodriguez JA, Fish A, Giaccone G, Fornerod M (2007). Homodimerization antagonizes nuclear export of survivin. Traffic.

[36] Pavlyukov MS (2011). Survivin monomer plays an essential role in apoptosis regulation. J. Biol. Chem..

[37] Heid C (2018). Molecular tweezers with additional recognition sites. Chemistry.

[38] Sun C, Nettesheim D, Liu Z, Olejniczak ET (2005). Solution structure of human survivin and its binding interface with Smac/Diablo. Biochemistry.

[39] Dutt S (2013). Molecular tweezers with varying anions: a comparative study. J. Org. Chem..

[40] García-Santisteban I (2016). A cellular reporter to evaluate CRM1 nuclear export activity: functional analysis of the cancer-related mutant E571K. Cell. Mol. Life Sci..

[41] Sowislok A. A. Ein neuer Weg zu asymmetrischen Klammern und Pinzetten mit Phosphoramiditen. Universität Duisburg-Essen (2019).

[42] Li H, Aneja R, Chaiken I (2013). Click chemistry in peptide-based drug design. Molecules.

[43] Lallana E, Riguera R, Fernandez‐Megia E (2011). Reliable and efficient procedures for the conjugation of biomolecules through Huisgen azide–alkyne cycloadditions. Angew. Chem. Int Ed. Engl..

[44] Keller R. L. The computer aided resonance assignment tutorial. CANTINA Verlag 1st ed. (2004).

[45] Sali A, Blundell TL (1993). Comparative protein modelling by satisfaction of spatial restraints. J. Mol. Biol..

[46] Chantalat L (2000). Crystal structure of human survivin reveals a bow tie-shaped dimer with two unusual alpha-helical extensions. Mol. Cell.

[47] Verdecia MA (2000). Structure of the human anti-apoptotic protein survivin reveals a dimeric arrangement. Nat. Struct. Biol..

[48] Niedzialkowska E (2012). Molecular basis for phosphospecific recognition of histone H3 tails by Survivin paralogues at inner centromeres. Mol. Biol. Cell.

[49] Laskowski RA, MacArthur MW, Moss DS, Thornton JM (1993). PROCHECK: a program to check the stereochemical quality of protein structures. J. Appl. Crystallogr..

[50] Arnold K, Bordoli L, Kopp J, Schwede T (2006). The SWISS-MODEL workspace: a web-based environment for protein structure homology modelling. Bioinformatics.

[51] Pronk S (2013). GROMACS 4.5: a high-throughput and highly parallel open source molecular simulation toolkit. Bioinformatics.

[52] Hornak V (2006). Comparison of multiple Amber force fields and development of improved protein backbone parameters. Proteins.

[53] Peters MB (2010). Structural survey of zinc containing proteins and the development of the Zinc AMBER Force Field (ZAFF). J. Chem. Theory Comput.

[54] Case, D. A. et al. AMBER 12. University of California, San Francisco (2012).

[55] Sousa da Silva AW, Vranken WF (2012). ACPYPE - AnteChamber PYthon Parser interfacE. BMC Res Notes.

[56] Berendsen HJC, Grigera JR, Straatsma TP (1987). The missing term in effective pair potentials. J. Phys. Chem..

[57] Bussi G, Donadio D, Parrinello M (2007). Canonical sampling through velocity rescaling. J. Chem. Phys..

[58] Parrinello M, Rahman A (1981). Polymorphic transitions in single crystals: a new molecular dynamics method. J. Appl. Phys..

[59] Páll S, Hess B (2013). A flexible algorithm for calculating pair interactions on SIMD architectures. Computer Phys. Commun..

[60] Darden T, York D, Pedersen L (1993). Particle mesh Ewald: an N log(N) method for Ewald sums in large systems. J. Chem. Phys..

[61] Hess B, Bekker H, Berendsen HJC, Fraaije JGEM (1997). LINCS: a linear constraint solver for molecular simulations. J. Computational Chem..

[62] Kaufman L., Rousseeuw P. J. Clustering by means of medoids. 405–416 (1987).

[63] R Core Team. R: a language and environment for statistical computing. Available at www.R-project.org (2016).

[64] Rousseeuw P (1987). Silhouettes: a graphical aid to the interpretation and validation of cluster analysis. J. Computational Appl. Math..

[65] Phillips JC (2005). Scalable molecular dynamics with NAMD. J. Comput Chem..

[66] Klauda JB (2010). Update of the CHARMM all-atom additive force field for lipids: validation on six lipid types. J. Phys. Chem. B.

[67] Vanommeslaeghe K (2010). CHARMM general force field: a force field for drug-like molecules compatible with the CHARMM all-atom additive biological force fields. J. Comput Chem..

[68] Mark P, Nilsson L (2001). Structure and dynamics of the TIP3P, SPC, and SPC/E water models at 298 K. J. Phys. Chem. A.

[69] Sherwood P (2003). QUASI: a general purpose implementation of the QM/MM approach and its application to problems in catalysis. J. Mol. Struc-Theochem..

[70] Kastner J (2009). DL-FIND: an open-source geometry optimizer for atomistic simulations. J. Phys. Chem. A.

[71] Ahlrichs R, Bär M, Häser M, Horn H, Kölmel C (1989). Electronic structure calculations on workstation computers: the program system turbomole. Chem. Phys. Lett..

[72] Lin H, Truhlar DG (2006). QM/MM: what have we learned, where are we, and where do we go from here?. Theor. Chem. Acc..

[73] Cisneros GA, Piquemal JP, Darden TA (2006). Quantum mechanics/molecular mechanics electrostatic embedding with continuous and discrete functions. J. Phys. Chem. B.

[74] Grimme S (2011). Density functional theory with London dispersion corrections. WIREs Computational Mol. Sci..

[75] Humphrey W, Dalke A, Schulten K (1996). VMD: Visual molecular dynamics. J. Mol. Graph..

[76] Pang YT, Miao Y, Wang Y, McCammon JA (2017). Gaussian accelerated molecular dynamics in NAMD. J. Chem. Theory Comput.

