# Supplementary material for: Specific inhibition of the Survivin–CRM1 interaction by peptide-modified molecular tweezers
Source: Nat Commun. 2021 Mar 8;12:1505. doi: 10.1038/s41467-021-21753-9 (PMC7940618; doi:10.1038/s41467-021-21753-9)
Supplement: Supplementary file 1 — Supplementary Information [file 41467_2021_21753_MOESM1_ESM.pdf]

Supplementary Information

Supplementary Method SI1: Peptides

Azac-ELTL peptide:

^{1}H NMR (600 MHz, DMSO-d_6) δ 0.84 (dd, J = 6.6, 1.6 Hz, 6H, H-13d), 0.88 (d, J = 6.6 Hz, 6H, H-7d), 1.04 (d, J = 6.3 Hz, 3H, H-10c), 1.41 – 1.57 (m, 4H, H-7b, H-13b), 1.62 (ddt, J = 26.1, 13.5, 6.7 Hz, 2H, H-7c, H-13c), 1.73 (dt, J = 13.7, 7.8 Hz, 1H, H-4b), 1.89 (dq, J = 13.7, 8.1, 7.2 Hz, 1H, H-4b), 2.22 (t, J = 8.1 Hz, 2H, H-4c), 3.81 – 3.90 (m, 2H, H-1), 3.94 (d, J = 6.2 Hz, 1H, H-10b), 4.19 (dd, J = 8.5, 4.7 Hz, 1H, H-13a), 4.24 (q, J = 8.0 Hz, 1H, H-10a), 4.36 (m, 2H, H-4a, H7a), 7.83 (m, 1H, H-12), 7.85 (d, J = 8.6 Hz, 1H, H-9), 8.15 (d, J = 7.9 Hz, 1H, H-6), 8.25 (d, J = 8.1 Hz, 1H, H-3).

^{13}C NMR (151 MHz, DMSO) δ 19.63, 21.45, 21.68, 22.85, 23.02, 24.11, 24.13, 27.66, 30.07, 39.10, 39.24, 39.38, 39.52, 39.66, 39.79, 39.93, 40.05, 40.56, 50.50, 51.13, 51.76, 58.11, 66.53, 167.30, 169.81, 170.60, 171.84, 173.86, 173.93.

HRMS; m/z [M+H]$^+$: 558.2882 calc., 558.2901 obs.

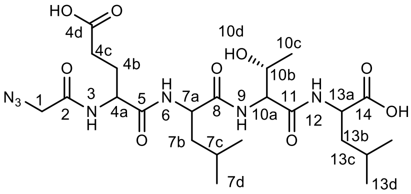

Chemical Formula: $C_{23}H_{39}N_7O_9$
Exact Mass: 557.2809

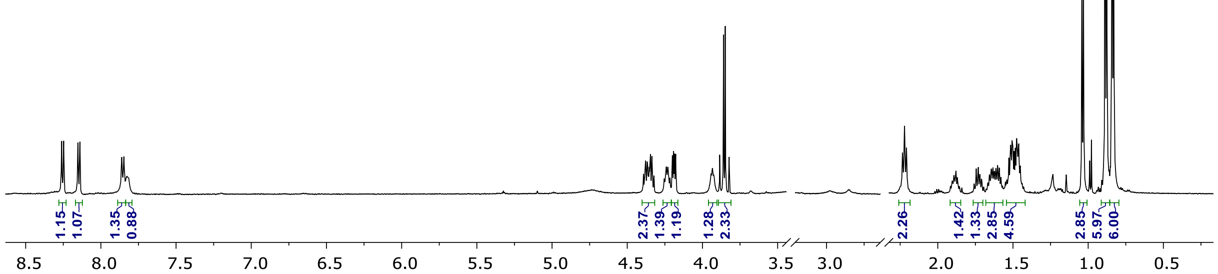

Azac-ELTLGEFL peptide:

^{1}H NMR (600 MHz, DMSO-d_6) δ 0.83-0.91 (m, 18H, H-7d, H-25d, H-13d), 1.01 (d, J = 6.2 Hz, 3H, H-10c), 1.41 – 1.77 (m, 12H, H-4b, H-7b, H-7c, H-13b, H-13c, H-25b, H-25c), 1.80 (h, J = 7.9, 7.0 Hz, 1H, H-19b), 1.89 (dq, J = 13.6, 8.1, 7.2 Hz, 1H, H-4b), 2.14 (t, J = 8.2 Hz, 2H, H-19c), 2.22 (t, J = 8.1 Hz, 2H, H-4c), 2.77 (dd, J = 14.0, 9.7 Hz, 1H, H-22b''), 3.03 (dd, J = 14.0, 4.3 Hz, 1H, H-22b'), 3.67 (ddd, J = 48.5, 16.6, 5.7 Hz, 2H, H-16a), 3.80 – 3.90 (m, 2H, H-1), 3.95 – 4.03 (m, 1H), 4.19 – 4.31 (m, 4H, H-7a, H-10a, H-19a), 4.31 – 4.41 (m, 2H, H-25a, H-4a), 4.51 (td, J = 9.0, 4.5 Hz, 1H, H-22a), 4.86 (s, 1H, OH), 7.13 – 7.28 (m, 5H, Aryl-H), 7.83 (dd, J = 13.2, 8.0 Hz, 3H, N-9, N-12, N-18), 8.02 – 8.12 (m, 2H, N-15, N-21), 8.15 (d, J = 7.8 Hz, 2H, N-6, N-24), 8.25 (d, J = 8.0 Hz, 1H, N-3).

^{13}C NMR (151 MHz, DMSO) δ 19.46, 21.28, 21.55, 21.59, 22.87, 23.03, 23.04, 24.02, 24.11, 24.21, 27.63, 27.67, 29.91, 30.03, 37.22, 39.10, 39.24, 39.38, 39.52, 39.66, 39.79, 39.93, 40.74, 41.91, 50.26, 50.50, 51.11, 51.18, 51.54, 51.74, 53.62, 57.82, 66.48, 126.22, 128.00, 129.17, 137.69, 167.31, 168.40, 169.82, 170.64, 170.69, 170.88, 171.98, 172.23, 173.84, 173.87, 173.99.

HRMS; m/z [M+H]$^{+}$: 1004.5047 calc., 1004.5059 obs.

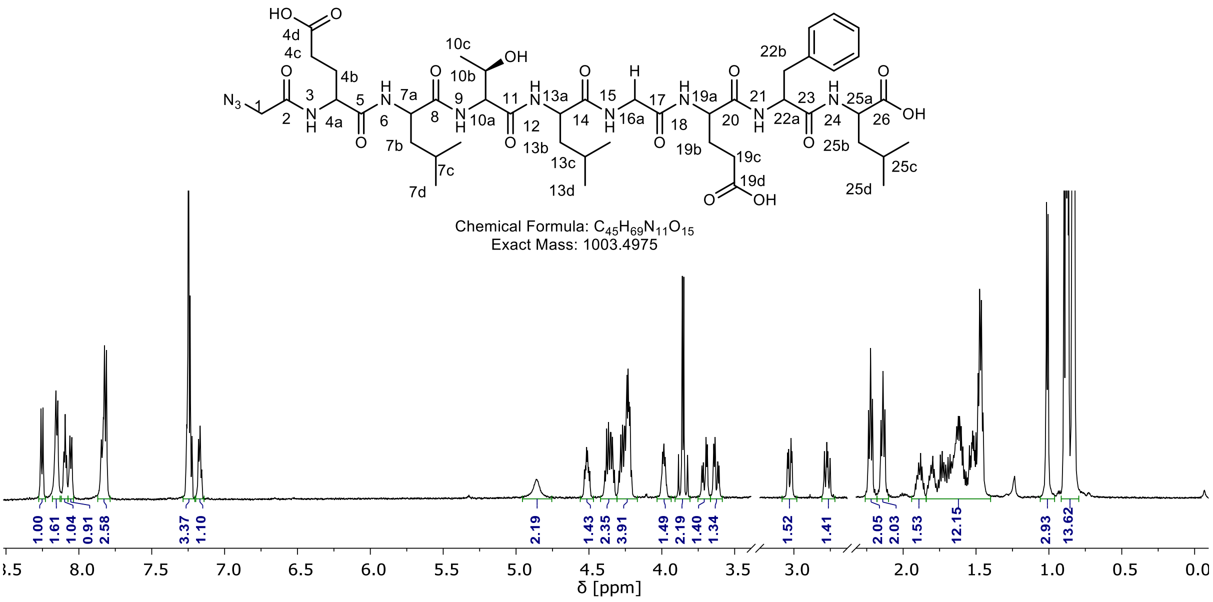

Azac-LFEEGLLT peptide:

^{1}H NMR (600 MHz, DMSO-*d*$_6$) δ 0.78 – 0.93 (m, 18H, H-4d, H-19d, H-22d), 1.03 (d, J = 6.3 Hz, 3H, H-25c), 1.31 – 1.54 (m, 7H, H-4b, H-19b, H-19c, H-22b), 1.54 – 1.67 (m, 2H, H-4c, H-22c), 1.68 – 1.84 (m, 2H, H-13b', H-10c') 1.85 – 1.98 (m, 2H, H-13b'', H-10c''), 2.16 – 2.32 (m, 4H, H-10b, H-13c), 2.81 (dd, J = 14.0, 9.7 Hz, 1H, H-7b'), 3.02 (dd, J = 14.1, 4.4 Hz, 1H, H-7b''), 3.65 (dd, J = 16.6, 5.7 Hz, 1H, H-16'), 3.73 – 3.87 (m, 3H, H-1, H-16''), 4.08 – 4.19 (m, 2H, H-25a, H-25b), 4.22 – 4.42 (m, 5H, H-4a, H-10a, H-13a, H-19a, H-22a), 4.51 (ddd, J = 9.7, 8.1, 4.5 Hz, 1H, H-7a), 7.14 – 7.27 (m, 5H, Aryl-H), 7.53 (d, J = 8.6 Hz, 1H, N-24), 7.92 (d, J = 8.2 Hz, 1H, N -18), 7.99 (d, J = 7.5 Hz, 1H, N -12), 8.04 (d, J = 7.8 Hz, 1H, N -9), 8.09 (d, J = 8.2 Hz, 1H, N -21), 8.16 (dd, J = 7.2, 4.5 Hz, 2H, N-3, N-15), 8.20 (d, J = 8.1 Hz, 1H, N -6).

^{13}C NMR (151 MHz, DMSO-*d*$_6$) δ 20.19, 21.57, 21.63, 21.69, 22.92, 23.01, 23.04, 24.09, 27.29, 27.51, 30.01, 30.08, 36.97, 40.06, 40.44, 40.89, 41.04, 41.81, 50.46, 50.90, 50.94, 51.03, 51.84, 51.92, 53.70, 57.38, 66.30, 126.18, 127.97, 129.15, 137.78, 167.07, 168.32, 170.83, 170.93, 171.58, 171.81, 171.89, 172.07, 173.97.

HRMS; m/z [M+H]$^+$: 1004.5047 calc., 1004.5048 obs.

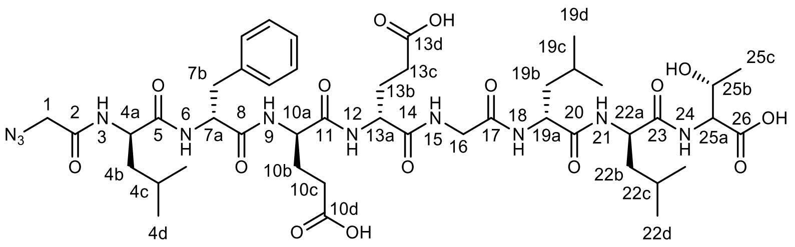

Chemical Formula: $C_{45}H_{69}N_{11}O_{15}$
Exact Mass: 1003.4975

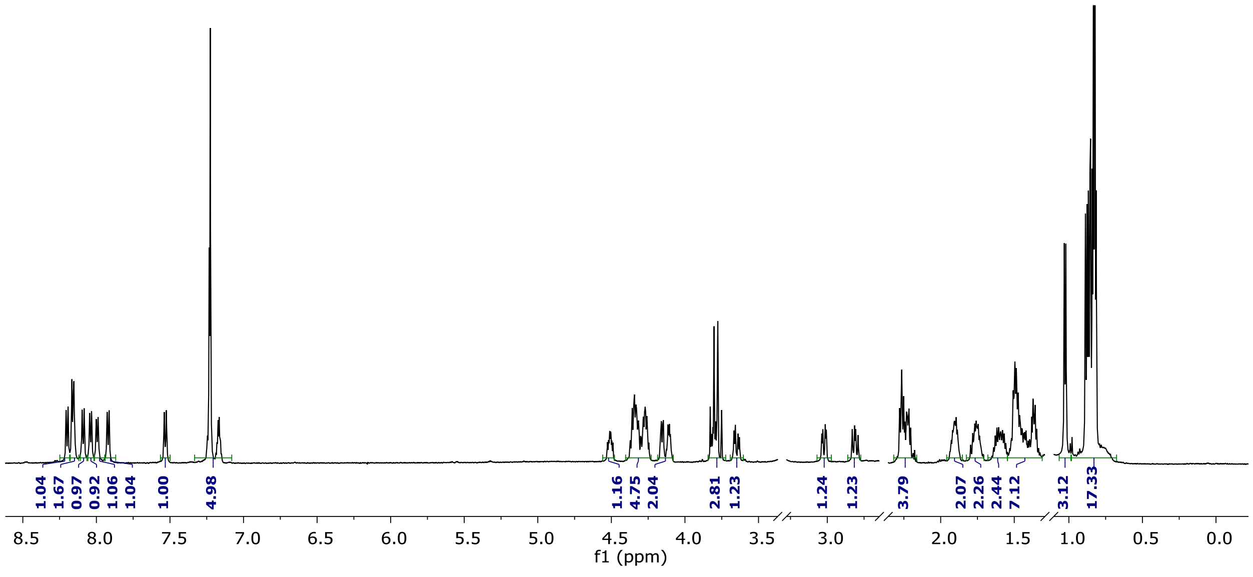

Supplementary Method SI2: Peptide-tweezer conjugates

TW-ELTL conjugate: Yield 8 mg, 6 µmol, 94 %.

^{1}H NMR (300 MHz, DMSO-d_6) δ 0.75 – 0.91 (m, 12H, Leu-CH_3), 1.04 (m, 3H), 1.24 (d, J = 8.6 Hz, 1H), 1.49 (d, J = 8.1 Hz, 4H), 2.15 – 2.34 (m, 10H), 2.93 (t, J = 6.9 Hz, 2H), 3.87 – 3.96 (m, 1H), 4.08 (m, 5H), 4.16 – 4.43 (m, 5H), 5.18 (s, 2H), 6.69 – 6.80 (m, 4H), 6.99 – 7.15 (m, 8H), 7.87 (m, 2H, NH), 7.96 (s, 1H, triazol-H), 8.19 (d, J = 7.7 Hz, 1H, NH), 8.57 (d, J = 7.8 Hz, 1H, NH).

^{31}P NMR (122 MHz, DMSO-d_6) δ -5.84, -5.41.

HRMS; m/z [M+Na]$^+$:1334.4695 calc., 1334.4640 obs.

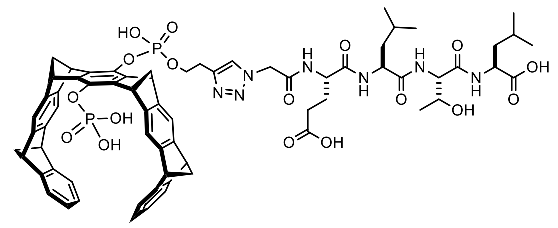

Chemical Formula: $C_{69}H_{75}N_7O_{17}P_2$
Exact Mass: 1335.4695

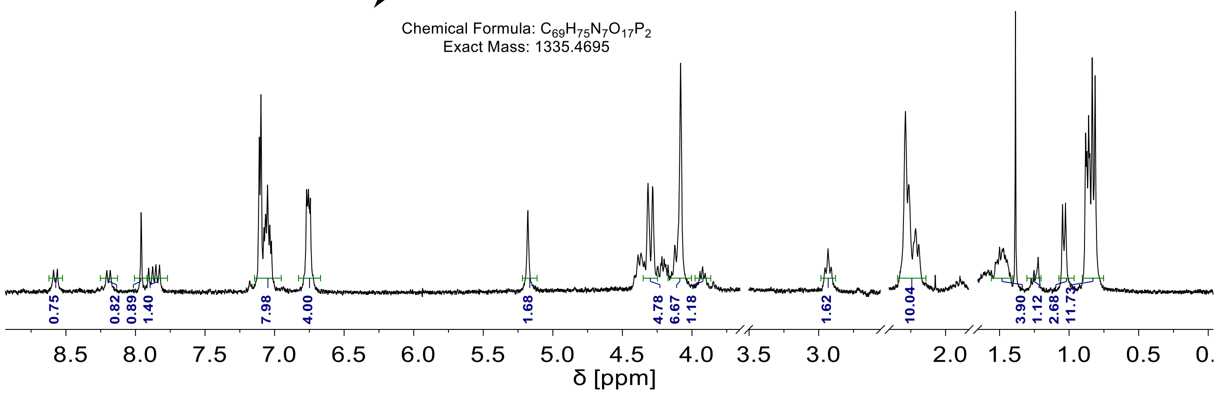

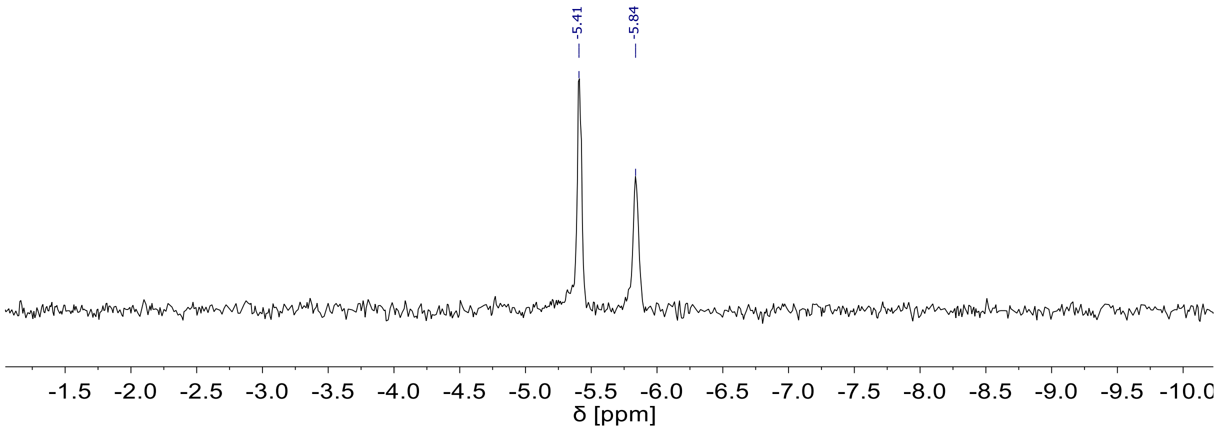

TW-ELTLGEFL conjugate: Yield 10 mg, 5.6 µmol, 94 %.

^{1}H NMR (600 MHz, DMSO-d_6) δ 0.85 (m, 18H), 0.89 (d, *J* = 6.6 Hz, 3H), 1.02 (d, *J* = 6.3 Hz, 3H), 1.48 (p, *J* = 7.6 Hz, 4H), 1.49 – 1.58 (m, 1H), 1.55 – 1.72 (m, 2H), 1.79 (ddd, *J* = 23.2, 11.8, 6.4 Hz, 2H), 1.93 (t, *J* = 8.1 Hz, 1H), 2.14 (t, *J* = 8.2 Hz, 2H), 2.21 (d, *J* = 7.1 Hz, 2H), 2.23 – 2.32 (m, 9H), 2.77 (dd, *J* = 14.0, 9.6 Hz, 1H), 2.92 (t, *J* = 6.9 Hz, 2H), 3.03 (dd, *J* = 14.0, 4.4 Hz, 1H), 3.62 (dd, *J* = 16.7, 5.8 Hz, 1H), 3.71 (dd, *J* = 16.6, 5.7 Hz, 1H), 3.97 – 4.02 (m, 1H), 4.08 (d, *J* = 1.5 Hz, 1H), 4.09 (s, 4H), 4.20 – 4.34 (m, 6H), 4.32 (d, *J* = 1.7 Hz, 2H), 4.39 (q, *J* = 7.5 Hz, 2H), 4.52 (td, *J* = 8.9, 4.3 Hz, 1H), 4.88 (s, 1H), 5.18 (s, 2H), 6.77 (tt, *J* = 5.3, 2.3 Hz, 4H), 7.02 – 7.22 (m, 10H), 7.21 – 7.28 (m, 4H), 7.80 – 7.88 (m, 3H), 7.96 (s, 1H), 8.05 (d, *J* = 8.3 Hz, 1H), 8.11 (t, *J* = 5.7 Hz, 1H), 8.18 (dd, *J* = 16.6, 7.9 Hz, 2H), 8.57 (d, *J* = 8.0 Hz, 1H).

^{13}C NMR (151 MHz, CDCl$_3$) δ 24.70, 26.50, 26.80, 26.87, 28.10, 28.27, 29.27, 29.37, 29.45, 31.98, 32.91, 33.14, 35.14, 35.29, 42.46, 45.30, 45.71, 45.99, 47.16, 53.18, 53.25, 55.45, 55.48, 55.53, 56.39, 56.44, 56.69, 56.78, 57.07, 58.84, 63.04, 71.76, 73.02, 74.24, 122.02, 126.70, 129.68, 131.45, 133.24, 134.42, 142.93, 146.20, 146.81, 147.91, 151.86, 152.18, 152.21, 155.48, 155.51, 170.67, 173.66, 175.09, 175.84, 175.94, 176.15, 177.23, 177.49, 179.08, 179.12, 179.23.

HRMS; m/z [M-H]$^-$:1780.6860 calc., 1780.6779 obs.

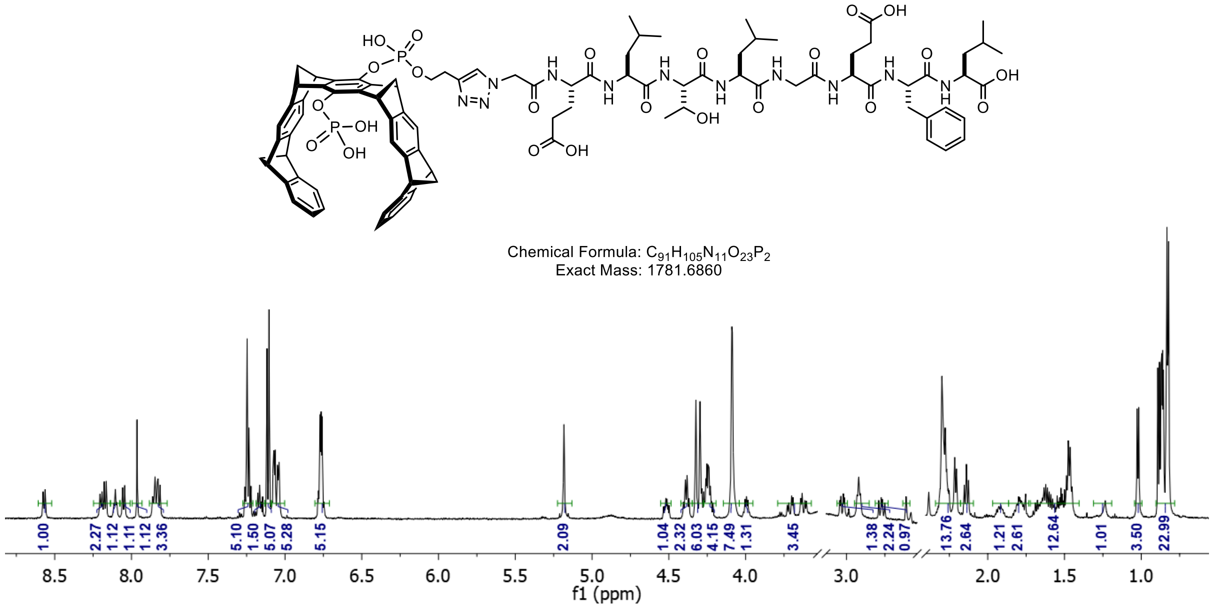

TW-LFEEGLLT conjugate: Yield 12 mg, 6.7 µmol, 96 %.

^{1}H NMR (600 MHz, DMSO-*d*$_6$) δ 0.79 – 0.93 (m, 18H), 1.03 (d, J = 6.4 Hz, 3H), 1.37 – 1.68 (m, 10H), 1.69 – 1.83 (m, 3H), 1.87 – 1.96 (m, 2H), 2.17 – 2.36 (m, 12H), 2.81 (dd, J = 14.0, 9.6 Hz, 1H), 2.94 (t, J = 6.8 Hz, 2H), 3.02 (dd, J = 14.1, 4.5 Hz, 1H), 3.65 (dd, J = 16.6, 5.5 Hz, 2H), 3.81 (dd, J = 16.6, 5.9 Hz, 2H), 4.06 – 4.15 (m, 7H), 4.17 (dd, J = 8.6, 3.1 Hz, 1H), 4.23 – 4.41 (m, 9H), 4.53 (td, J = 8.6, 4.5 Hz, 1H), 5.05 – 5.19 (m, 2H), 6.73 – 6.79 (m, 4H), 7.02 – 7.14 (m, 8H), 7.13 – 7.26 (m, 5H), 7.54 (d, J = 8.6 Hz, 1H), 7.92 (s, 1H), 7.95 (s, 1H), 8.00 (d, J = 7.6 Hz, 1H), 8.06 (d, J = 7.8 Hz, 1H), 8.09 (d, J = 8.2 Hz, 1H), 8.16 (t, J = 5.8 Hz, 1H), 8.27 (d, J = 8.0 Hz, 1H), 8.47 (d, J = 8.3 Hz, 1H).

^{13}C NMR (151 MHz, DMSO-*d*$_6$) δ 20.25 , 21.59, 21.70, 21.73, 22.98, 23.02, 23.04, 24.07, 24.10, 27.53, 30.01, 30.07, 40.06, 40.44, 41.06, 41.26, 41.81, 47.95, 48.02, 50.24, 50.29, 50.88, 51.01, 51.41, 51.82, 57.39, 66.34, 67.79, 69.03, 116.78, 116.86, 121.47, 124.32, 124.45, 126.21, 128.01, 129.16, 140.97, 141.65, 146.58, 146.62, 146.99, 150.28, 165.08, 168.33, 170.83, 170.94, 171.82, 171.89, 172.13, 173.98.

^{31}P NMR (122 MHz, DMSO-*d*$_6$) δ -5.85, -5.43.

HRMS; m/z [M+H]$^+$:1783.6864 calc., 1783.6979 obs.

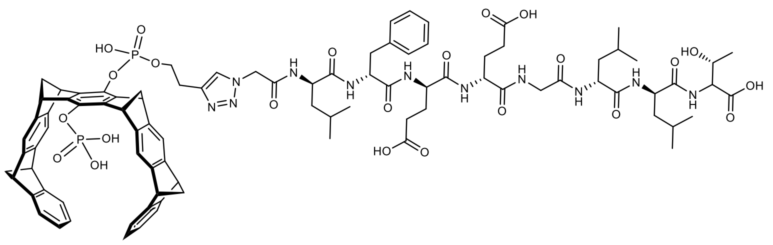

Chemical Formula: $C_{91}H_{105}N_{11}O_{23}P_2$
Exact Mass: 1781.6860

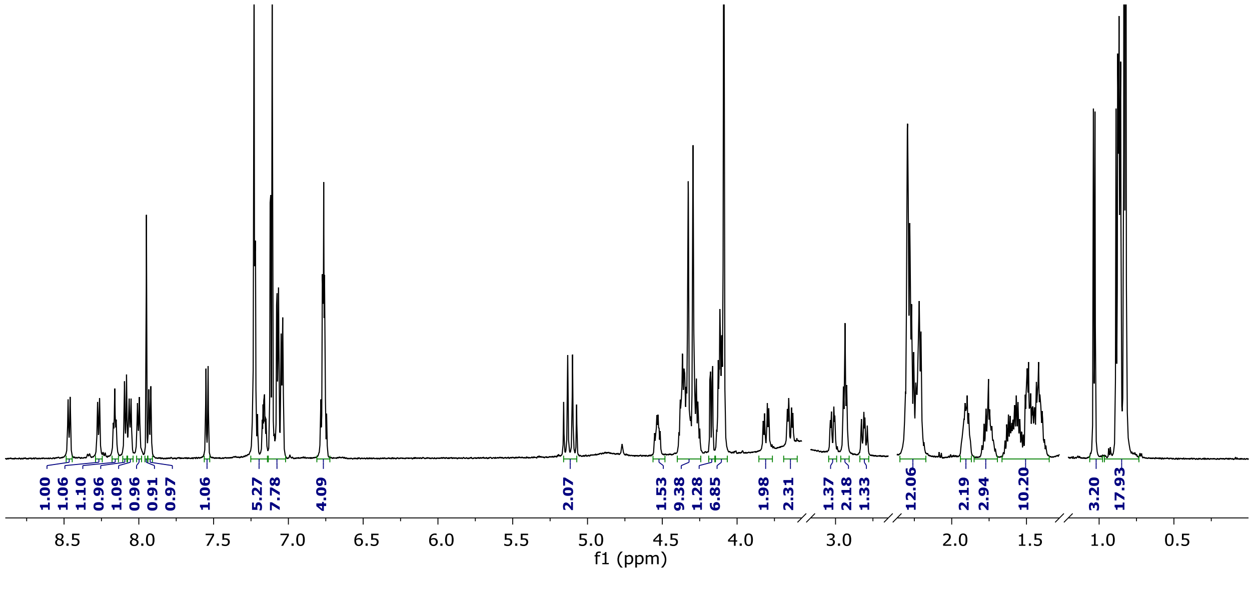

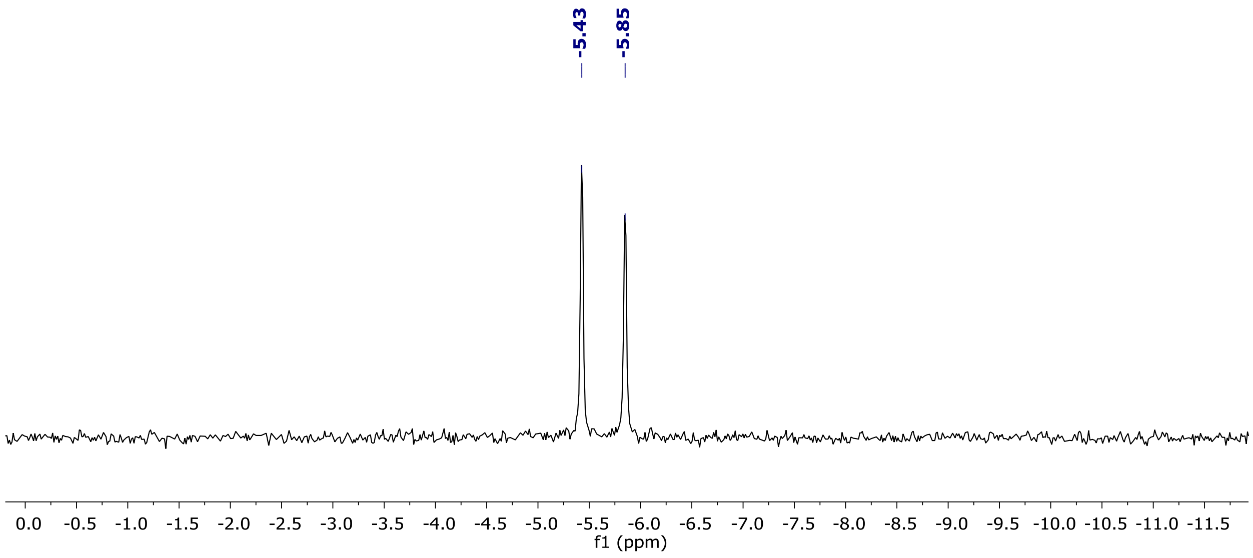
-5.43
-5.85
0.0 -0.5 -1.0 -1.5 -2.0 -2.5 -3.0 -3.5 -4.0 -4.5 -5.0 -5.5 -6.0 -6.5 -7.0 -7.5 -8.0 -8.5 -9.0 -9.5 -10.0 -10.5 -11.0 -11.5
f1 (ppm)

Supplementary Method SI3: FAM-labelled tweezers and peptide-tweezer conjugates

TW-FAM: Yield 16 mg, 11.24 µmol, 87 %.

^{1}H NMR (600 MHz, DMSO-*d*$_6$) δ 8.88 (s, 2H), 8.57 (s, 2H), 8.48 (s, 1H), 8.38 (s, 2H), 8.26 (d, J = 8.1 Hz, 1H), 8.06 (d, J = 8.2 Hz, 1H), 7.93 (s, 1H, triazol-H), 7.32 (s, 2H), 7.13 – 6.99 (m, 8H), 6.79 – 6.72 (m, 4H), 6.67 (s, 2H), 6.57 (s, 4H), 5.11 (s, 2H), 4.29 (s, 6H), 4.05 (t, J = 13.0 Hz, 5H), 3.95 (s, 2H), 3.73 (s, 2H), 3.57 (s, 1H), 2.83 (s, 3H), 2.37 – 2.10 (m, 10H), 2.00 (d, J = 12.0 Hz, 2H), 1.75 (s, 2H), 1.60 (s, 4H), 1.40 (s, 4H), 1.23 (s, 2H).

^{13}C NMR (151 MHz, DMSO-*d*$_6$) δ 168.70, 165.05, 160.23, 152.32, 150.75, 150.72, 150.67, 147.27, 136.86, 129.58, 126.96, 124.86, 124.62, 123.76, 121.88, 117.22, 116.99, 113.20, 102.73, 69.19, 68.14, 50.78, 50.75, 50.70, 48.48, 48.38, 41.15, 31.18, 25.60, 23.05.

HRMS; m/z [M+H]$^+$:1423.3752 calc., 1423.3790 obs.

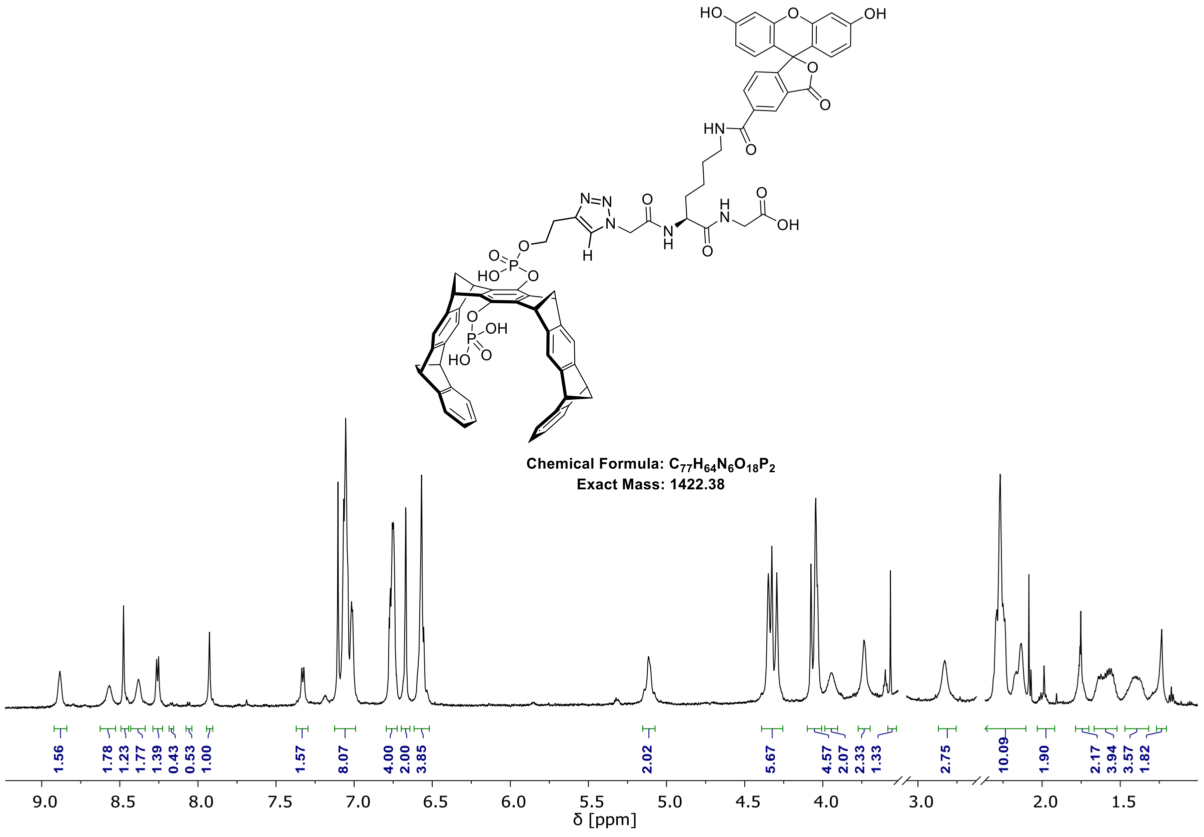

TW-ELTL-FAM: Yield 7 mg, 4 µmol, 31 %.

^{1}H NMR (600 MHz,) δ 10.20 (s, 2H), 8.81 (d, *J* = 5.6 Hz, 1H), 8.56 (d, J = 8.0 Hz, 1H), 8.46 (d, *J* = 1.7 Hz, 1H), 8.24 (dd, *J* = 8.1, 1.6 Hz, 1H), 8.20 (d, *J* = 8.0 Hz, 1H), 8.14 (t, *J* = 5.9 Hz, 1H), 7.96 (s, 1H, triazol-H), 7.90 (dd, *J* = 25.7, 9.0 Hz, 4H), 7.34 (d, *J* = 8.0 Hz, 1H), 7.12 – 6.98 (m, 9H), 6.79 – 6.72 (m, 4H), 6.69 (d, J = 2.2 Hz, 2H), 6.59 – 6.52 (m, 4H), 5.22 – 5.12 (m, 2H), 4.42 – 4.32 (m, 2H), 4.31 (s, 5H), 4.27 (s, 2H), 4.07 (t, J = 1.7 Hz, 4H), 4.01 – 3.96 (m, 1H), 3.78 (dd, J = 17.5, 5.9 Hz, 1H), 3.70 (dd, J = 17.5, 5.7 Hz, 1H), 2.86 (s, 2H), 2.28 (d, J = 11.2 Hz, 9H), 2.19 (d, J = 7.1 Hz, 2H), 1.93 (s, 2H), 1.82 – 1.74 (m, 1H), 1.71 (s, 1H), 1.60 (s, 2H), 1.56 (dt, J = 21.6, 6.9 Hz, 2H), 1.47 (s, 4H), 1.23 (s, 1H), 1.04 (d, J = 6.3 Hz, 3H), 0.87 – 0.79 (m, 11H).

^{13}C NMR (151 MHz, DMSO-*d*$_6$) δ 174.33, 170.17, 164.93, 160.09, 152.28, 150.73, 150.69, 136.85, 126.90, 124.89, 124.63, 121.92, 113.14, 109.55, 102.74, 83.72, 58.40, 50.76, 50.72, 48.47, 48.40, 41.06, 40.54, 30.59, 24.62, 24.52, 23.62, 23.51, 22.11, 21.97, 19.81.

^{31}P NMR (122 MHz, DMSO-*d*$_6$) δ -5.74, -5.39.

HRMS; m/z [M-H]$^-$:1877.6336 calc., 1877.6320 obs.

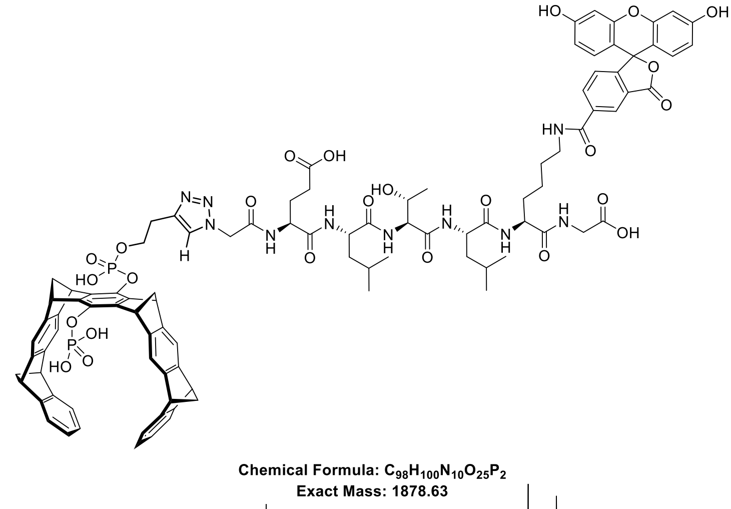

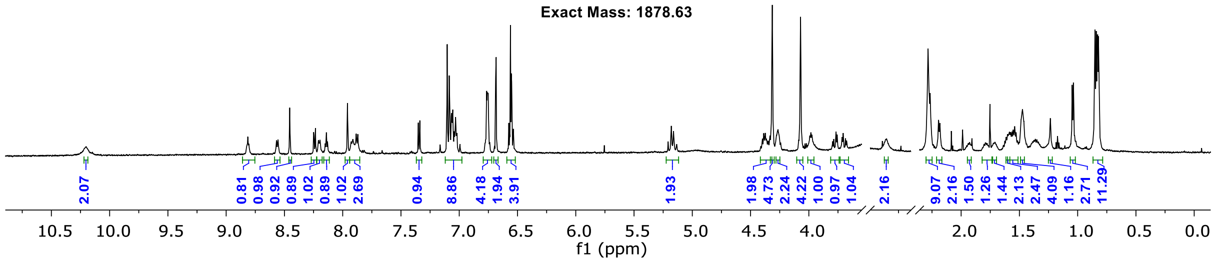

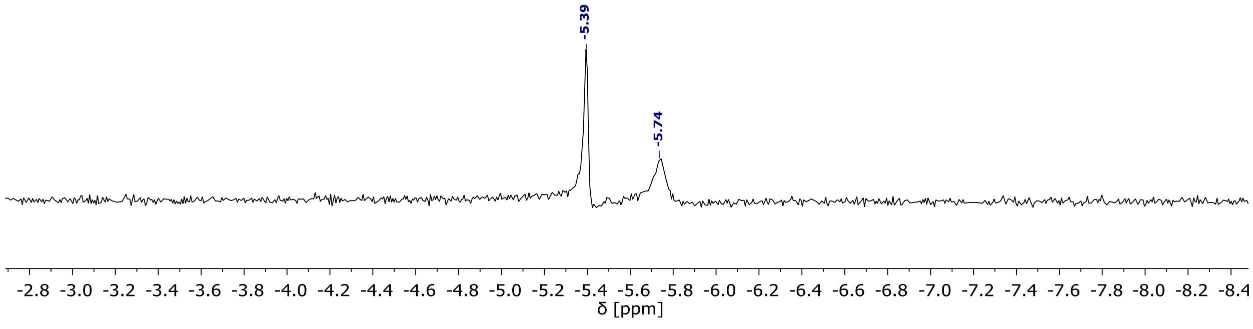

TW-ELTLGEFL-FAM. Yield 3 mg, 1.3 µmol, 10 %.

^{1}H NMR (600 MHz, DMSO-*d*$_6$) δ 8.83 (s, 1H), 8.46 (s, 1H), 8.28 – 8.22 (m, 3H), 8.12 (s, 1H), 7.95 (s, 1H, triazol-H), 7.23 (d, J = 13.4 Hz, 3H), 7.05 (s, 8H), 6.77 (s, 4H), 6.71 (s, 1H), 6.57 (s, 3H), 5.18 (s, 2H), 4.49 (s, 1H), 4.32 (s, 11H), 4.04 (s, 5H), 3.73 (s, 8H), 3.61 (s, 1H), 3.03 (s, 1H), 2.82 (s, 3H), 2.55 (s, 7H), 2.49 (s, 11H), 2.28 (s, 6H), 2.16 (s, 5H), 2.15 (d, J = 9.0 Hz, 2H), 1.82 (s, 2H), 1.72 (s, 2H), 1.58 (s, 14H), 1.25 (s, 4H), 1.07 (s, 2H), 0.83 (s, 11H).

^{13}C NMR (151 MHz, DMSO-*d*$_6$) δ 172.29, 172.16, 168.66, 164.91, 160.19, 152.31, 150.71, 150.61, 147.01, 138.23, 136.84, 126.91, 109.49, 83.76, 72.27, 68.72, 68.03, 67.15, 67.04, 66.82, 52.34, 42.58, 41.08, 37.50, 32.35, 30.52, 29.14, 25.59, 24.63, 23.94, 23.84, 23.76, 23.16, 22.56.

HRMS; m/z [M+H]$^{2+}$: 1163.9339 calc., 1163.9309 obs.

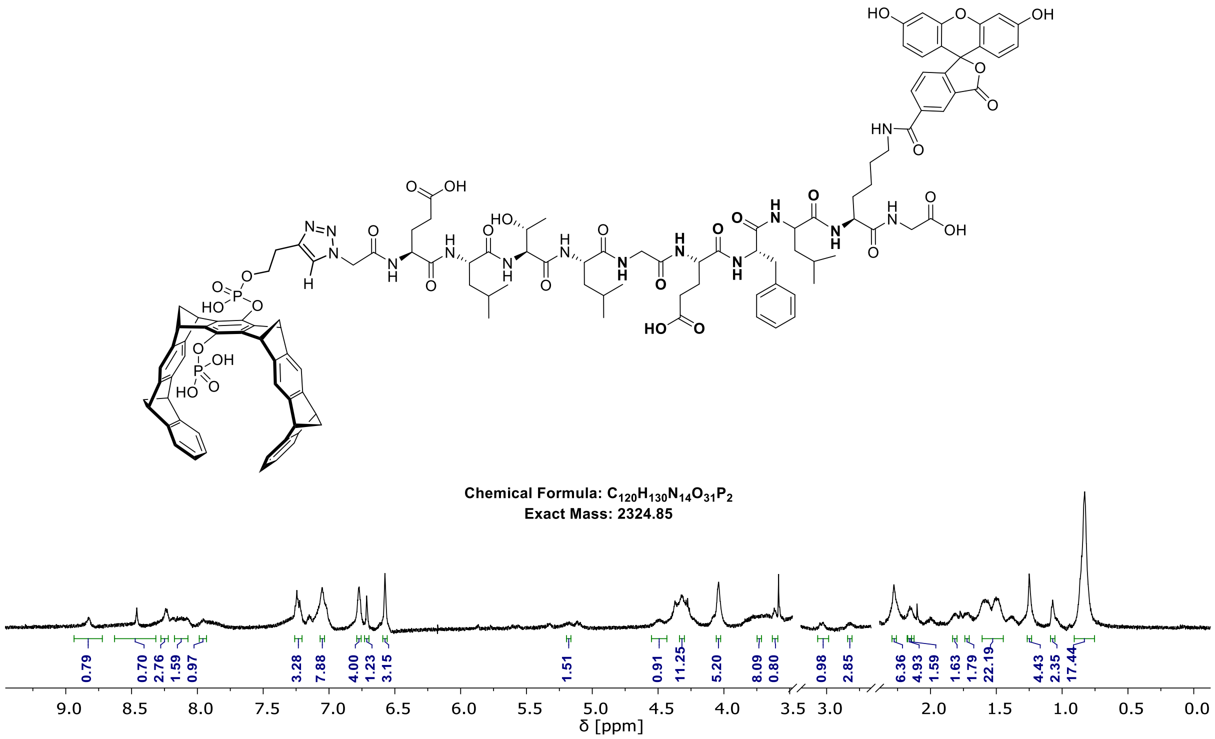

Supplementary Figure SI4

a

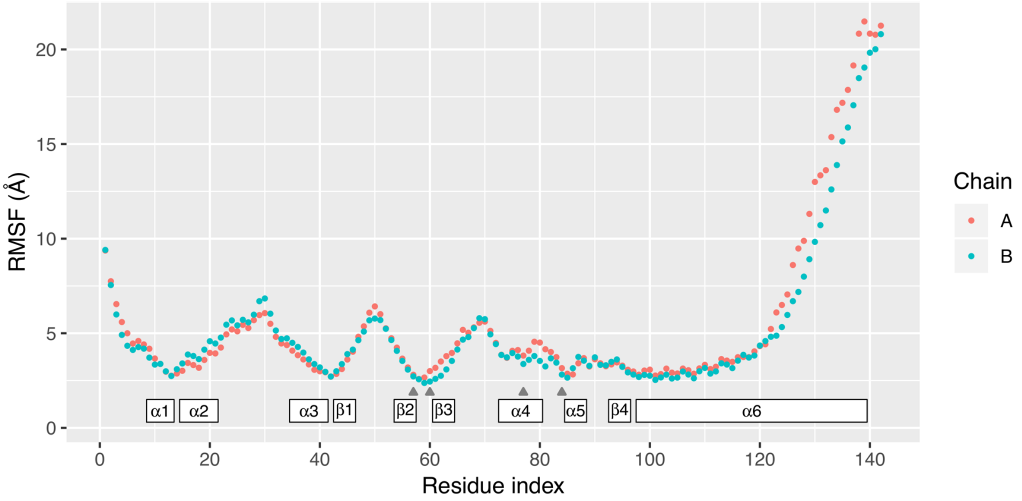

b

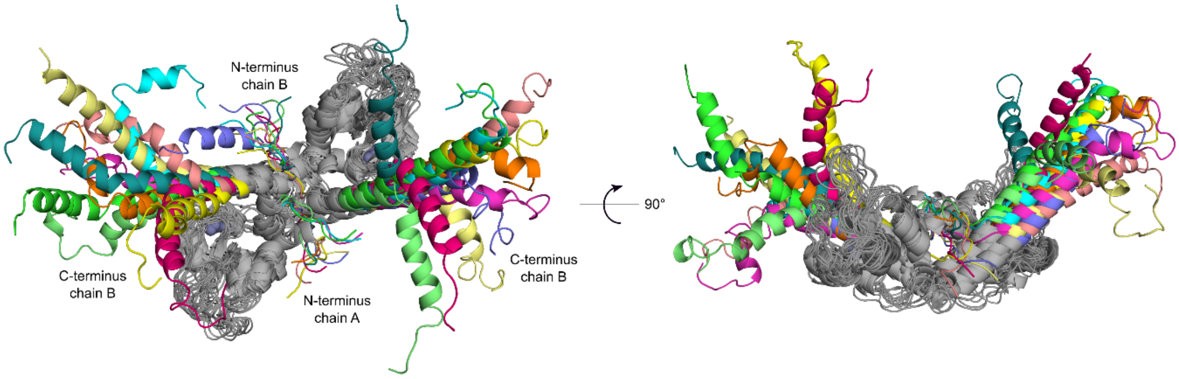

Supplementary Figure SI4: *In silico* simulation trajectories of Survivin protein dynamics. a) Analysis of the mobility of the Survivin dimer backbone atoms on the pooled MD trajectories. Dips in flexibility in the protein core (residues 6-90) are consistent with the known secondary structure of BIR domains[50], where five α-helices (white rectangles α1-5), a three-stranded antiparallel β-sheet (white rectangles β1-3) and a zinc finger (residues marked by grey triangles) contribute to the stability of the tertiary structure. Survivin extends the BIR domain with a C-terminal tail featuring a two-stranded intermolecular antiparallel β-sheet (β4) and an α-helix (α6). Although α6 forms a continuous 65 Å-long helix in crystal structures, this conformation is unstable in the MD simulation, where the second half of α6 (residues 120-142) is significantly more mobile than the N-terminal loop (residues 1-10). b) Superposition of the representative poses of the clustered MD trajectories, illustrating the range of motions of the C-terminal α6 helix. The relatively stable BIR domain is colored in grey with zinc ions represented as pastel blue spheres, while residues 1-7 and 109-142 have a unique color for each dimer cluster. In most clusters, the secondary structure of α6 features a turn near residue 120. The clusters are 12-56 ns long.

Supplementary Figure SI5

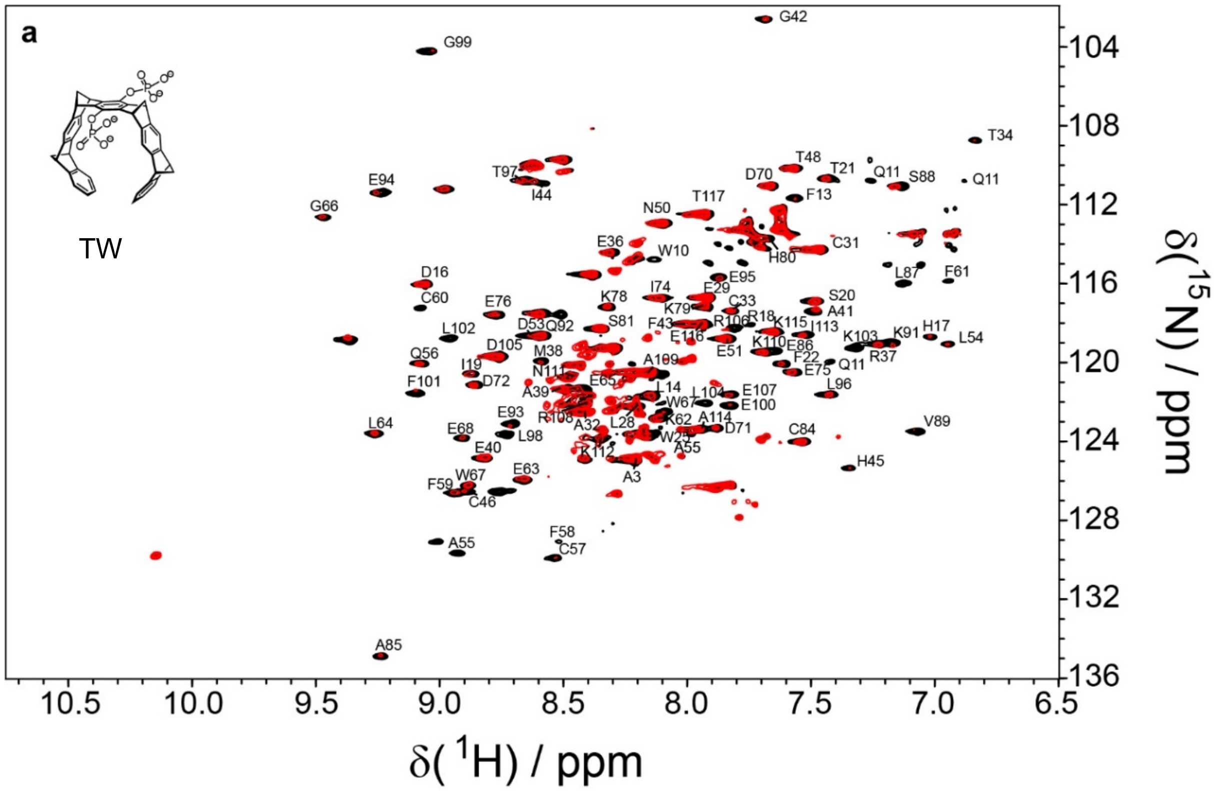

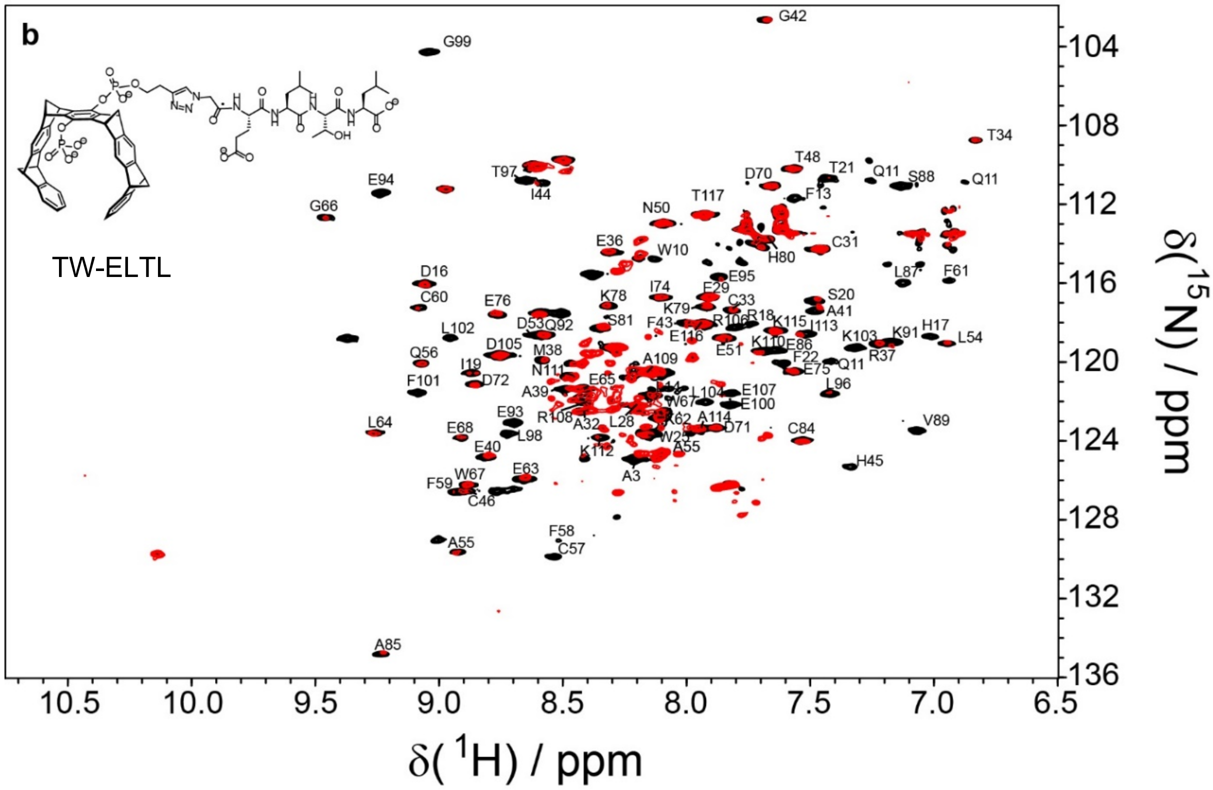

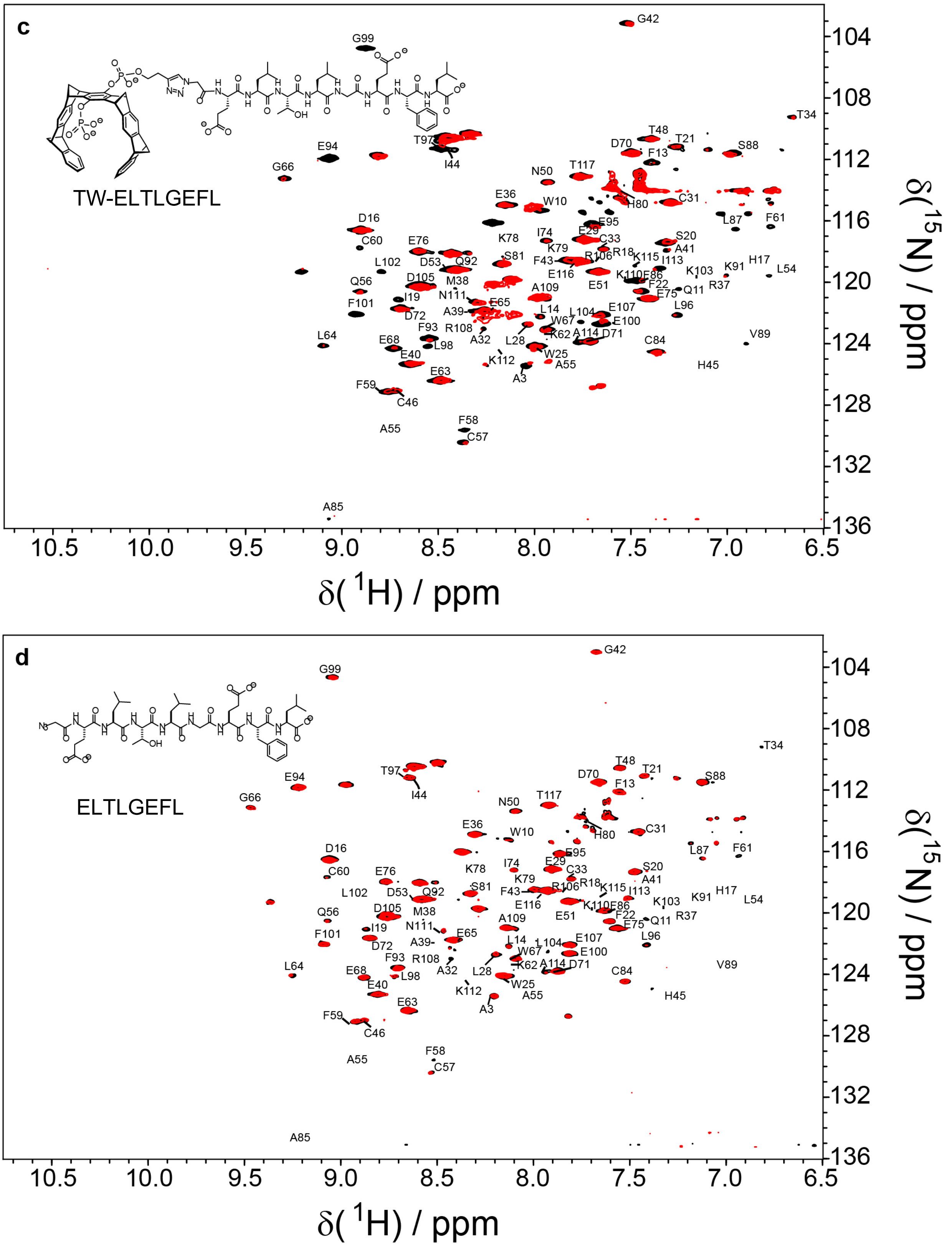
c
TW-ELTLGEFL
δ(¹H) / ppm
δ(¹⁵N) / ppm
d
ELTLGEFL
δ(¹H) / ppm
δ(¹⁵N) / ppm

e

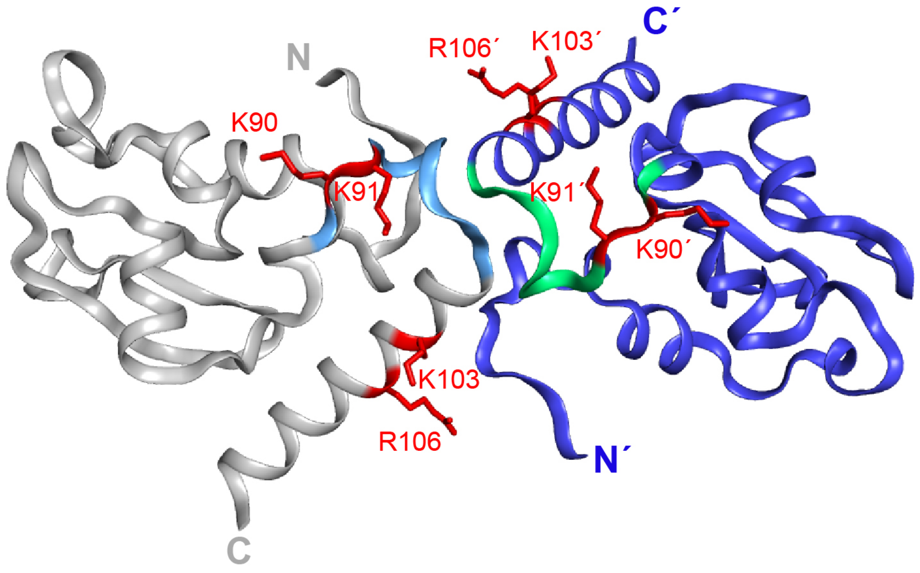

Supplementary Figure SI5: ^{15}N-HSQC NMR spectra of ^{15}N-Survivin120 in the absence (black) and presence (red) of tweezers. a) Unmodified tweezer TW, b) TW-ELTL, c) TW-ELTLGEFL, d) ELTLGEFL peptide (3.5-fold excess). Assigned signals are labeled. A shift or reduction of signal intensity is indicative of binding. No signal shifts or intensity changes are observed for the peptide-only control, indicating no binding. e) K and R residues displaying significant chemical shift perturbation and reduced signal intensities (red sticks) mapped to the structure of the Survivin120 dimer (PDB-ID: 1XOX [https://www.rcsb.org/structure/1xox]). The two protomers are shown in gray and blue, with the NES in light blue or green, respectively. Residues marked with a prime belong to the second protomer (blue). K90, K91, K103 as well as R106 are in close proximity to Survivin's NES.

Supplementary Figure SI6

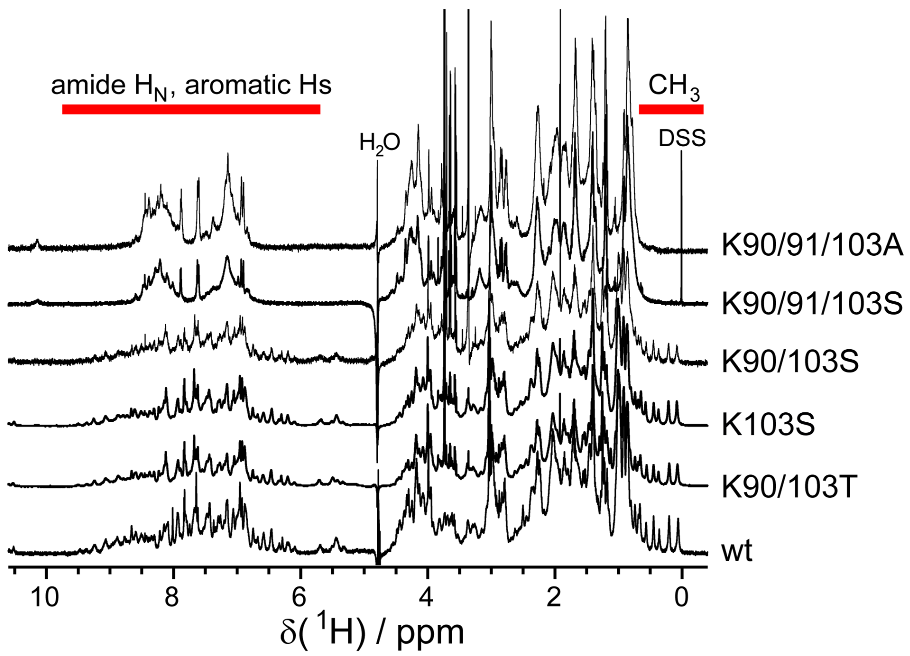

Supplementary Figure SI6: 1D ^{1}H NMR spectra of different Survivin120 mutants. The correct folding of Survivin120 mutants with substitutions of the amino acids linked to Tweezer binding was analyzed via NMR spectroscopy. Spectra were compared to that of Survivin120 wildtype. Correct folding was examined especially in the region of 6-10 ppm resulting from amides and aromatics and below 1 ppm obtained from methyl groups (highlighted in red). Mutant K90/103T was stable, folded correctly and hence chosen for further analysis.

Supplementary Table SI7: Thermodynamic data obtained from ITC titrations. ITC data from titrations with Survivin120 WT or Survivin120 K90/103T and TW, TW-ELTL, TW-ELTLGEFL and TW-LFEEGLLT was fitted to the *one set of sites* model with the Origin® software provided with the instrument. The thermodynamic data is listed in the table below.

	**Survivin120 WT**					**Survivin120 K90/103T**				
	KD[a] [µM]	N	ΔG [kcal]	ΔH [kcal]	-TΔS [kcal]	KD[a] [µM]	N	ΔG [kcal]	ΔH [kcal]	-TΔS [kcal]
**TW**	38 ± 4	20:1	-6.0	-1.3	-4.7	49 ± 5	34:1	5.9	-0.6	5.3
**TW-ELTL**	24 ± 4	2:1	-6.3	-2.5	-3.8	50 ± 10	1:1	5.9	-1.3	4.6
**TW-ELTLGEFL**	19 ± 3	2:1	-6.4	-1.9	-4.5	36 ± 10	1:1	6.0	-1.4	4.6
**TW-LFEEGLLT**	68 ± 22	4:1	-5.7	-1.0	-4.7	55 ± 24	1:1	5.9	-1.4	4.5

[a]Values reported are the mean ± s.e.m. of the fit. N, stoichiometry in tweezers per protein; G, Gibbs free energy; H, enthalpy; T, temperature; S, entropy.

Supplementary Figure SI8

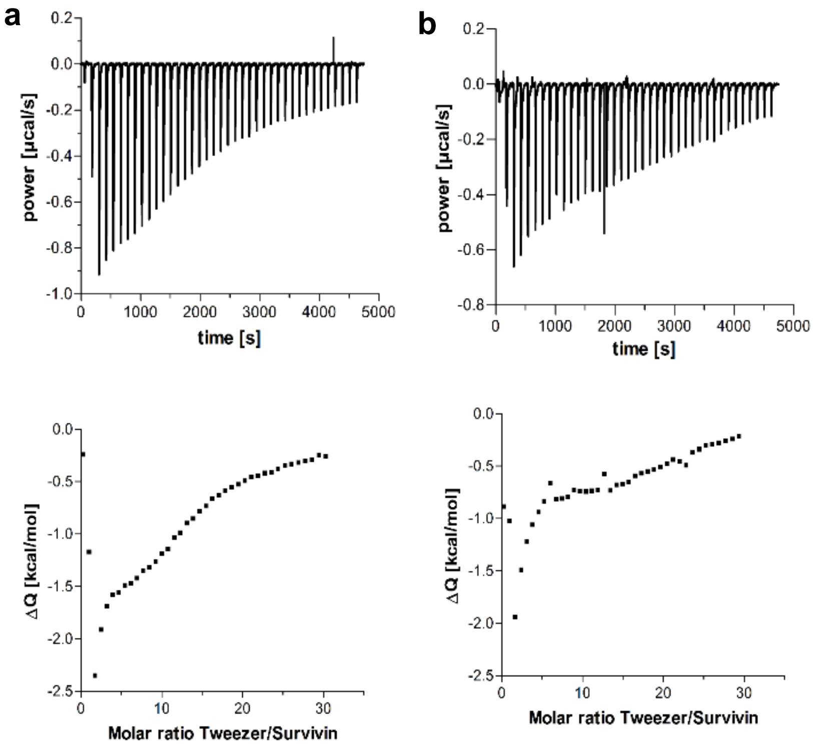

Supplementary Figure SI8: Reverse ITC titrations. Titration of 33.3 µM (a) and 34,4 µM (b) Survivin120 in the cell with 5 mM unmodified tweezer TW (a) and TW-ELTL (b) in the syringe. All titrations (n = 1) were performed in PBS, pH 7.4 at 25 °C. Top row: raw heating power over time subtracted by control heating power of Survivin120 titration into PBS. Bottom row: fit of the integrated energy values normalized to injected Tweezer.

Supplementary Figure SI9

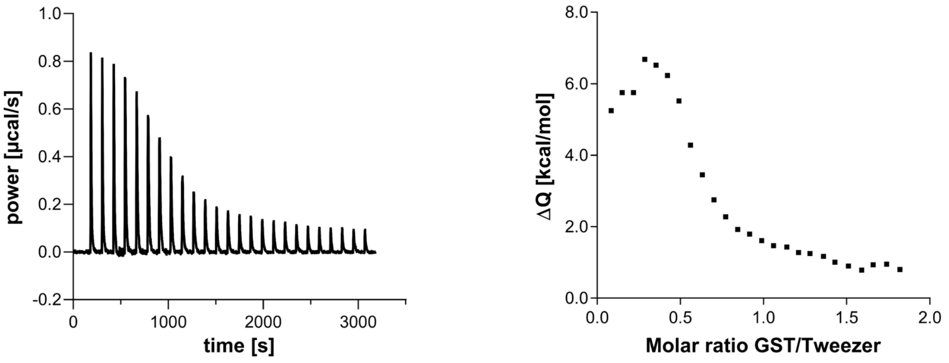

Supplementary Figure SI9: ITC titrations to control for GST binding to TW-ELTLGEFL. ITC titrations reveal an endothermic interaction between the GST anchor and the tweezer peptide conjugate. The left panel shows the peaks resulting from titration of 100 µM TW-ELTLGEFL in the cell with 914 µM GST in the syringe in PBS, pH 7.4 at 25 °C. The right panel shows the integrated energy values over molar ratio of GST and tweezer. The data (n = 1) revealed a biphasic thermogram, but was not fit due to the uncertainty of the exact binding mode of TW-ELTLGEFL binding to GST.

Supplementary Method SI10: Additional information, computational details

For the molecular dynamics (MD) simulations, the parameters for the unmodified tweezer TW were generated with the Swissparam server.[1] These parameters have been validated in previous works[2, 3, 4, 5] involving TW (also known as CLR01) and several biomolecules (see more details below). For the modified tweezers TW-ELTL and TW-ELTLGEFL, we applied the parameters established for TW in combination with the peptide fragments for which we used the standard protein force field parameters of CHARMM36m.[6] The parameters for the union between the peptide motifs and the tweezer were also obtained using the Swissparam server.

We used representative snapshots from the MD simulations as initial coordinates for the QM/MM optimizations (5 representative snapshots of each system, for a total of 20). To this end, cluster analyses of the MD trajectories were performed using the quality-threshold-based algorithm implemented for VMD,[7] with a RMSD cutoff of 3 Å. The snapshots are those closest to the center of the first most populated cluster, which corresponds to 35.95 % (K23), 49.11 % (K90) 22.87 % (K91) and 37.53 % (K103) of the total sampling in each case (see SI11). We note that the QM/MM energies resulting from the QM/MM optimizations correspond to zero Kelvin values, which do not distribute as those that can be sampled under a temperature regime and therefore a Boltzmann-like interpretation of the relative QM energy differences is not possible. We have observed in previous works[2, 3, 4] that relative QM energy differences span over a wide range that reaches tens of kcal/mol. Even so, the relative QM energy values allow assessing the relative stability, at electronic resolution, of an atomic configuration with respect to other.

As in previously reported works,[2, 3, 4] here our aim was the optimization of the geometry of tweezers complexes at the QM/MM level, e.g. using DFT to describe the whole tweezer structure (and part of the included amino acid), with an electrostatic embedding for the QM-MM treatment. The suitability of using the above-mentioned set of parameters for generating geometries for QM/MM optimizations is evidenced by our previous work on complexes of several tweezers with amino acids and short peptides.[2] In addition, QM/MM calculations in which the initial coordinates were obtained from MD simulations using the SwissParam parameters allowed us to successfully predict (with experimental agreement) binding sites in systems as diverse as 14-3-3 proteins, p97-Nterm, the N-term of a fragment of the first exon of a Huntingtin protein, and beta amyloid peptides, among others.[2, 4, 8, 9]

In previous published works, we also evaluated how well this set of parameters allows sampling the tweezer forming inclusion complexes in MD simulations.[4, 9, 10, 11] The average values of structural parameters measured from simulations of TW-Lys complexes are in agreement with a crystal structure reported for a TW-Lys complex in a 14-3-3 protein (PDB ID 5OEH).[2] This agreement is also observed in the current work (see SI13). Using these parameters, we

previously predicted that the formation of inclusion complexes of the tweezer with N17 (17-residue N-terminal fragment of the exon-1 domain of the Huntingtin protein) would result in the decrease of the α-helical content and amphipathic nature of N17, in agreement with REMD simulations (with Generalized Amber force field parameters for the tweezer) and experimental work.[8] Very recently, the SwissParam parameters were also used by us for simulating the formation of inclusion complexes of tweezers with model lipid bilayers and the effect of such tweezers on the membrane's structural properties, in excellent agreement with NMR measurements and biophysical experiments.[12] Nevertheless, we note that, although suitable for MD simulations and allowing a correct sampling of the geometry of tweezer complexes, such parameters should be used with caution as they may not be suitable for delivering properties such as accurate binding free energy estimations. Thus, we recently used CHARMM General Force Field (CGenFF) parameters to calculate the binding affinity in tweezer-Lys and tweezer-Arg complexes.[13]

For selection of the scrambled peptide, all possible permutations (3359) of the original peptide (ELTLGEFL) were generated. The FoldX program[14, 15] was used to evaluate the relative stability of the complexes between Survivin120 and these peptides bound to the NES region. The complexes of Survivin120 with the scrambled peptides were generated using as template a representative structure of the complex Survivin120 – TW-ELTLGEFL from our simulations. The scrambled peptide LFEEGLLT followed two criteria: 1) the leucine spacing that is part of the NES consensus sequence was disrupted. 2) it does not feature reverse sequences which would simply anchor the lysine from the other side.

Supplementary Table SI11: Most populated clusters from the 1:1 MD simulations of TW-ELTL forming complexes with K23, K90, K91 and K103 of Survivin120.

Cluster	K23	K90	K91	K103
	Population (%)			
	TW-ELTL/Survivin120			
**C1**	35.95	49.11	22.87	37.53
**C2**	26.41	19.33	14.23	24.51
**C3**	19.68	13.34	13.94	20.67

Supplementary Figure SI12

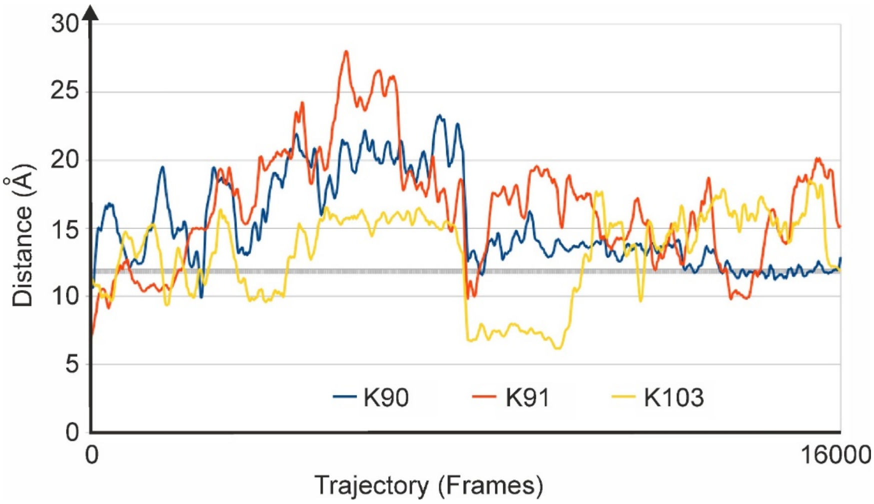

Supplementary Figure SI12: MD simulations of TW-ELTL encapsulating K103/K90/K91 of monomeric Survivin120. Distance between the centers of masses of the peptide tail of Tweezer-ELTL and the analogous segment (^{95}ELTL^{98}) of monomeric Survivin120 over 16000 frames collected every 5 ps along 80 ns of MD simulation (carried out with two independent simulations of 40 ns each). The gray bar located at a distance of 12 Å represents the cutoff above which no effective interaction occurs. The sudden decrease in the middle of the distance profiles corresponds to the values of the initial distances at the beginning of the second simulation.

Supplementary Table SI13: Structural parameters characterizing the inclusion complexes between the tweezers and lysine residues during the MD simulations. d(Cp-Cp') and d(Cm-Cm') are the distances between the terminal rings of the tweezer's arms. The angle a(C-C1-N) indicates how deep the respective lysine is threaded inside the tweezer's cavity. d(P1-N) is the distance from the phosphate group bound to C1 and the nitrogen atom of the indicated lysine. The experimental values are taken from the crystal structure with PDB-ID 5OEH [https://www.rcsb.org/structure/5OEH] of a TW-lysine complex in a 14-3-3 protein.[2] All complexes were conserved during the simulations.

	**TW**	**TW-ELTL**
**d(Cp-Cp')**	values in angstroms [Å]	
**K103**	6.5 ± 1.0*	5.5 ± 0.4
**K91**	5.8 ± 0.7	5.7 ± 0.5
**K90**	5.8 ± 0.4	5.8 ± 0.7
**K23**	5.7 ± 0.5	5.7 ± 0.4
**Exp. Value**	5.5	
**d(Cm-Cm')**	values in angstroms [Å]	
**K103**	5.2 ± 1.1*	4.0 ± 0.4
**K91**	4.4 ± 0.8	4.2 ± 0.5
**K90**	4.2 ± 0.4	4.3 ± 0.8
**K23**	4.1 ± 0.5	4.1 ± 0.5
**Exp. Value**	3.7	
**a(C-C1-N)**	values in degrees [°]	
**K103**	84.0 ± 5.3	81.0 ± 6.5
**K91**	88.9 ± 6.3	87.5 ± 6.2
**K90**	94.5 ± 6.1	83.9 ± 5.7
**K23**	86.0 ± 5.9	84.9 ± 5.5
**Exp. Value**	90.3	
**d(P1-N)**	values in angstroms [Å]	
**K103**	4.0 ± 0.2	4.6 ± 1.0**
**K91**	4.0 ± 0.1	3.9 ± 0.1
**K90**	4.0 ± 0.2	4.0 ± 0.1
**K23**	4.0 ± 0.2	3.9 ± 0.1
**Exp. Value**	4.1	

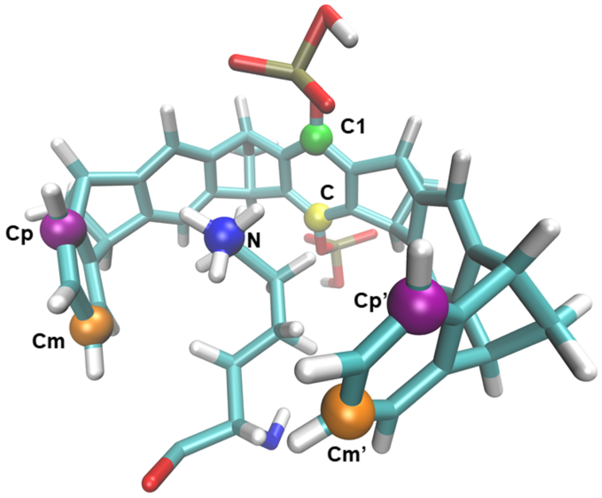

*During the simulations, the tweezer performs a partial rotation around the lysine, which is favored by an opening of its arms. **The deviation for the d(P1-N) distance in K103 is due to the rotation of the phosphate group.

Supplementary Figure SI14

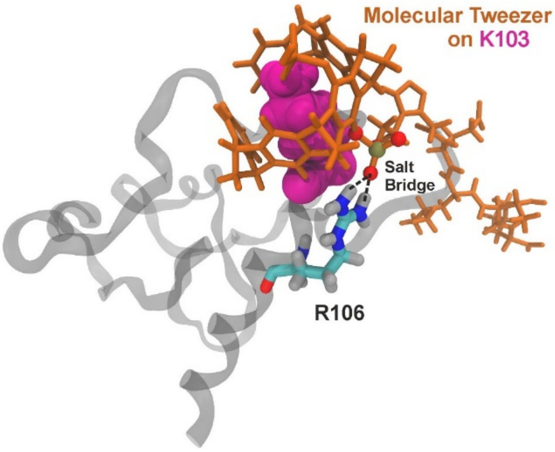

Supplementary Figure SI14: Salt bridge between the phosphate group of TW-ELTL on K103 and R106. The tweezer cavity (orange) encapsulates K103 (pink), while the peptide moiety contacts the NES on the surface of Survivin120. An additional salt bridge between the phosphate group of the tweezer and R106 stabilizes TW-ELTL on the protein.

Supplementary Figure SI15

a

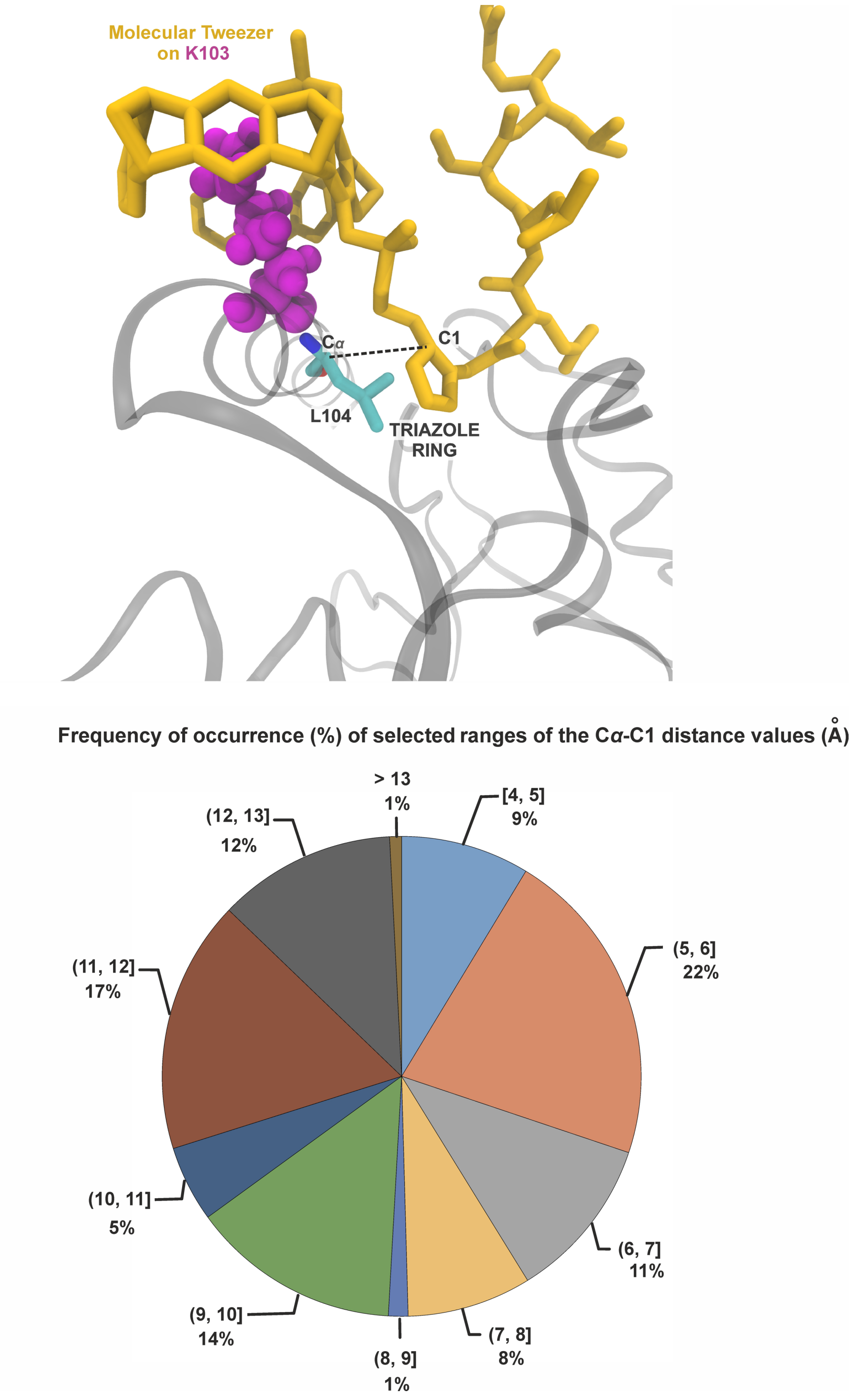

Supplementary Figure SI15: Profile of the distance C*α*-C1 as indicator of the proximity of the triazole group to the backbone of Survivin. a) Distance between the *α* carbon of L104 (C*α*) and the carbon C1 of the triazole ring. b) Frequency of certain values of the C*α*-C1 distance. For 30 % of the simulation the values of the C*α*-C1 distance are below 6 Å. Furthermore, for 50 % of the sampling time, the values of the Cα-C1 distance are below 8 Å.

Supplementary Figure SI16

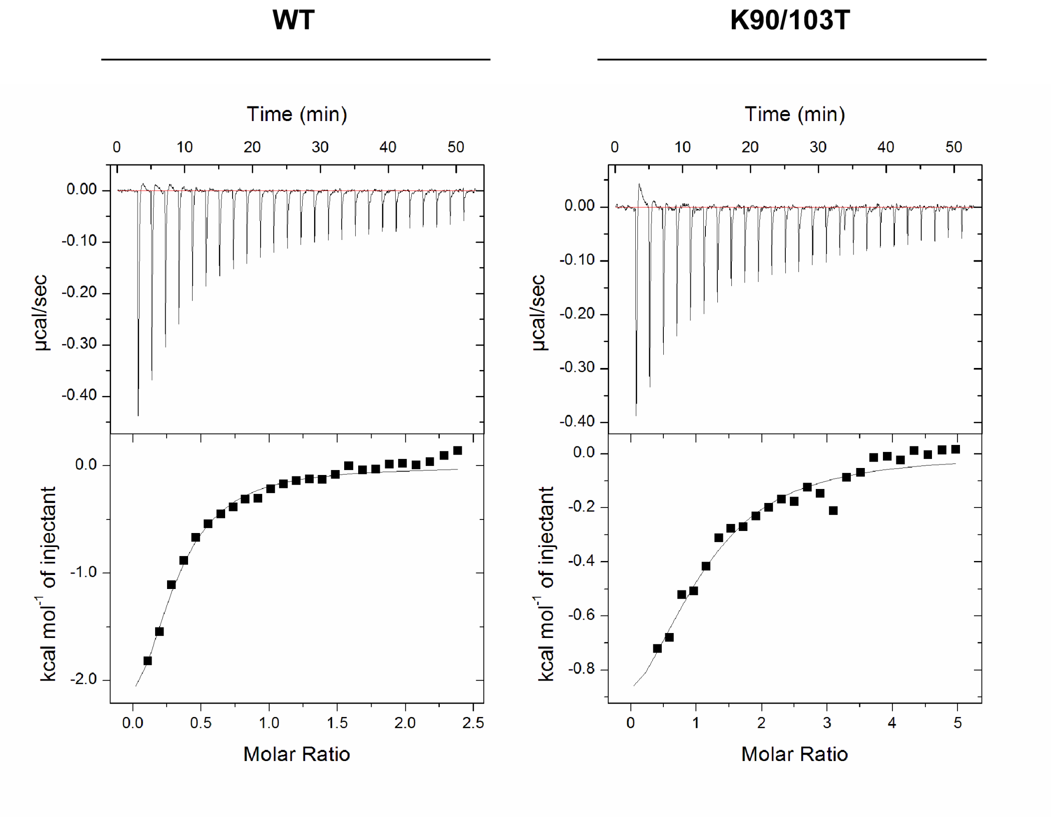

Supplementary Figure SI16: ITC titrations of TW-LFEEGLLT binding to Survivin120 WT and K90/103T. ITC titrations reveal an exothermic interaction between the scrambled peptide-modified tweezer (TW-LFEEGLLT) and Survivin120 WT as well as K90/103T. Titration of TW-LFEEGLLT (0.1 µM) in the cell with 1.2 mM Survivin120 WT (left) or 2 mM Survivin120 K90/103T (right) in the syringe in PBS, pH 7.4 at 25 °C. The solid line in the bottom panel represents the best fit with a *one set of sites* model. The dissociation constants are 69 ± 23 µM for Survivin120 WT and 55 ± 26 µM for Survivin120 K90/103T. Graphs represent one representative example each from three independent experiments (n = 3).

Supplementary Figure SI17

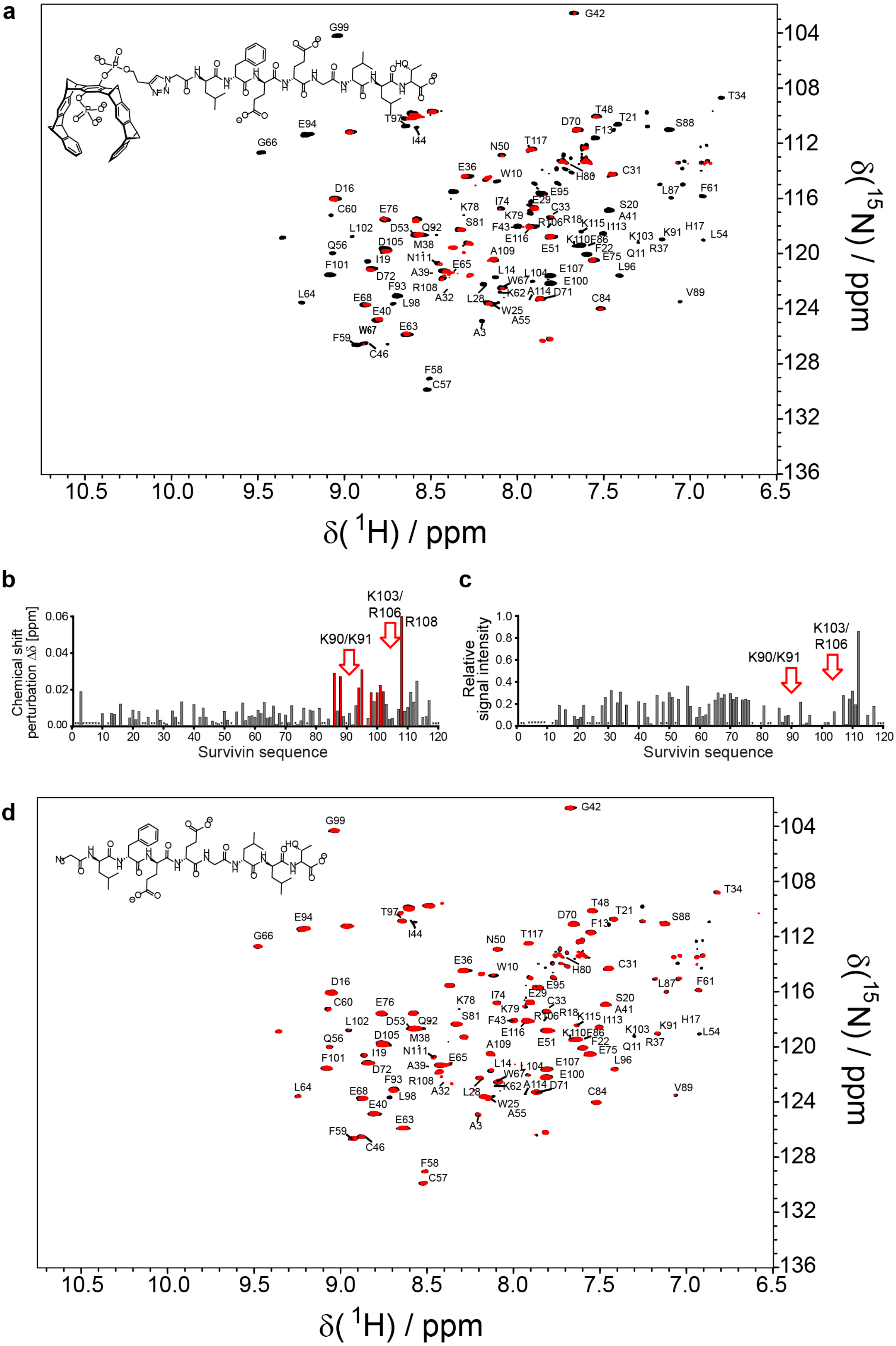

Supplementary Figure SI17: ^{15}N-HSQC NMR spectra of ^{15}N-Survivin120 in the absence (black) and presence (red) of TW-LFEEGLLT. a) ^{15}N-HSQC NMR spectra of ^{15}N-Survivin120 in the absence (black) and presence (red) of the scrambled-peptide tweezer TW-LFEEGLLT. Assigned signals are labeled. A shift or reduction of signal intensity is indicative of binding. Chemical shift perturbation (b) and relative signal intensities (c) plotted against the Survivin120 sequence. Residues displaying above-average chemical shift perturbation are highlighted in red. The tweezer with the scrambled peptide sequence can still bind the two sites K90/K91 and K103/R106 like the unmodified TW. Slight chemical shift perturbations due to the spatial proximity of the peptide moiety to the anchoring residue are expected, but no large perturbations like for TW-ELTL or TW-ELTLGEFL are observed in between residues 91-103, indicating that the peptide moiety does not form specific contacts with Survivin. d) ^{15}N-HSQC NMR spectra of ^{15}N-Survivin120 in the absence (black) and presence (1:1, red) of the scrambled peptide LFEEGLLT. Higher peptide concentrations could not be reached due to the poor solubility of the peptide. No signal shifts are observed showing no binding of the peptide to Survivin. Slightly lower signal intensities are due to dilution of the protein sample upon addition of the peptide.

Supplementary Figure SI18

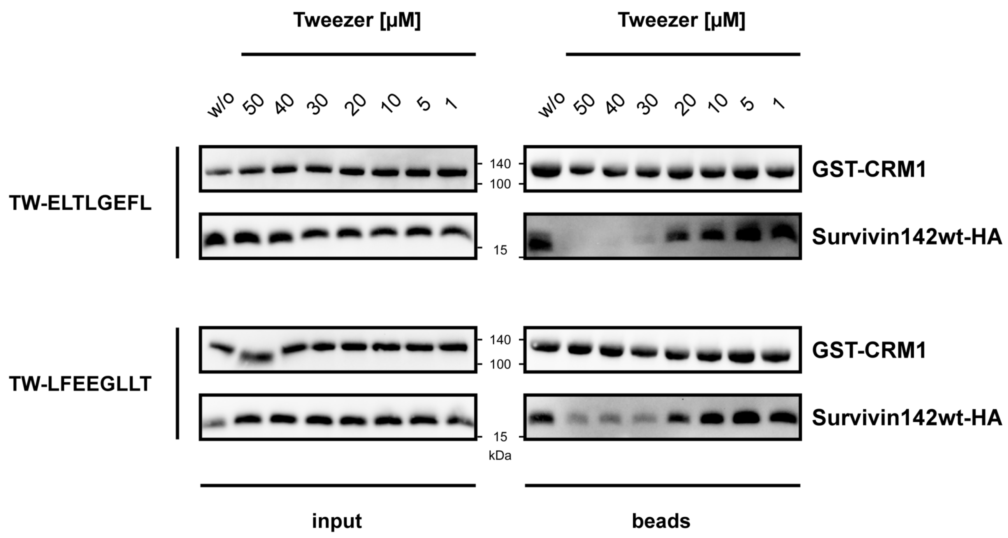

Supplementary Figure SI18: Pull-down assays with concentrations ranging between 1 and 50 µM of TW-ELTLGEFL and the scrambled peptide-modified tweezer (TW-LFEEGLLT). 293T cells were transfected with Survivin142-HA. Respective cell lysates were pre-incubated with TW-ELTLGEFL (top) or scrambled peptide TW-LFEEGLLT (bottom) at different concentrations between 1 and 50 µM. GST-CRM1 bait protein was mixed with pre-incubated lysates, recombinant RanQ69L and dGTP to enable complex assembly. Protein complexes were pulled by GSH-coated beads. Proteins in input and beads samples were analyzed via immunoblotting with antibodies specific for GST and HA. TW-ELTLGEFL inhibited the Survivin/CRM1 interaction efficiently at concentrations of 20-30 µM, whereas the scrambled peptide tweezer did not reach a comparable effect even at 50 µM. One representative example of 2 independent experiments performed in 3 technical replicates is shown. Samples derive from the same experiment and gels/blots were processed in parallel. Source data are provided as a Source Data file.

Supplementary Figure SI19

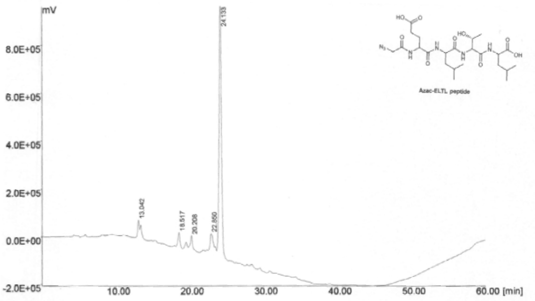

File name : HW_pep90001.CH1

Info :
EC 250/4 Nucleosil 100-3 C18
Flow 0.5 mL Wasser/0.1% TFA:Acetonitril Gradient
Range 0.001 UV 214 nm

Vial # = 1 Rack # = 1
Injection Date :21-Jun-2016 10:54:24
Curr. Date : 25-Jun-2020 10:19:40
User : WOELL
Group : SODOM
Control Method :TEST

#	Name	RT	Area[mV.Sec]	%Area
1		13.042	507908	2.13
2		18.517	525054	2.20
3		20.208	569824	2.38
4		22.850	717423	3.00
5		24.133	21572011	90.29

Total Area of Peak = 23892219 [mV.Sec]
Injection Volume = 20.00 ul

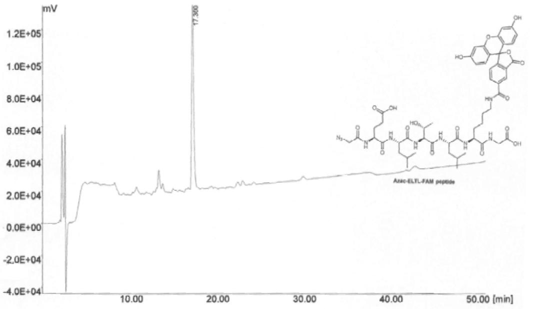

File name : HW_pep123_2a001.CH1

Info :
EC 250/4 Luna 5µm C18(2) 100 A
Flow 1.0 mL Wasser:Acetonitril Gradient
Range 0.001 UV 220 nm
erneut lyophilisiert

Vial # = 1 Rack # = 1
Injection Date :20-Feb-2019 10:05:54
Curr. Date : 25-Jun-2020 10:51:48
User : WOELL
Group : SODOM
Control Method :INESA

#	Name	RT	Area[mV.Sec]	%Area
1		17.300	1657619	100.00

Total Area of Peak = 1657619 [mV.Sec]
Injection Volume = 20.00 ul

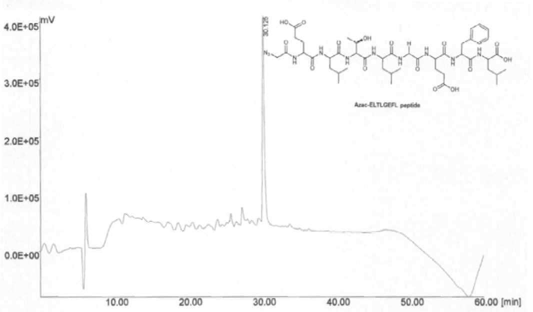

File name : HW_pep101_3001.CH1

Info :
EC 250/4 Luna 5µm C18(2) 100 A
Flow 0.5 mL Wasser:Acetonitril Gradient
Range 0.001 UV 214 nm

Vial # = 1 Rack # = 1
Injection Date :27-May-2020 11:43:26
Curr. Date : 27-May-2020 13:55:42
User : WOELL
Group : SODOM
Control Method :TEST

#	Name	RT	Area[mV.Sec]	%Area
1		30.125	5900122	100.00

Total Area of Peak = 5900122 [mV.Sec]
Injection Volume = 20.00 ul

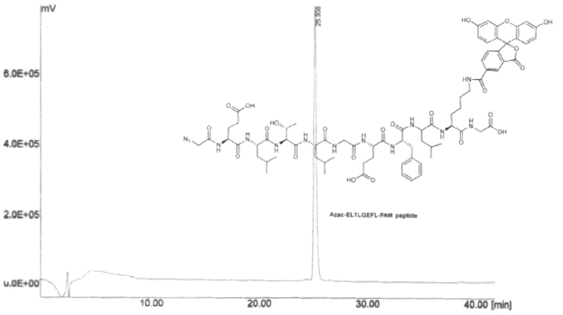

File name : IS_HW_124_F42001.CH1

Info :
EC 250/4 Luna 5µm C18(2) 100 A
Flow 1.0 mL Wasser/0.1% TFA:Acetonitril Gradient
Range 0.001 UV 220 nm

Vial # = 1 Rack # = 1
Injection Date : 5-Nov-2018 16:19:26
Curr. Date : 25-Jun-2020 10:32:50
User : WOELL
Group : SODOM
Control Method :INESA

#	Name	RT	Area[mV.Sec]	%Area
1		25.308	12248826	100.00

Total Area of Peak = 12248826 [mV.Sec]
Injection Volume = 20.00 ul

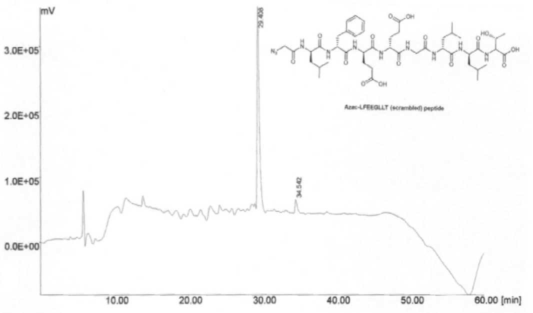

File name : HW_pep136001.CH1

Info :
EC 250/4 Luna 5µm C18(2) 100 A
Flow 0.5 mL Wasser:Acetonitril Gradient
Range 0.001 UV 214 nm

Vial # = 1 Rack # = 1
Injection Date :27-May-2020 10:41:20
Curr. Date : 25-Jun-2020 10:34:20
User : WOELL
Group : SODOM
Control Method :TEST

#	Name	RT	Area[mV.Sec]	%Area
1		29.408	5848665	93.93
2		34.542	378279	6.07

Total Area of Peak = 6226943 [mV.Sec]
Injection Volume = 20.00 ul

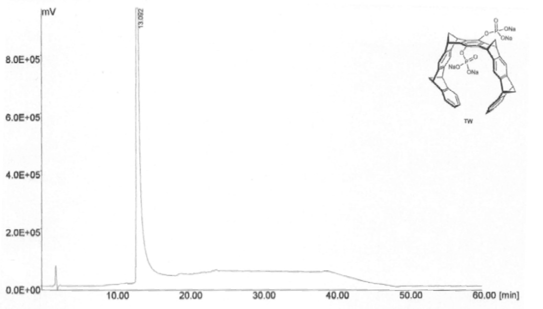

File name : CLR01_042041001.CH1

Info :
EC 250/4 Luna 5µm C18(2) 100 A
Flow 1.0 mL Wasser:Acetonitril Gradient
Range 0.001 UV 220 nm

Vial # = 1 Rack # = 1
Injection Date :28-May-2020 12:02:16
Curr. Date : 25-Jun-2020 10:42:48
User : WOELL
Group : SODOM
Control Method :PHILIPP2

#	Name	RT	Area[mV.Sec]	%Area
1		13.092	37583904	100.00

Total Area of Peak = 37583904 [mV.Sec]
Injection Volume = 20.00 ul

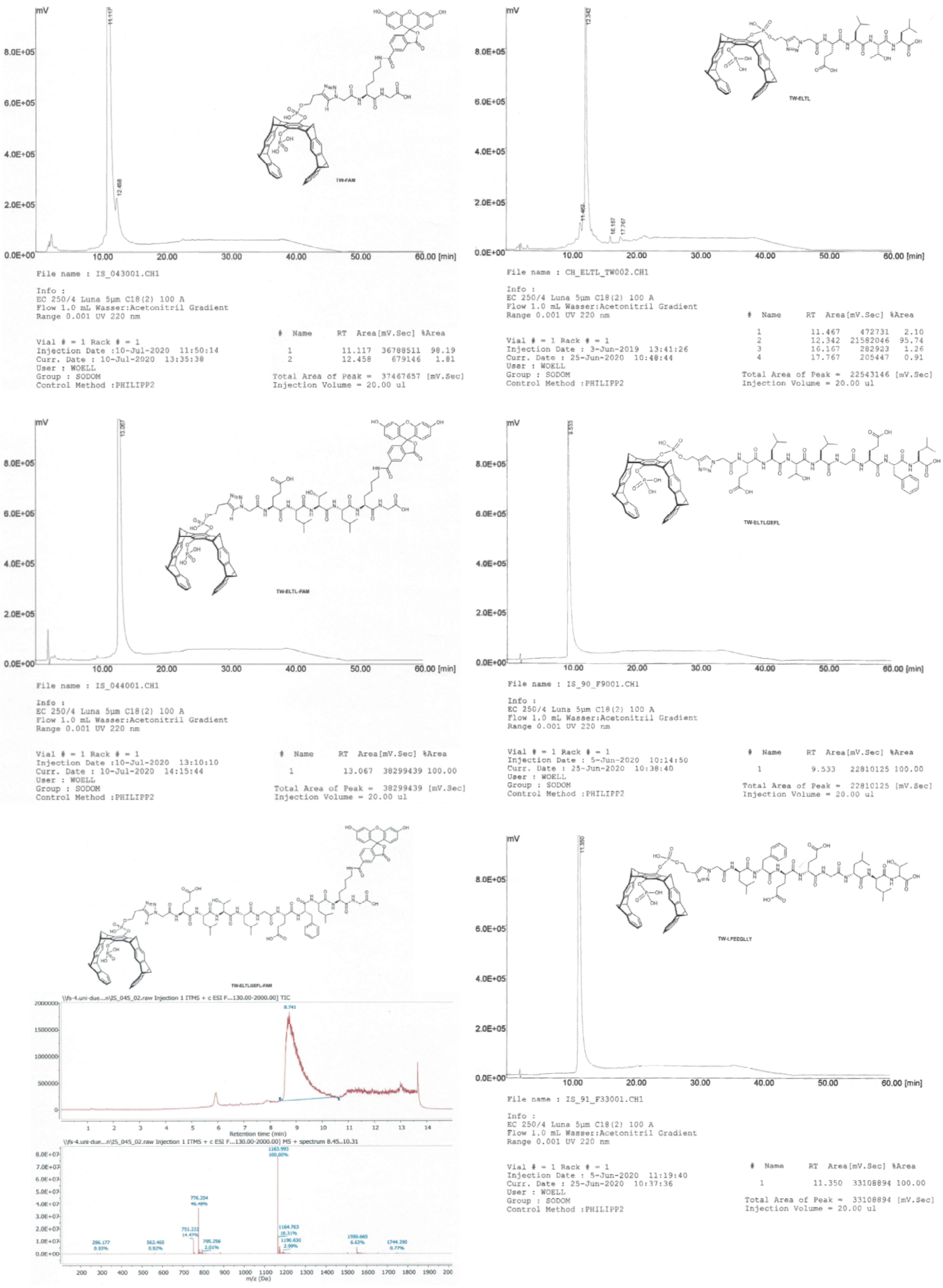

Supplementary Figure SI19: LC traces of the final products (peptides, FAM-labeled peptides, tweezer molecules). Peptides: Azac-ELTL, Azac-ELTL-FAM, Azac-ELTLGEFL, Azac-ELTLGEFL-FAM, Azac-LFEEGLLT (scrambled peptide). Tweezer molecules: unmodified tweezer TW, TW-FAM, TW-ELTL, TW-ELTL-FAM, TW-ELTLGEFL, TW-ELTLGEFL-FAM, TW-LFEEGLLT (from top to bottom, left to right). The LC trace of TW-ELTLGEFL-FAM differs from all other traces, because it was measured on a different stationary phase.

Supplementary Table SI20: List of plasmids and primers

Plasmid	Template	Reference	Primer	Sequence (5' → 3')
**Prokaryotic expression constructs**				
pET41-GST-PreSc-Crm1	pC3-Crm1-HA	[16]	ApaI_Crm1-1_f	AAA GGG CCC ATG CCA GCA ATT ATG
			Bam_Crm1-1071_r	TTT GGA TCC TTA ATC ACA CAT TTC TTC TGG
pET41-GST-PreSc-RanQ69L	pGEX-RanQ69L	[16]	ApaI_RanQL_fw	AAA GGG CCC ATG GCT GCG CAG GGA G
			BamHI_RanQL_rev	TTT GGA TCC TTA CAG GTC ATC ATC CTC ATC CGG
pET41-GST-PreSc-Survivin120WT	pc3-Survivin-GFP	[16]	Apa-Surv_for	AAA GGG CCC GGT GCC CCG ACG TTG CCC
			Bam-Surv120_rev	TTT GGA TCC TTA CTT ATT GTT GGT TTC CTT TGC
pET41-GST-PreSc-Survivin120 K90/91/103A	pET41-GST-PreSc-Survivin120WT	This work.	Q5SDM_SurvK9091103A_F	ACC CTT GGT GAA TTT TTG GCG CTG GAC AGA GAA AGA GCC
			Q5SDM_SurvK9091103A_R	TAA TTC TTC AAA CTG CGC CGC GAC AGA AAG GAA AGC GCA
pET41-GST-PreSc-Survivin120 K90S	pET41-GST-PreSc-Survivin120WT	This work.	Surv_K90S_fw	CTT TCT GTC TCG AAG CAG TTT
			Surv_K90S_rev	TTC AAA CTG CTT GCT GAC AGA
pET41-GST-PreSc-Survivin120 K90/103S	pET41-GST-PreSc-Survivin120K90S	This work.	Q5SDM_SurvK103S_F	TGA ATT TTT GAG CCT GGA CAG AGA AAG AG
			Q5SDM_SurvK103S_R	CCA AGG GTT AAT TCT TCA AAC
pET41-GST-PreSc-Survivin120 K103S	pET41-GST-PreSc-Survivin120WT	This work.	Q5SDM_SurvK103S_F	TGA ATT TTT GAG CCT GGA CAG AGA AAG AG
			Q5SDM_SurvK103S_R	CCA AGG GTT AAT TCT TCA AAC
pET41-GST-PreSc-Survivin120 K90/91/103S	pET41-GST-PreSc-Survivin120 K103S	This work.	Q5SDM_SurvK9091103S_F	ACC CTT GGT GAA TTT TTG AGC CTG GAC AGA GAA AGA GCC
			Q5SDM_SurvK9091103S_R	TAA TTC TTC AAA CTG GCT GCT GAC AGA AAG GAA AGC GCA
pET41-GST-PreSc-Survivin120 K103T	pET41-GST-PreSc-Survivin120WT	This work.	Q5SDM_SurvK103T_F	GAA TTT TTG ACG CTG GAC AGA GAA AGA GC
			Q5SDM_SurvK103T_R	ACC AAG GGT TAA TTC TTC
pET41-GST-PreSc-Survivin120 K90/103T	pET41-GST-PreSc-Survivin120K103T	This work.	Q5SDM_SurvK90T_F	CCT TTC TGT CAC GAA GCA GTT TGA AG
			Q5SDM_SurvK90T_R	AAA GCG CAA CCG GAC GAA
pGEX-GST-PreScission protease	-	[17]	-	-
**Eukaryotic expression constructs**				
pc3-Survivin142-HA	pc3-Survivin-GFP	[16]	Surv-Bam for	AAAGGATCCACATGGGTGCCCCGACGTTG
			Surv-Nhe rev	TTTGCTAGCATCCATGGCAGCCAGCTGC

Supplementary References

1. Zoete V, Cuendet MA, Grosdidier A, Michielin O. SwissParam: A fast force field generation tool for small organic molecules. *Journal of Computational Chemistry* **32**, 2359-2368 (2011).

2. Bier D*, et al.* Molecular tweezers modulate 14-3-3 protein–protein interactions. *Nature chemistry* **5**, 234-239 (2013).

3. Lump E*, et al.* A molecular tweezer antagonizes seminal amyloids and HIV infection. *Elife* **4**, (2015).

4. Trusch F*, et al.* Molecular tweezers target a protein–protein interface and thereby modulate complex formation. *Chemical Communications* **52**, 14141-14144 (2016).

5. Bier D*, et al.* The Molecular Tweezer CLR01 Stabilizes a Disordered Protein-Protein Interface. *J Am Chem Soc* **139**, 16256-16263 (2017).

6. Huang J*, et al.* CHARMM36m: an improved force field for folded and intrinsically disordered proteins. *Nat Methods* **14**, 71-73 (2017).

7. Humphrey W, Dalke A, Schulten K. VMD - Visual Molecular Dynamics. *J Molec Graphics* **14**, 33-38 (1996).

8. Vöpel T*, et al.* Inhibition of Huntingtin Exon-1 Aggregation by the Molecular Tweezer CLR01. *Journal of the American Chemical Society* **139**, 5640-5643 (2017).

9. Mittal S, Bravo-Rodriguez K, Sanchez-Garcia E. Mechanism of Inhibition of Beta Amyloid Toxicity by Supramolecular Tweezers. *The Journal of Physical Chemistry B* **122**, 4196-4205 (2018).

10. Lopes DH*, et al.* Molecular tweezers inhibit islet amyloid polypeptide assembly and toxicity by a new mechanism. *ACS Chem Biol* **10**, 1555-1569 (2015).

11. Dutt S*, et al.* Molecular Tweezers with Varying Anions: A Comparative Study. *The Journal of Organic Chemistry* **78**, 6721-6734 (2013).

12. Weil T*, et al.* Supramolecular Mechanism of Viral Envelope Disruption by Molecular Tweezers. *Journal of the American Chemical Society* **142**, 17024-17038 (2020).

13. Ruiz-Blanco YB, Sanchez-Garcia E. CL-FEP: An End-State Free Energy Perturbation Approach. *Journal of Chemical Theory and Computation* **16**, 1396-1410 (2020).s

14. Delgado J, Radusky LG, Cianferoni D, Serrano L. FoldX 5.0: working with RNA, small molecules and a new graphical interface. *Bioinformatics* **35**, 4168-4169 (2019).

15. Buß O, Rudat J, Ochsenreither K. FoldX as Protein Engineering Tool: Better Than Random Based Approaches? *Computational and Structural Biotechnology Journal* **16**, 25-33 (2018).

16. Knauer SK, Bier C, Habtemichael N, Stauber RH. The Survivin-Crm1 interaction is essential for chromosomal passenger complex localization and function. *EMBO Rep* **7**, 1259-1265 (2006).

17. van den Boom J, Trusch F, Hoppstock L, Beuck C, Bayer P. Structural Characterization of the Loop at the Alpha-Subunit C-Terminus of the Mixed Lineage Leukemia Protein Activating Protease Taspase1. *PLoS One* **11**, e0151431 (2016).
