# Supplementary material for: Specific inhibition of the Survivin–CRM1 interaction by peptide-modified molecular tweezers
Source: Nat Commun. 2021 Mar 8;12:1505. doi: 10.1038/s41467-021-21753-9 (PMC7940618; doi:10.1038/s41467-021-21753-9)
Supplement: Supplementary file 2 — Reporting Summary [file 41467_2021_21753_MOESM2_ESM.pdf]

nature research

Corresponding author(s): Elsa Sanchez-Garcia, Thomas Schrader, Shirley Knauer

Last updated by author(s): Jan 25, 2021

Reporting Summary

Nature Research wishes to improve the reproducibility of the work that we publish. This form provides structure for consistency and transparency in reporting. For further information on Nature Research policies, see Authors & Referees and the Editorial Policy Checklist .

Statistics

For all statistical analyses, confirm that the following items are present in the figure legend, table legend, main text, or Methods section.

n/a	Confirmed	
☐	☒	The exact sample size (*n*) for each experimental group/condition, given as a discrete number and unit of measurement
☐	☒	A statement on whether measurements were taken from distinct samples or whether the same sample was measured repeatedly
☒	☐	The statistical test(s) used AND whether they are one- or two-sided<br>*Only common tests should be described solely by name; describe more complex techniques in the Methods section.*
☒	☐	A description of all covariates tested
☒	☐	A description of any assumptions or corrections, such as tests of normality and adjustment for multiple comparisons
☐	☒	A full description of the statistical parameters including central tendency (e.g. means) or other basic estimates (e.g. regression coefficient) AND variation (e.g. standard deviation) or associated estimates of uncertainty (e.g. confidence intervals)
☒	☐	For null hypothesis testing, the test statistic (e.g. *F*, *t*, *r*) with confidence intervals, effect sizes, degrees of freedom and *P* value noted<br>*Give P values as exact values whenever suitable.*
☒	☐	For Bayesian analysis, information on the choice of priors and Markov chain Monte Carlo settings
☒	☐	For hierarchical and complex designs, identification of the appropriate level for tests and full reporting of outcomes
☒	☐	Estimates of effect sizes (e.g. Cohen's *d*, Pearson's *r*), indicating how they were calculated

Our web collection on statistics for biologists contains articles on many of the points above.

Software and code

Policy information about availability of computer code

Data collection	ITC experiments: The MicroCal iTC200 software (version 1.26.1) from GE Healthcare/ Malvern Panalytical supplied with the instrument was used for ITC data collection.<br>Fluorescence anisotropy measurements: The software Spectra Manager™ II from Jasco supplied with the instrument was used.<br>NMR experiments: NMR data were collected using Topspin 3.5 (Bruker) using the NMRlib 2.0 pulse sequence tools library from IBS (Grenoble, France) available at http://www.ibs.fr/research/scientific-output/software/pulse-sequence-tools/ .<br>MD simulations: All simulations were carried out with Gromacs 4.6.7 using the Amber ff99SB force field extended with the ZAFF forcefield and NAMD 2.12; topologies were created with Modeller 9.17, the TLEaP module of Amber 12.21 and ACPYPE 2013.<br>QM/MM: Software ChemShell 3.5.0, DL-FIND geometry optimizer, Turbomole V6.4, DL_Poly_2.
Data analysis	ITC experiments: ITC thermograms were fitted to a one set of sites model with the software MicroCal Analysis Launcher in Origin provided with the instrument (Origin 7; version 7.0552). Heat of dilution was subtracted as constant from each data point.<br>Fluorescence anisotropy measurements: Data was plotted with GraphPad Prism 8 (version 8.4.1).<br>NMR experiments: Spectra were processed with Topspin 3.5 (Bruker) and analyzed in CARA (version 1.9.1.7; http://cara.nmr.ch). Chemical shift perturbation and relative signal intensities were calculated from the raw chemical shift data and peak intensities using Excel 2016 (Microsoft) and plotted with GraphPad Prism 5.0.<br>MD simulations: PROCHECK v.3.5.4, Gromacs 4.6.7, R 3.3.1, R package cluster 2.0.6, R package fpc 2.1.10, Python 3.5.3, PyMOL 1.8.6 compiled for Python 2.7.13., VMD 1.9.4, PyContact 1.0.1.<br>Western blot: Western blots were analyzed by densitometric analysis with ImageJ version 1.52p (National Institutes of Health).

For manuscripts utilizing custom algorithms or software that are central to the research but not yet described in published literature, software must be made available to editors/reviewers. We strongly encourage code deposition in a community repository (e.g. GitHub). See the Nature Research guidelines for submitting code & software for further information.

Data

Policy information about availability of data

All manuscripts must include a data availability statement. This statement should provide the following information, where applicable:

- Accession codes, unique identifiers, or web links for publicly available datasets
- A list of figures that have associated raw data
- A description of any restrictions on data availability

The authors declare that all data supporting the findings of this study are available within the article and its supplementary information and source data files. Additionally, large datasets generated during MD or GaMD simulations and QM/MM calculations are available from the corresponding author on reasonable request. The raw data associated with Figures 3, 4, 5 b and c, 6 and 7 as well as table 1 and SI18 are provided as Source Data file.
Accession codes used in the study are publicly available as Uniprot protein sequence O15392-1 [https://www.uniprot.org/uniprot/O15392] (human BIRC5 isoform alpha) and PDB entries 1E31 [https://www.rcsb.org/structure/1E31], 1F3H [https://www.rcsb.org/structure/1F3H], 3UEG [https://www.rcsb.org/structure/3UEG], 3UEH [https://www.rcsb.org/structure/3UEH], 3UEI [https://www.rcsb.org/structure/3UEI], 1XOX [https://www.rcsb.org/structure/1XOX].

Field-specific reporting

Please select the one below that is the best fit for your research. If you are not sure, read the appropriate sections before making your selection.

☒ Life sciences ☐ Behavioural & social sciences ☐ Ecological, evolutionary & environmental sciences

For a reference copy of the document with all sections, see nature.com/documents/nr-reporting-summary-flat.pdf

Life sciences study design

All studies must disclose on these points even when the disclosure is negative.

Sample size	Measures taken to verify reproducibility included to perform every experiment at least three times (for exeptions, see below). Additionally, the number of repetitions for all experiments is reported in the respective figure legend or methods section. Although no statistical analysis was performed to predetermine sample size, but three biological replicates is considered standard practice for most biochemical assays.
Data exclusions	As stated in the source data file, only in one experiment two data points were not recorded due to technical issues with the machine (Fig. 7b, flourescence anisotropy measurement No. 1). As also revealed in the source data file, no other data was excluded from the analyses. However, experiments that have undoubtedly not been run correctly due to e.g. technical issues were not included in the study (e.g. when a protein precipitates prior to measurements, or there are no detectable bands at all in Western Blot analyses).
Replication	Measures taken to verify reproducibility included to perform every experiment at least three times. However, as we had to handle large proteins including mutants that could not be easily expressed, replication of the respective experiment was not possible due to very low protein concentrations, as already stated in the manuscript: Low protein yields of the mutant CRM1_1-1062VLV430AAA did not allow replicates of the respective experiment. After initial establishment and technical optimization of each method, all attempts at replication were successful.
Randomization	The samples were not randomized in our study (not applicable here). Here, the main task was to establish a concentration-dependency of chemical inhibitors, so it was not possible to rearrange sample orders randomly.
Blinding	Blinding was not used in our study (not applicable here). More specifically, regarding the elaborate chemical syntheses and protein expression protocols, combined with the requirement of minimized storage duration of ligands and protein batches for an optimal experimental performance, blinding was virtually not possible throughout the complete study and therefore not applied even to single experimental setups.

Reporting for specific materials, systems and methods

We require information from authors about some types of materials, experimental systems and methods used in many studies. Here, indicate whether each material, system or method listed is relevant to your study. If you are not sure if a list item applies to your research, read the appropriate section before selecting a response.

Materials & experimental systems

n/a	Involved in the study	
☐	☒	Antibodies
☐	☒	Eukaryotic cell lines
☒	☐	Palaeontology
☒	☐	Animals and other organisms
☒	☐	Human research participants
☒	☐	Clinical data

Methods

n/a	Involved in the study	
☒	☐	ChIP-seq
☒	☐	Flow cytometry
☒	☐	MRI-based neuroimaging

Antibodies

Antibodies used	primary antibodies:<br>anti-Crm1, rabbit polyclonal, Novus Biologicals Ltd., Cambridge (NB100-79802, RRID:AB_2215823), 1:10,000 (WB)<br>anti-GST, mouse monoclonal, Santa Cruz Biotechnology Inc., Heidelberg (sc-57753, RRID:AB_783587), 1:1,000 (WB)<br>anti-Ran, rabbit polyclonal, Novus Biologicals Ltd., Cambridge (NBP1-31776), 1:1,000 (WB)<br>anti-HA, mouse monoclonal, BioLegend, Covance (MMS-101R, RRID:AB_29126), 1:1,000 (WB)<br>secondary antibodies:<br>anti-rabbit IgG-horseradish peroxidase (HRP), donkey, GE Healthcare Life Sciences, Freiburg (NA934, RRID:AB_772206), 1:10,000 (WB)<br>anti-mouse IgG-horseradish peroxidase (HRP), sheep, GE Healthcare Life Sciences, Freiburg (NXA931, RRID:AB_772209), 1:10,000 (WB)
Validation	All antibodies used in this study are used in our laboratory for many years. Also the lots used in the current study were successfully used in other projects in the lab before. In addition, we validated all antibodies in combination with already validated recombinant proteins (Crm1, GST, Ran) or eukaryotic cell lysates overexpressing the respective validated plasmids (Crm1, HA) in Western Blot (WB) analyses, which already serves as validation to exclude unspecific binding or cross-reactivity.<br>Of note, all our antibody suppliers (Abcam, Novus, Santa Cruz and GE Healthcare) have very strict validation procedures including e.g. knockdown validation.<br>Validation of primary antibodies according to the manufacturer's website:<br>anti-Crm1 (Novus Biologicals Ltd., NB100-79802) has been validated for use in western blotting to detect human CRM1, as stated on the suppliers product page.<br>anti-GST (Santa Cruz Biotechnology Inc., sc-57753) has been validated for use in western blotting to detect recombinant GST protein, as stated on the suppliers product page.<br>anti-Ran (Novus Biologicals Ltd., Cambridge, NBP1-31776) has been validated for use in western blotting to detect human Ran, as stated on the suppliers product page.<br>anti-HA (Abcam PLC, AB9110) has been validated for use in western blotting to detect the HA epitope tag, as stated on the suppliers product page.

Eukaryotic cell lines

Policy information about cell lines

Cell line source(s)	293T/17 [HEK 293T/17] ATCC® CRL-11268™ - clone 17 selected specifically for its high transfectability.
Authentication	Morphology check by microscope.
Mycoplasma contamination	The cells are routinely tested for mycoplasma contamination and were tested negative all times.
Commonly misidentified lines<br>(See ICLAC register)	Commonly misidentified lines were not used.
